# Plant derived biopesticides for controlling imported fire ants (*Solenopsis* spp.): a comprehensive review on biochemical traits, promising alternatives, and mechanisms of action

**DOI:** 10.3389/fpls.2026.1848932

**Published:** 2026-07-06

**Authors:** Maricruz Rangel-Galván, Yesenia Ithaí Ángeles-López, Nemesio Villa-Ruano

**Affiliations:** 1 SECIHTI-Dirección de Innovación y Transferencia de Conocimiento, Benemérita Universidad Autónoma de Puebla, Puebla, Mexico; 2 Facultad de Ciencias Biológicas, Benemérita Universidad Autónoma de Puebla, Puebla, Mexico

**Keywords:** acetylcholinesterase, biopesticides, essential oils, glutathione S-transferase, odorant-binding proteins, *Solenopsis* spp

## Abstract

The genus *Solenopsis* is also known as imported fire ants and comprises several invasive species, including the highly aggressive *Solenopsis invicta* (RIFA). Imported fire ants have spread from South America to every continent due to international trade and climate change, affecting agriculture, ecosystems, and even human health. Conventional chemical insecticides are widely used for controlling *Solenopsis* spp., however, their application results in environmental and health risks, including toxicity to non-target organisms. These concerns have led to growing interest in biopesticides as a safer and more sustainable alternative. This review examines the effects of plant-derived biopesticides on *Solenopsis* spp., with particular emphasis on RIFA as a model system and essential oils and their main volatiles as a safe agroecological control alternative. Overall, a solid background suggests that azadirachtin, eugenol, carvacrol, citral, and isothiocyanates induce rapid mortality and behavioral disruption in fire ants, often through blocking acetylcholinesterase (AChE) and glutathione S-transferase (GST). In addition to these enzymatic targets, growing evidence highlights the role of the chemosensory system, affecting odorant receptors and semiochemical perception. Twenty-four odorant-binding proteins (OBPs) and 23 chemosensory proteins (CSPs) have been identified in RIFA, with differential expression depending on factors such as caste, tissue, foraging activity, and corpse removal. The current state of the art suggests that bait strategies combining toxicants with attractants or repellents may be considered to improve the control efficiency of RIFA. Although biopesticides exhibit low or negligible risks to non-target organisms and the environment, their efficacy, stability, and large-scale application face evident challenges in the short term. Understanding the roles of essential oils and their active compounds may contribute to more effective and sustainable control of imported fire ants.

## 1 Introduction

The *Solenopsis* genus includes several invasive species considered highly destructive pests worldwide, collectively referred to as imported fire ants (Angulo et al., 2022). Among them, *Solenopsis invicta* is the most representative and aggressive member, commonly known as red imported fire ant (RIFA) (EFSA Panel on Plant Health et al., 2023). Native to South America, RIFA have achieved new niches in the United States, China, Australia, India, Japan, South Korea, and Vietnam. In particular, RIFA has substantially spread across several parts of the world because of intense international trade, cargo container traffic, and climate change (Wylie et al., 2020; Li et al., 2023). RIFA represents a serious threat to agriculture, ecosystems, economies, and human health (Chan and Guénard, 2020) due to its aggressive behavior in plant crops and rapid expansion (Kenfack et al., 2022). Extensive spread of RIFA disrupts the ecosystem balance, inducing loss of native invertebrate communities (Epperson et al., 2020), egg reptile predation (Swartwout and Willson, 2022), bobwhite (*Colinus virginianus*) population declining (Redmond et al., 2022), and allergic and/or anaphylactic reactions caused by fire ant venom (Angulo et al., 2022). RIFA invasion caused losses of $10.95 billion from 1930 to 2020, with a projected loss of $40.98 billion through 2084. Most of these losses are associated with the invasion of RIFA and *Wasmannia auropunctata* (little fire ant) in the United States of America and Australia (Angulo et al., 2022). These ecological and economic pressures have intensified the search for effective management strategies. It is known that fire ant behavior is largely regulated by chemical communication. Fire ants exhibit a social structure composed of workers, males, and queens, in which pheromones and other semiochemicals released by insects, microorganisms, and plants modulate communication and behavior (Wilson, 1978). These chemical signals convey information about the colony and its surrounding environment. Anatomical structures such as antennae, together with specialized proteins, neurons, and specific brain regions, constitute the sensory circuit involved in odor perception (Rana et al., 2024). Trail, alarm, queen, and necrophoric pheromones contribute to the organization, defense, and attack behaviors of *Solenopsis* species (Morgan, 2008). Accordingly, disrupting chemosensory perception represents a promising strategy for targeted behavioral manipulation in fire ant management. Despite the available information on the behavior of *Solenopsis* spp., their control has primarily relied on conventional hazardous pesticides (Rezende-Teixeira et al., 2022). Although some of these chemical insecticides can be effective (Song et al., 2024), their extensive use is still showing significant risks to human health and the environment, including adverse effects on non-target organisms, soil microbiota, and phreatic water (Rezende-Teixeira et al., 2022; Zhou et al., 2024). Pesticide dissemination in water and foods has been associated with cancer, acute hepatic toxicity, neurodegenerative diseases, respiratory diseases, and reproductive disorders, among other adverse effects (Zhou et al., 2024). These limitations have driven increasing interest in biopesticides as a safer and more sustainable alternative for pest control (Rizvi et al., 2025).

Biopesticides derived from plants, bacteria, and fungi exhibit relatively low toxicity for non-target organisms (Reyes-Ávila et al., 2024; Hezakiel et al., 2024). Among these alternatives, plant-derived products such as essential oils and their volatile constituents have received considerable attention due to their rapid bioactivity, biodegradability, and capacity to disrupt insect behavior (Chen and Oi, 2020). Several studies have demonstrated the bioactive potential of plant extracts and essential oils against the genus *Solenopsis* (Chen and Oi, 2020). For example, extracts of *Artemisia annua* exhibit biocidal activity against *Solenopsis saevissima*, while powders and/or essential oils of clove, mint, cinnamon, coriander, eucalyptus, and pepper affect *Solenopsis germinata* (Seixas et al., 2018; Nalini and Sasinathan, 2020). Similarly, hybrid imported fire ants such as *Solenopsis invicta* and *Solenopsis richteri* seem to be susceptible to the *Magnolia grandiflora* essential oil (Ali et al., 2022). Many of these plant-derived compounds interfere with key enzymatic systems involved in neurotransmission and detoxification, including acetylcholinesterase (AChE) and glutathione S-transferase (GST) (Xie et al., 2020; Du et al., 2020). In addition, some compounds disrupt semiochemical perception, further affecting colony-level behavior (Du and Chen, 2021). However, one evident challenge for essential oil-based biopesticides is the need for sustainable, scalable production to enable widespread field application (Chen and Oi, 2020; Nabi et al., 2025). Despite the existence of several plant-derived compounds against fire ants, current knowledge remains fragmented across studies on toxicity, repellency, or molecular targets, with limited integration. Therefore, this review proposes an integrative conceptual framework that links plant-derived semiochemicals, olfactory disruption, biochemical targets, and colony-level behavioral responses as interconnected components for the sustainable management of imported fire ants (*Solenopsis* spp.). By combining evidence from toxicological, behavioral, and molecular studies, this framework shifts the perspective from conventional toxicity-based control toward behaviorally informed and semiochemical-oriented management strategies. A special emphasis is placed on the role of olfaction in fire ant behavior, the perception of semiochemicals associated with essential oils, and the ecological implications of plant volatiles for target ants and non-target insects.

## 2 Materials and methods

This review was conducted following a systematic literature search focused on plant-derived biopesticides against *Solenopsis* spp. Literature retrieval was performed primarily using Google Scholar and ISI Web of Knowledge due to their broad coverage of studies on chemical ecology, entomology, natural products, and agroecological pest management. Depending on the thematic scope of each section, both classical and recent publications were included. For section 3, we select studies addressing fire ants’ chemical communication and social structure, semiochemical and olfactory protein interactions, and the impact on fire ants’ behaviour. Keywords included combinations of “*Solenopsis*”, “chemosensory proteins”, “odorant-binding proteins”, “olfactory proteins”, “olfactory system”, “castes”, “chemical communication”, and “ecological interactions”. For sections 4, searches focused on information on biocides as an ecological control management of *Solenopsis* spp. Search terms included combinations of “*Solenopsis*”, “fire ants”, “essential oils”, “plant volatiles”, “biopesticides”, “fumigant activity”, “repellent activity”, and “chemical ecology”. Because these sections aimed to provide a broad overview of the current state of knowledge, no strict publication date restriction was applied. For Section 5, the search strategy prioritized studies addressing molecular and biochemical mechanisms associated with plant-derived compounds in *Solenopsis* spp. Search terms included “mechanism of action”, “acetylcholinesterase inhibition”, “ionic channels”, “glutathione S-transferase”, “G-protein receptors”, “chemosensory proteins”, and “*Solenopsis*”. Studies reporting experimental evidence of enzymatic inhibition or chemosensory interference were prioritized and integrated with mortality and repellency data. Section 6 focused on the relationship between biopesticides active against *Solenopsis* spp. and their effects on beneficial non-target insects, particularly pollinators. Keywords included “pollinators”, “*Apis mellifera*”, “non-target insects”, “ecotoxicology”, “essential oils and bees”, and “botanical insecticides and pollinators”. Sections 7 and 8 emphasized recent studies on formulation technologies, field applications, environmental stability, and scaling strategies, using terms such as “nanoencapsulation”, “controlled release”, “bait systems”, “field application”, “semiochemical analogs”, “delivery systems”, “essential oil production”, and “integrated pest management”. In these sections, priority was given to studies published from 2014 onwards because they better reflect current translational challenges and emerging strategies for the large-scale application of plant-derived biopesticides. Studies were included when they evaluated plant-derived extracts, essential oils, or isolated phytocompounds against *Solenopsis* spp. and reported mortality, repellency, behavioral disruption, fumigation, enzymatic inhibition, or olfactory-related effects under laboratory, semi-field, or field conditions. Studies unrelated to *Solenopsis* spp., focused exclusively on synthetic insecticides, or lacking experimental evidence relevant to the objectives of this review, were excluded. Selected studies were manually screened and categorized according to mortality, repellency, chemosensory mechanisms, enzymatic targets, ecological implications, and translational strategies for field applications. Comparative synthesis was prioritized to identify recurrent phytochemical classes, shared mechanisms of action, and limitations that hinder the transition from laboratory efficacy to scalable agroecological applications.

## 3 *Solenopsis* spp. biology

### 3.1 *Solenopsis* spp. behavior

Fire ants are eusocial insects divided into queens, workers, and male castes that perform specific tasks within their colony or nest (Wilson, 1978). Workers are a non-reproductive caste and constitute the largest population (Wilson, 1978). Age and size (e.g., major, medium, minor) determine whether workers serve as nurses, caretakers, foragers, explorers, patrollers, or soldiers. Considering the complexity of their social structure, communication is essential for maintaining the operation of ant colonies (Morgan, 2008). Semiochemicals such as pheromones and cuticle components (e.g., hydrocarbons and fatty acids) serve as cues in RIFA ants’ coordination to exert a variety of tasks (Brindis et al., 2008; Zeng et al., 2022; Zeng, 2023). In addition, ants use environmental information to find food and identify threats or allies by detecting other semiochemicals, such as volatile organic compounds emitted by plants, microorganisms, and other insects. Moreover, several ant pheromones are volatiles that disperse through the air to establish chemical communication (Brindis et al., 2008; Zeng et al., 2022; Zeng, 2023).

RIFA produces pheromones associated with different behaviors, including foraging, mating, defense against external threats, dismembering ant corpses, removing ant corpses (necrophoric behavior), and queen identification (Morgan, 2008; Suckling et al., 2010; Zeng, 2023). Over the last few decades, significant progress has been achieved on elucidating how ants’ antennae detect semiochemicals and how these substances affect RIFA’s physiology and behavior (Yan and Liebig, 2021). For example, alarm pheromones released by a single or several colony members stimulate the recruitment of a large number of workers (Wilson, 1978). RIFA workers exhibit a rapid alarm response to the alkylpyrazine, 2-ethyl-3,5-dimethylpyrazine, which is the main pheromone constituent. This pheromone stimulates the antenna at concentrations of 10 and 100 ng (Guan et al., 2014; Sun et al., 2017; Li et al., 2019). Similarly, two isomers of the trial pheromone *Z,E*- and *E,E*-α-farnesene, mediated the aggregation of RIFA workers. Low doses of *Z,E*-α-farnesene produce an attractant response, whereas higher doses exert a contrary effect (Xu et al., 2021). In addition, allofarnesene isomers triggered a similar behavior as that of farnesene isomers in fire ants, but they are not formally classified as pheromones yet (Alvarez et al., 1987; Xu et al., 2023a). The mixture of *cis*- and *trans*-allofarnesene applied to baits was a stronger attractant agent (68% at10 µg/mL) for RIFA’s minor workers, whereas 2,5-dimethyl-3-pyrazine was significantly less attractive. However, trail pheromone attraction decreased (50% at 10 µg/mL) at large distances and longer times (Gokulanathan et al., 2024). In addition to trail and alarm pheromones, bioinsecticide formulations (e.g., baits, nanoparticles) may enhance worker recruitment and aggregation, thereby increasing fire ant mortality. Adjusting the proportions of pheromone concentrations, determining the effective duration, and the action ratio may help establish the frequency of applications in urban and field conditions, increasing formulation efficacy for controlling fire ant populations.

RIFA workers use pheromones to establish temporal or long-term mutualism with plants and other insects, enhancing their fitness (Xu and Chen, 2021; Travanty et al., 2022). For instance, RIFA workers prevent the dispersion of the aphid *Aphis gossypii* by releasing *Z,E*-α-farnesene and *E,E*-α-farnesene. Additionally, *Z,E*-α-farnesene increased aphid reproduction rate (Xu et al., 2021). Large aphid populations provide a sustained source of honeydew for RIFA workers, while the ants in turn protect the aphids from predators and other threats (Xu et al., 2021). Furthermore, semiochemicals released by the associated bacteria from the *Phenacoccus solenopsis* honeydew were stronger attractants for RIFA workers (Zhang et al., 2023a). *Brachybacterium* and *Kouria* produced limonene in honeydew, while *Brachybacterium* and *Microbacterium* produced phenylethyl alcohol. Interestingly, different concentrations (0.1 µg/mL, 1 µg/mL, and 10 µg/mL) of limonene, phenylethyl alcohol, and 2,4-ditert-butylphenol were attractive to RIFA workers, showing an opposite effect at traceable concentrations (0.01 µg/mL and 0.001 µg/mL) (Zhang et al., 2023a). Beyond trophic interactions, RIFA disrupts plant-pollinator ecological interactions. The detection of workers’ cuticular hydrocarbons by *Aphis cerana* and *Pieris rapae* interrupts the pollination of RIFA-infested flowers of *Brassica napus* (Wu et al., 2016). A recent study indicates that compounds released by the soil bacterium *Arthrobacter woluwensis* attract RIFA workers and stimulate their digging behavior. In contrast, bacteria of the genus *Bacillus*, *Paenibacillus*, and *Brevibacillus* produce compounds that repel RIFA workers (Travanty et al., 2022). Microbial and insect semiochemicals that significantly affect fire ants’ digging behavior are strong candidates for plant-derived repellent formulations, as hindering the construction of fire ant nests or disrupting ecological interactions can help limit their spread. However, to design effective anti-digging formulations, it will be necessary to test whether soil type and depth affect semiochemical delivery. Also, evaluating the digging and tunnel construction rates of fire ants is needed to determine the anti-digging properties of these formulas. Additionally, the collateral effects of these formulations on ecological interactions, such as pollination rates in urban, agricultural, and field environments, should be further studied to determine side effects.

### 3.2 Perception mechanisms of odorant receptors

**Figure 1:**
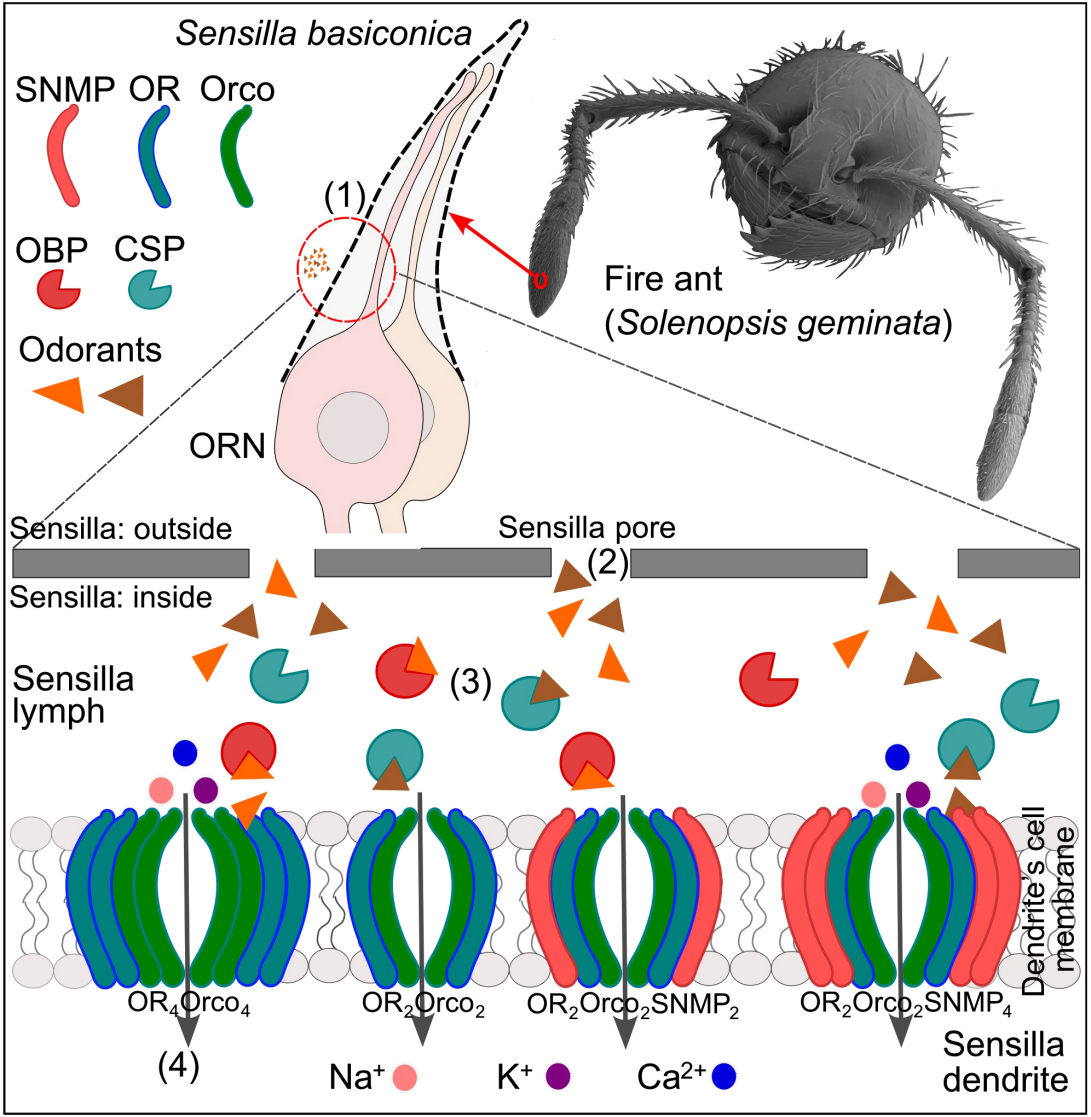
Proposed olfactory sensing of odorants in *Solenopsis* spp. by sensorial organs. (1) *Sensilla basicona* are located in the antennae of fire ants and are innervated by dendrites of the ORNs. (2) Odorants, such as pheromones and semiochemicals from plants and insects, pass through the sensilla pore. (3) The sensilla lymph contains OBPs and CSPs that solubilize and transport odorants to the multimer complexes of ORs, Orco, and SNMP, forming ion channels. (4) Odorants bind to the multimer complexes that transport calcium, sodium, and potassium ions. ORNs' axons connect to the brain, where information is interpreted to trigger behavioral or physiological responses.

Semiochemicals are odorant molecules detected by the ants’ olfactory organs. The antennae’s sensilla harbor neurons and molecular machinery for signal transmission and interpretation in the brain (Rana et al., 2024). The coeloconica, ampullacea, tricodea, tircodea curvata, and basicona sensilla of RIFA are involved in odorant traffic (Renthal et al., 2003). A large number of genes coding for odorant-binding proteins (OBPs), chemosensory proteins (CSPs), and odorant receptors (ORs) have been found in the RIFA’s genome (Zhang et al., 2016; Wanchoo et al., 2020). The OBPs and CSPs are suspended in the sensilla’s lymph and transport the odorants from the antennae pores to ORs and OR co-receptors (Orco). ORs and Orco are located on the dendrites of the cilia, which are attached to the odorant-receptor neurons (sensory neurons, ORNs) that innervate the sensilla (**Figure 1**) (Wicher and Miazzi, 2021; Rana et al., 2024). Ionotropic receptors, gustatory receptors, sensory neuron membrane proteins, and odorant-degrading esterases, including antennae-specific cytochrome P450 proteins, complement the intricate olfactory system of RIFA (Yan and Liebig, 2021; Wicher and Miazzi, 2021; Rana et al., 2024). Once the odorants are interpreted in the brain, they are transduced into particular physiological or behavioral responses (Yan and Liebig, 2021).

#### 3.2.1 Odorant-binding proteins

OBPs are small carrier proteins from 130 to 150 amino acids that include pheromone-binding proteins and general odorant-binding proteins. The RIFA genome has at least twenty-four OBPs genes (herein *Si*OBPs) and one pseudogene. Except for the *Si*OBP17 gene, the rest showed differential expression in the antennae, head, thorax, and abdomen of workers, queens, and males (Gotzek et al., 2011; Zhang et al., 2016; Pracana et al., 2017; Shah and Renthal, 2020). OBPs are categorized as classical, c-minus, and c-plus based on the distribution and content of cysteine ​​residues (Zhang et al., 2016). These differential expression patterns highlight OBPs as potential molecular targets for disrupting caste-specific communication pathways, causing foraging coordination impairment and loss of colony defense. High expression levels of *Si*OBP1, *Si*OBP5, *Si*OBP6, and *Si*Orco were observed in the antenna of RIFA workers (Du and Chen, 2021). *Si*Orco and *Si*OBP5 proteins detected the alarm pheromone 2,4,6-trimethylpyridine, but not 2-ethyl-3,6-dimethylpyrazine. Gene silencing of *Si*Orco and *Si*OBP5 through dsRNA injection (RNA interference) reduced gene transcription at 72 hours post-treatment and disrupted detection of 2,4,6-trimethylpyridine according to electroantennography assays (Du and Chen, 2021). Interestingly, *Si*OBP1 was highly expressed in the antennae of worker ants, and its protein was identified in the antennae proteome of males and workers (González et al., 2009; Zhang et al., 2016; Shah and Renthal, 2020; Du and Chen, 2021; Shen et al., 2025). The binding of anethole, 1S-(-)-β-pinene, linalool, myrcene, benzaldehyde, α-pinene, β-caryophyllene, and 2,5-dimethylhexane to the *Si*OBP1 protein was predicted through an *in silico* approach and confirmed through experimental methods (Shen et al., 2025). From tested volatiles, *Si*OBP1protein exhibited the highest affinity for β-caryophyllene (0.1 µg/µL) and the lowest affinity for 2,5-dimethylhexane (0.1 µg/µL) (Shen et al., 2025). RIFA workers were strongly attracted to a mixture of anethole and 1S-(-)-β-pinene. In contrast, the combination of anethole and β-caryophyllene was less appealing (Shen et al., 2025). Baits formulated with high concentrations (1 µg/µL) of assayed volatiles were attractive to RIFA workers. In contrast, baits with lower concentrations (0.1 µg/µL) of anethole, 1S-(-)-β-pinene, and β-caryophyllene were repellent (Shen et al., 2025). Workers’ attraction to anethole, 1S-(-)-β-pinene, β-caryophyllene, α-pinene, and linalool was significantly reduced when the *Si*OBP1 gene was silenced via RNAi (Shen et al., 2025). The concentration-dependent shift from attraction to repellency suggests that fine-tuning volatile blends is needed in push–pull strategies to manipulate RIFA foraging behavior and direct fire ant movement without relying on broad-spectrum insecticides.

On the other hand, *Si*OBP2 and *Si*OBP14 modulate workers’ foraging behavior (Xiao et al., 2024; Liu et al., 2025). *Si*OBP2 was expressed in the *sensilla basiconica* of the RIFA workers’ antennae and was involved in detecting the alarm pheromone 2,4,6-trimethylpyridine, while *Si*OBP14 was expressed in the antennae of workers and males (Zhang et al., 2016; Shah and Renthal, 2020; Wei et al., 2021; Xiao et al., 2024; Liu et al., 2025). The gene silencing of the *Si*OBP2 gene via RNAi disrupted OBP2 protein binding to the bait sausage odorants 3-mercapto-2-butanone and furfuryl mercaptan in accordance with electroantennography assays (Wei et al., 2021; Xiao et al., 2024; Liu et al., 2025). Similarly, *Si*OBP14 gene silencing (RNAi) led to a shift from attraction to repellency towards 2-methyltetrahydrofuran-3-one, 2-methyl-3-furanthiol, 4,5-dimethylthiazole, and 2-methyl-3-methyl(thio)furan released by bait sausages for RIFA workers (Zhang et al., 2016; Wei et al., 2021; Xiao et al., 2024). Consequently, *Si*OBP2 and *Si*OBP14 gene repression significantly impaired the bait-sausage-searching efficiency of RIFA workers (Xiao et al., 2024; Liu et al., 2025). These findings indicate that OBP-mediated odor detection is crucial for bait recognition, endorsing that semiochemical-based management enhances bait selectivity, reducing side effects.

The general protein 9 (Gp-9) or *Si*OBP3 determines the social structure of RIFA colonies. Gp-9 is expressed in the antennae of males and the antennae and tibiae of workers (Krieger and Ross, 2005; Cohanim et al., 2018; Shah and Renthal, 2020). Two Gp-9 haplotypes, SB and Sb, are encoded from a non-recombining region named the “social chromosome” that comprises 504–600 genes (Cohanim et al., 2018). Colonies only containing SB haplotypes are monogynous, led by a single queen. In contrast, polygynous colonies with Sb haplotypes are led by several queens. It is believed that Gp-9 regulates the workers’ behavior toward queens. Workers from colonies with SB/SB or SB/Sb haplotypes accept queens with SB/Sb haplotypes but kill those with SB/SB haplotype (Dang et al., 2019). This evidence suggests that workers differentiate between queens with SB/SB or SB/Sb haplotypes using olfactory proteins specifically encoded in the Sb haplotype. The transcriptome of workers’ antennae from SB/SB, SB/SB, or SB/Sb colonies revealed differential expression of *Si*OR463, *Si*OBP12, and 11 different genes across the three groups (Dang et al., 2019). The *Si*OBP12 paralog, annotated as *Si*OBP12’, is exclusively found in the Sb haplotype of the RIFA species, being absent in the Sb haplotype of *Solenopsis geminata*. Both paralogs were expressed throughout the worker’s body, but *Si*OBP12 was not expressed in the antennae. This fact suggests that queen discrimination in RIFA could be attributed to the neofunctionalization of duplicated olfactory genes (Dang et al., 2019). Thus, understanding the molecular basis of queen discrimination and social organization could open new alternatives for destabilizing colony structure, particularly in polygynous populations that are typically more invasive and aggressive. A possible approach is to inhibit Gp-9 and SiOBP12 to interrupt queen recognition by workers, which could lead to queen elimination and provoke colony collapse.

Sanitation behavior and social immunity within RIFA nests are regulated by the *Si*OBP15 protein (Zhang et al., 2025). Worker ants dismember and remove microbial-infected corpses to maintain the sanitation in the nest. When workers were inoculated with *B. bassiana* (1 x 10^8^ conidia/mL), the expression of *Si*OBP15 significantly increased between 24 and 72 hours post-inoculation. Silencing *Si*OBP15 through RNAi reduced workers’ ability to dismember infected ant corpses (Wei et al., 2021; Zhang et al., 2025). Cuticular composition is an indicator of nest health, and this parameter can be monitored by specific OBPs expression. Behenic acid, 3-octenol, and 3-ethyltetrahydro-2-furanone were more abundant in the cuticles of workers infected by specific fungi. In contrast, the levels of acetoin, 2,3-butanedione, and trans-11-eicosenoic acid decreased, while the amounts of oleic acid and linolenic acid remained similar (Zhang et al., 2025). Behenic acid released from worker corpses (dead from frostbite or *B. bassiana*-infected) seems to play a crucial role in triggering dismembering activity (Zhang et al., 2025). Conversely, oleic acid and *cis*, *cis*-9,12-linolenic acid inhibited the dismemberment process. The *Si*OBP15 protein interacts with the proteins encoded by *Si*ApoLp-III and *Si*FABP5 genes, which are involved in the insect’s innate immunity and help to regulate dismemberment behavior (Zhang et al., 2025). Collectively, these findings highlight OBPs involved in sanitation behavior as potential molecular targets for disrupting social immunity and chemical communication in RIFA colonies.

Another study showed that limonene, nonanal, and 2,4,6-trimethylpyridine bind to *Si*OBP15 and *Si*OBP7 proteins, while 2-ethyl-3,5-dimethylpyrazine binds to the *Si*OBP7 protein (Yang et al., 2022). When RIPA workers were exposed to cadmium, there was a reduction in the expression levels of the SiOBP7 (100, 300, and 500 mg/L), *Si*OBP9 (100, 300, and 500 mg/L), and *Si*PBP15 (100 mg/kg) genes (Yang et al., 2021). Electroantennographic assays indicated that cadmium exposure diminished the capacity of *Si*OBP7 and *Si*OBP15 proteins to bind limonene, nonanal, and 2,4,6-trimethylpyridine, while increasing their sensitivity to undecane. Gene silencing of *Si*OBP7 and *Si*OBP15 through RNAi dismissed workers’ ability to perceive nonanal and undecane (Yang et al., 2022) and indicates that cadmium can impact the expression level in several OBPs and thus the behavior in ants.

Similarly, cadmium exposure and *Beauveria bassiana* (1 x 10^8^ conidia/mL) infection reduced the expression levels of *Si*OBP14 in the RIFA workers’ antennae (Yang et al., 2021; Wei et al., 2021). These observations indicate that fungal infection and the stress triggered by metal contamination may interfere with odorant perception by RIFA (**Table 1**). On the other hand, cadmium exposure induced high expression of *Si*OBP12 in workers’ antennae (Yang et al., 2021). Importantly, exposure to cadmium offset the behavioral responses elicited by 2,4,6-trimethylpyridine and 2-ethyl-3,5-dimethylpyrazine in RIPA workers (Yang et al., 2021). Overall, these studies showed that cadmium alters RIFA behavior by disrupting the transmission of chemical signals, highlighting the crucial role of the olfactory system in social structure. This evidence points out OBPs as targets for plant-derived biopesticides and repellents. We considered that the delivery systems of these biopesticides and repellents will require efficacy evaluation in contaminated environments to account for potential malfunction.

**Table 1:** Odorant-binding proteins of *Solenopsis invicta* that are involved in behavioral responses and semiochemicals detection.

Gene/protein	Organ or tissue	Caste	Binding compounds and putative role	Reference
SiOBP1	Antennae, head, thorax, abdomen, and tibia	Workers, female and alate male	Binds to β-caryophyllene, anethole, 1S-(-)-β-pinene, linalool, myrcene, benzaldehyde, α-pinene, and 2,5-dimethylhexane	Shen et al., 2025; Du and Chen, 2021; Zhang et al., 2016
SiOBP2	Antennae, head, thorax, and abdomen	Workers, female and alate male	Binds to 3-mercapto-2-butanone and furfuryl mercaptan from sausage bait. Involved in foraging behavior	Liu et al., 2025; Shah and Renthal, 2020; Zhang et al., 2016
SiOBP3	Antennae, head, thorax, and abdomen	Workers, female and alate male	Essential in the social structure of the colony	Shah and Renthal, 2020; Cohanim et al., 2018; Zhang et al., 2016; Krieger and Ross, 2005
SiOBP5	Antennae, head, thorax, abdomen, and tibia	Workers, female and alate male	Bind to 2,4,6-trimethylpyridine Its silencing disrupts the alarm response and communication	Du and Chen, 2021; Shah and Renthal, 2020; Zhang et al., 2016
SiOBP7	Antennae, head, thorax, abdomen, and tibia	Workers, female and alate male	Bind to limonene, nonanal, 2,4,6-trimethylpyridine, and 2-ethyl-3,5-dimethylpyrazine Low sensitivity and expression after cadmium exposure	Yang et al., 2022; Yang et al., 2021; Shah and Renthal, 2020; Zhang et al., 2016
SiOBP9	Antennae, head, thorax, abdomen (all body)	Workers, female and alate male	Bind to 2,4,6-trimethylpyridine, and 2-ethyl-3,5-dimethylpyrazine	Yang et al., 2021; Zhang et al., 2016
SiOBP12	Antennae, head, thorax, abdomen (all body)	Workers, female and alate male	High expression after cadmium exposure Involved in queen discrimination by workers	Yang et al., 2021; Dang et al., 2019; Zhang et al., 2016
SiOBP14	Antennae, head, thorax, abdomen (all body)	Workers, female and alate male	Bind to 2-methyltetrahydrofuran-3-one, 2-methyl-3-furanthiol, 4,5-dimethylthiazole, and 2-methyl-3-methyl(thio)furan from food baits Involved in foraging behaviour and chemosensory signalization in ORNs (sensitive to cadmium exposure)	Xiao et al., 2024; Shah and Renthal, 2020; Zhang et al., 2016
SiOBP15	Antennae, head, thorax, abdomen, and tibia	Workers, female and alate male	Binds to limonene, nonanal, undecane, and 2,4,6-trimethylpyridine Binds to cuticular components such as behenic, oleic, and cis, cis-9,12-linoleic acids Involved in the dismemberment of corpses	Zhang et al., 2025; Shah and Renthal, 2020; Yang et al., 2022; Zhang et al., 2016

*Si*OBP4, *Si*OBP6, *Si*OBP8, *Si*OBP9-*Si*OBP11, *Si*OBP13, *Si*OBP16, and *Si*OBP17 have not yet been associated with specific olfactory functions. Nevertheless, *Si*OPB4, *Si*OBP11, *Si*OBP12, and *Si*OBP16 showed high expression in the antennae of RIFA workers exposed to cadmium (Yang et al., 2021). *Si*OPB6 and *Si*OBP11 were expressed in the antennae of worker ants, whereas *Si*OBP4 and *Si*OBP16 were only expressed in the tibia of the same caste (Shah and Renthal, 2020; Du and Chen, 2021). *B. bassiana* infection significantly reduced *Si*OBP4 and *Si*OBP6 expression from 24 to 48 h post-inoculation (Wei et al., 2021). Interestingly, OBP6 protein did not bind to the pheromones 2-ethyl-3,5-dimethylpyrazine and 2,4,6-trimethylpyridine (Wei et al., 2021). The lack of functional characterization for several SiOBPs represents a critical knowledge gap that may conceal yet-unexplored targets for semiochemical-based management. Although OBPs mediate the initial stages of odorant recognition, effective semiochemical disruption also depends on downstream receptors and signaling components, which are discussed in the following sections.

#### 3.2.2 Chemosensory proteins

CSPs are a family of small soluble proteins similar to OBPs that help to trap and transport odorants suspended in the lymph to the ORs and Orco complexes. Several CSP genes are expressed in several tissues, including RIFA antennae, indicating that they perform diverse functions beyond olfaction (Cheng et al., 2015). Up to 24 genes encoding CSPs (*Si*CSPs), along with 2 pseudogenes, have been identified in RIFA (González et al., 2009; Kulmuni et al., 2013; Shah and Renthal, 2020). Remarkably, 21 genes were differentially expressed in the antennae, head, thorax, and abdomen of alate males, alate females, and workers (Wanchoo et al., 2020). However, only fourteen *Si*CSP genes were expressed in the antennae or tibiae of worker ants and the antennae of male ants, whereas thirteen CSP proteins were detected in the proteome of worker antennae (González et al., 2009; Shah and Renthal, 2020). The *Si*CSP1 gene is more highly expressed in the legs and head of RIFA workers than in the same organs from alate males and females (**Table 2**) (Cheng et al., 2015; Qiu and Cheng, 2017; Shah and Renthal, 2020). Polar cuticular lipids of workers bind to the *Si*CSP1 protein, contributing to nestmate recognition, while fatty acids from decaying corpses trigger a necrophoric behavior (González et al., 2009; Cheng et al., 2015). Disruption of *Si*CSP1-mediated corpse recognition could compromise nest sanitation efficiency, potentially increasing colony susceptibility to pathogens or reducing social cohesion. Gene silencing of *Si*CSP1 via siRNA in workers decreased the corresponding expression levels and reduced oleic and linoleic acid sensitivity according to electrophysiological assays. Consequently, workers removed fewer corpses than in normal conditions (Qiu and Cheng, 2017). In contrast, *Si*CSP9 was highly expressed in RIFA alate females than in other cases. This expression was primarily observed in the abdomen as well as in the antennae and tibia of males and workers (**Table 2**) (Qiu and Cheng, 2017; Shah and Renthal, 2020). In addition, *Si*CSP9 was highly expressed in the third-instar larvae, and its silencing via RNAi resulted in a high mortality rate. The *Si*CSP9 protein was putatively involved in fatty acid biosynthesis and the molting process, nevertheless, further studies are needed to confirm these functions (González et al., 2009; Qiu and Cheng, 2017). The high *Si*CSP9 expression in larvae and the corresponding RNAi-associated mortality unravel this gene as a possible regulator of developmental stability, suggesting that any change at this stage may impact colony renewal dynamics. Simultaneously, these findings indicate that CSPs contribute not only to chemical communication but also to behavioral and developmental regulation, expanding the range of potential molecular entry points for disrupting colony functionality.

**Table 2:** Chemiosensory proteins and other olfactory proteins of *Solenopsis invicta* that are involved in behavioral responses and semiochemicals detection.

Gene/protein	Organ or tissue	Caste	Observations	Reference
SiCSP1	Antennae, head, thorax, abdomen	Workers, female and alate male	Binds to polar cuticular lipids of workers Involved in nestmate and non-nestmate recognition, and modulate necrophoric behavior	Wanchoo et al., 2020; Qiu and Cheng, 2017; González et al., 2009
SiCSP9	Antennae, head, thorax, abdomen	Workers, female and alate male	Involved in the moulting process	Wanchoo et al., 2020; Cheng et al., 2015; González et al., 2009
SiOrco	Antennae	Workers	Bind to 2,4,6-trimethylpyridine Its silencing disrupts the alarm response and communication Interact with SiSNMP1 and ORs in the antenna	Du and Chen, 2021; Shah and Renthal, 2020
SiOR463	Antennae	Workers	Only present in the SB haplotype	Dang et al., 2019
SiOR88	Antennae	Workers	Putatively involved in the social structure or the queen's discrimination	Cohanim et al., 2018
SiOR89	Antennae	Workers	Putatively involved in the social structure or the queen's discrimination	Cohanim et al., 2018
SiSNMP1	Antennae	Workers and males	Interact with Orco and ORs in the antennae	Du and Chen, 2021; Shah and Renthal, 2020
SinvCYP4V2	Antennae	Workers	Sensing and recognition of the alarm pheromone 2-ethyl-3,5-dimethylpyrazine	Jiang et al., 2025
SinvCYP6K1	Antennae	Workers	Sensing and recognition of the alarm pheromone 2-ethyl-3,5-dimethylpyrazine	Jiang et al., 2025
SiCYPs	Antennae	Workers and males	25 CYPs were found exclusively in the antennae	Shah and Renthal, 2020

#### 3.2.3 Odor receptor proteins

ORs and Orco are proteins comprised of seven helical transmembrane segments. ORs form heteromeric dimers or multimers with Orco and other ORs, which are tagged as permeable calcium channels. Since both receptors interact with G proteins, their functions seem to be independent of each other (Wicher and Marion-Poll, 2018). Considering that OR–Orco complexes constitute the primary signal-transduction gateway for odor perception, functional interference at this level could significantly affect behavioral outputs such as recruitment, alarm signaling, or trail following (Wicher and Marion-Poll, 2018). Recent research estimates that over 400 OR genes are encoded in ant genomes. Up to 407 *Si*OR genes have been identified in the RIFA genome, while 214 *Sf*ORs were found in the genome of *Solenopsis fluxa* (Wurm et al., 2011; Saad et al., 2018; Cohen and Privman, 2020; Mier et al., 2022; Zhang et al., 2023b). After manual curation and re-annotation, 287 OR genes were confirmed (Mier et al., 2022). The high number of OR genes suggests a highly specialized chemosensory system involved in fine discrimination against environmental and colony-derived chemical cues. Interestingly, two OR genes located in the “social chromosome”, *Si*OR88 and *Si*OR89, are unique to the SB haplotype of the Gp-9 gene in RIFA. Fire ants with Sb and SB haplotypes only retain the SB copy, whereas both *Si*OR88 and *Si*OR89 genes lie outside the “nine-exon clade,” which has significantly expanded due to the duplication of OR genes (Cohanim et al., 2018). Notably, *Si*OR463, *Si*OR88, and *Si*OR89 have been linked to the social structure of RIFA colonies, including their ability to recognize queens (Cohanim et al., 2018; Wicher and Marion-Poll, 2018; Dang et al., 2019; Xu et al., 2023b).

On the other hand, the levels of the Orco protein were negligible in the tibia of RIFA workers (Du and Chen, 2021). Nevertheless, the levels of this protein were five times higher in the antennae of RIFA workers than in the same organs from male ants (Du and Chen, 2021). It is believed that the Orco protein is involved in the perception of 2-ethyl-3,5-dimethylpyrazine by the antennae of RIFA workers (Du and Chen, 2021). Recent evidence suggests that Orco, ORs, and SNMP1 can be grouped into complexes. Specific complexes may include Orco_2_OR_2_, Orco_4_OR_4_, Orco_2_OR_2_SNMP_2_, and Orco_2_OR_2_SNMP_4_ (Shah and Renthal, 2020). To corroborate that these multimeric complexes occur and are functional in the ORNs of RIFA, co-immunoprecipitation and patch-clamp electrophysiology techniques can be applied. The formation of multi-protein receptor complexes suggests additional regulatory layers that may fine-tune odor detection thresholds and ligand specificity.

Furthermore, two ionotropic receptor subunits were detected in the antennae of male and worker ants, which were categorized as IR25a-like proteins due to their similarity to the IR8a and IR25a ionotropic receptors in *Drosophila melanogaster* (Shah and Renthal, 2020). Remarkably, the specific functions of IR25a-like proteins in RIFA remain unknown. Then, the partial characterization of ionotropic receptors represents a significant gap that limits our understanding of their activation by semiochemicals and their participation in signal transduction in RIFA. Collectively, OBPs and CSPs mediate odorant capture and transport, whereas OR–Orco complexes and ionotropic receptors drive signal transduction and behavioral output. This multilayered chemosensory architecture underpins recruitment, alarm communication, and queen recognition in RIFA. Consequently, perturbations at different molecular levels may be translated into measurable effects on colony cohesion and adaptive performance.

#### 3.2.4 Cytochrome P450-mediated odorant inactivation in RIFA antennae

Antenna-specific cytochrome P450s (CYP) are part of the odorant-degrading esterase group that deactivates odorants (Lewis and Ito, 2008). These enzymes help to maintain olfactory sensitivity and temporal resolution during chemical communication. Twenty-five CYP genes (*Si*CYP) are exclusively expressed in the antennae of RIPA workers and males (Shah and Renthal, 2020). Comparison of CYP coding sequences from RIPA, *Spodoptera litura*, *Aphis mellifera*, and *Dendroctonus ponderosae* revealed five conserved regions. These five regions are Helix C (WXXXR), Helix I (GXE/DTT/S), Helix (EXXR), Meander (PXXFXPEX/DF), and heme-binding domain (PFXXGXRXCXG/A) (Jiang et al., 2025). The conservation of these structural motifs across insect taxa suggests evolutionary constraints linked to essential functions. The *Si*CYP6K1 and *Si*CYP4V2 genes were highly expressed in the antennae of RIFA workers and lower expressed in eggs, larvae, and nymphs (Jiang et al., 2025). The CYP6K1 protein belongs to the CYP3 subfamily and is putatively involved in the metabolism and detoxification of exogenous compounds in RIFA (Liang et al., 2024; Jiang et al., 2025). Similarly, the CYP4V2 protein belongs to the CYP4 subfamily and is probably involved in the biosynthesis of olfactory-related compounds (Yu et al., 2018; Jiang et al., 2025). Thus, antenna P450_S_ represents a crucial metabolic step in chemosensory regulation that modulates semiochemicals’ strength and incidence. Interestingly, it has been demonstrated that these proteins participate in sensing 2-ethyl-3,5-dimethylpyrazine (Jiang et al., 2025). RNAi gene silencing of *Si*CYP6K1 and *Si*CYP4V2 significantly reduced the antennae’s response to 2-ethyl-3,5-dimethylpyrazine, affecting RIFA workers’ orientation, speed, and range of movement (Jiang et al., 2025). This dual role in degradation and pheromone perception strongly suggests CYPs as modulators of signal intensity. Overall, the development of biopesticides for fire ants focuses on strategically disrupting their chemosensory systems and destabilizing their social structures. This can be achieved by targeting specific molecular gateways (e.g., OBPs, CSPs, ORs, and CYPs) that regulate colony cohesion or by leveraging semiochemicals to influence essential behaviors (e.g., foraging, queen recognition, and nest sanitation), making them more vulnerable to natural threats. Using chemoinformatic approaches, we could perform a structural analysis of chemosensory proteins to identify active sites and key binding residues for semiochemical binding. Also, conducting site-directed mutagenesis and heterologous expression systems could help identify key residues in binding pockets and the activation of receptor complexes (e.g., ORs-Orco) by specific semiochemicals.

## 4 Biopesticides for controlling *Solenopsis* spp.

### 4.1 Mortality

Plant-derived biopesticides have shown promising toxicity against *Solenopsis* spp. For instance, azadirachtin and celangulin from *Azadirachta indica* and *Celastrus angulatus* are effective biocide agents against major ants, showing median lethal dose (LD_50_) of 0.05-0.20 ng/ant after 24 h post-treatment (**Table 3**). Natural soils enriched with azadirachtin and celangulin (8 µg/g) produced 91% and 97% mortality after 72 h, respectively. The median lethal time (LT_50_) for these compounds under the same conditions was only 46 hours for azadirachtin and 33 hours for celangulin (Liang et al., 2023). In parallel, the essential oils of *Illicium verum*, *Blumea balsamifera*, *Acorus tatarinowii*, *Citrus limon*, *Mosla chinensis*, and *Cinnamomum cassia* exhibited high toxicity, causing 100% mortality within 24 hours at a concentration of 0.01 µL/cm³ (except for *C. limon*). Moreover, fumigation of *I. verum* and *B. balsamifera* essential oils at a concentration of 0.001 µL/cm³ produced 70% mortality after 12 days (Fu et al., 2023). Notably, limonene is a frequent constituent of many essential oils and is particularly abundant in *B. balsamifera* and *C. limon* (49%). Despite both essential oils containing limonene as a major constituent, their fungicidal effects were contrasting (Fu et al., 2023). This fact strongly suggested that the cytotoxic effect cannot be attributed solely to this compound but also to the synergic activity of other molecules dissolved in the volatile fraction (Fu et al., 2023). A similar trend was observed for the essential oil from *Citrus paradisi*, where limonene was the main constituent in crude grapefruit oil (GO1) and concentrated grapefruit oil (GO2), producing 70% and 74% mortality, respectively (Zhang et al., 2021). Nevertheless, after evaluation, octanal, α-pinene, β-phellandrene, d-carvone, and α-terpineol showed a stronger fumigant effect than limonene. These volatiles constituted around 3% of the total *C. paradisi* essential oil composition, suggesting that minor compounds substantially contribute to overall bioactivity (Zhang et al., 2021). Interestingly, GO2 induced 100% mortality within 7 hours, while GO1 achieved the same effect within 10 hours at a nominal fumigation dose of 5 µL per centrifuge tube (~25.5 µL/L air). Similarly, octanal produced 100% mortality at 6 hours, followed by α-pinene (100%), β-phellandrene (92%), d-carvone (88%), and α-terpineol (82%) at the same nominal dose after 8 hours post-treatment (Zhang et al., 2021). These findings suggest that synergistic interactions among volatile constituents substantially contribute to the bioactivity of essential oils. Consequently, preserving multicomponent blends may represent a more effective formulation strategy than isolated compounds, as these mixtures can enhance toxicity, stability, and behavioral disruption while potentially reducing the likelihood of resistance development (Hamdeni et al., 2026).

**Table 3:** Biocide parameters for potential plant biopesticides against *Solenopsis* spp.

Plant	Target	Mortality effect	Reference
Piper spp.	Solenopsis saevissima (Smith)	LC_50_ (48 h): Piper aduncum: 58 mg/L Piper marginatum types A: 122 mg/L Piper marginatum types B: 167 mg/L Piper divaricatum: 302 mg/L Piper callosum: 313 mg/L	Souto et al., 2012
Cupressus nootkatensis	Solenopsis invicta (Buren); Solenopsis richteri (Forel)	LC_50_ (48 h): nootka oil: 0.3 % LD_50_ (48 h): nootka oil/L: 8.9 µl	Addesso et al., 2017
Seriphidium brevifolium	Solenopsis invicta	LC_50_ (12 h): S. brevifolium oil: 16.5 µl/L (α+ β) Thujone: 19.1 µl/L 1,8-Cineole: 30 µl/L	Xie et al., 2020
Brassicaceae spp.	Solenopsis invicta	LC_50_ Allyl isothiocyanate: 32.5 µg/L 3-(Methylthio) propyl isothiocyanate: 57.6 µg/L LD_50_ Allyl isothiocyanate: 7.9 µg/ant 2-Phenylethyl isothiocyanate: 2.4 µg/ant 3-(Methylthio) propyl isothiocyanate: 2.1 µg/ant	Du et al., 2020
Magnolia grandiflora L.	Hybrid imported fire ants (Solenopsis invicta/Solenopsis richteri)	LC_50_ 1-Decanol: 140.6 µg/g 1-Octanol: 486.8 µg/g	Ali et al., 2022
Curcuma longa	Solenopsis invicta, Solenopsis richteri, Hybrids	LC_50_ (24-h): SI: Leaf essential oil: 85.8 µg/g Rizhome essential oil: 127.0 µg/g S. richteri: Leaf essential oil: 97.7 µg/g Rizhome essential oil: 109.9 µg/g Hybrids: Leaf essential oil: 182.7 µg/g Rizhome essential oil: 151.2 µg/g	Ali et al., 2024
Baccharis microdonta	Solenopsis invicta, Solenopsis richteri, Hybrids	LC_50_ (24 h): SI: 78.9 µg/g S. richteri: 97.5 µg/g Hybrids: 136.5 µg/g	Ali et al., 2023
Lippia gracilis	Solenopsis invicta	LD_50_: Lippia gracilis essential oil: 6.1 µg/mg Carvacrol: 6.8 µg/mg Carvacryl acetate: 6.9 µg/mg Carvacryl benzoate: 3.2 µg/mg Carvacryl butyrate: 7.5 µg/mg Carvacryl ethyl ether: 6.9 µg/mg Carvacryl hexanoate: 10.5 µg/mg Carvacryl isobutyrate: 6.5 µg/mg Carvacryl isovalerate: 10.8 µg/mg Carvacryl pvaloate: 5.9 µg/mg Carvacryl trichloroacetate: 7.9 µg/mg	Dantas et al., 2023
	Solenopsis invicta	LC_50_ (24 h): Eugenol: 0.020 mg/cm2 Isoeugenol: 0.003 mg/cm2 Methyl isoeugenol: 0.003 mg/cm2 Eugenol acetate: 0.004 mg/cm2 Methyl eugenol: 0.018 mg/cm2 Isoeugenyl acetate: 0.556 mg/cm2	He et al., 2023
Houttuynia cordata Thunb	Solenopsis invicta	LC_50_ (24 hr): 2-undecanone: 44.6 µg/g	Kurmanbayeva et al., 2023
Celastrus angulatus	Solenopsis invicta	LD_50_ (24 h): Azadirachtin: 0.20 ng/ant Celangulin: 0.05 ng/ant Veratramine: 544.6 ng/ant LT_50_ Azadirachtin: (1 mg/L) 7.7 h, (0.125 mg/L) 60 h Celangulin: (0.125 mg/L) 9.9 h, (0.031 mg/L) 54 h Veratramine (500 mg/L) 24 h, (200 mg/L) 47 h	Liang et al., 2023
Matricaria chamomilla	SI, Solenopsis richteri, Hybrids	LC_50_ M. chamomilla cv Egyptian 93.6 µg/g SI; 188.1 µg/g S. richteri M. chamomilla German CO2 98.1 µg/g SI; 138.4 µg/g S. richteri M. chamomilla cv. German 142.9 µg/g SI; 202.5 µg/g S. richteri α-bisabolol 159.23 µg/g SI.	Shah et al., 2023
Allium sativum	Solenopsis invicta	LC_50_ (12 h): Diallyl disulfide. 0.05 µg/L Methyl allyl disulfide: 0.07 µg/L	Song et al., 2023
Sophora flavencens	Solenopsis invicta	LC_50_ (7 days): S. flavencens ethanol extract: 1426.3 mg/L (minor workers) 2292.6 mg/L (major workers) Matrine: 46.8 mg/L (minor workers) 71.5 mg/L (major workers) Sophocarpine: 50.1 mg/L (minor workers) 85.9 mg/L (major workers)	Tian and Zhang, 2023
Cinnamomum loureirii; Cinnamomum cassia	Solenopsis invicta	LT_50_: C. loureirii bark oil 19 min ± 0.21 C. cassia bark oil 22 min ± 0.53 C. loureirii leaf oil 31 min ± 1.82 C. cassia leaf oil 33 min ± 0.99. trans-cinnamaldehyde 44 min ± 0.99.	Xing et al., 2023b
Asteraceae spp.	Solenopsis invicta	LC_50_ (24h): Seriphidium brevifolium: 2.18 µL/L Seriphidium kaschgaricum: 2.20 µL/L Seriphidium badghysum: 4.19 µL/L Seriphidium terrae-albae: 2.46 µL/L Seriphidium kurramense: 2.49 µL/L Seriphidium mongolorum: 2.34 µL/L Seriphidium balchanorum: 4.04 µL/L Seriphidium gracilescens: 2.22 µL/L Seriphidium schrenkianum: 2.36 µL/L Seriphidium tauricum: 2.38 µL/L 1,8-Cineole: 2.23 µL/L α-thujone: 2.29 µL/L β-thujone: 2.50 µL/L	Rizvi et al., 2025

median lethal dose (LD_50_), median lethal concentration (LC_50_), or lethal time (LT_50_). The values shown in the table are rounded.

The isothiocyanates emitted by *Brassicaceae* plants have also shown potent toxicity against major RIFA workers. Particularly, allyl isothiocyanate, 2-phenylethyl isothiocyanate, and 3-(methylthio) propyl isothiocyanate raised median lethal doses (LD_50_) of 7.99, 2.36, and 2.09 µg/ant, respectively. The median lethal concentrations (LC_50_) for allyl isothiocyanate and 3-(methylthio) propyl isothiocyanate were 32.5 and 57.6 µg/L, respectively (Du et al., 2020). Similarly, the LD_50_ of carvacrol (the primary constituent of *Lippia gracilis*; 51%), and pure *L. gracilis* essential oil were 6.79 and 6.11 µg/mg, respectively (Dantas et al., 2023). Considering the potency of this monoterpene, carvacrol derivatives were prepared to enhance its fungicidal properties. Among these derivatives, carvacryl benzoate stood out with an LD_50_ of 3.2 µg/mg. The *L. gracilis* essential oil, carvacrol, and carvacrol benzoate caused 100% mortality after 84, 60, and ~35 hours of exposure, respectively (Dantas et al., 2023). This approach pointed out the added value of natural compounds as basic structures for subsequent chemical optimization. Additionally, the crude extract from *Allium sativum* has also exhibited a strong fumigant effect against RIFA, causing 100% mortality in minor workers and 91.7% in major workers at 16 µg/mL after 12 hours post-treatment. The LC_50_ of diallyl disulfide and methyl allyl disulfide (the two main compounds of *A. sativum*) were 0.05 and 0.07 µg/L at 12 hours, respectively (Song et al., 2023). Furthermore, *Litsea cubeba* essential oil and its primary compound citral (27.76%) were evaluated against RIFA workers. After 24 hours of treatment, 5.33 µL/cm³ of *L. cubeba* essential oil caused 60% and 90% mortality in major and minor workers, respectively. Moreover, citral produced 76 and 96% mortality, respectively (Xiao et al., 2020). Other experimental evidence demonstrated the fumigant effect of essential oils from ten species of the *Seriphidium* genus, as well as 1,8-cineole, camphor, α-thujone, β-thujone, citral, and geraniol as bioactive compounds with LC_50_ ranging from 2.18 to 4.19 µL/L against RIFA (Rizvi et al., 2025). The essential oils from *S. brevifolium*, *S. kaschgaricum*, *S. terrae-albae*, and *S. kurramense* caused 100% mortality on minor and major workers of RIFA, whereas those from *S. mongolorum*, *S. schrenkianum*, *S. tauricum*, and *S. gracilescens* produced 90–97% mortality at 4 µL/L after 120 minutes. In addition, 1,8-cineole, (-)-α-thujone, (α+β)-thujone, and D (+)-camphor raised 100% mortality under the same assay conditions (Rizvi et al., 2025). Complementary evidence endorsed the effect of *S. brevifolium*, revealing (α+β)-thujone and 1,8-cineole with IC_50_ of 16.47, 19.11, and 30.04 µL/L, respectively, after 12 h post-treatment. Remarkably, 100% mortality was achieved after 24 hours of exposure at concentrations of 20, 28, and 80 µL/L (Xie et al., 2020). In contrast to single-compound assessment, the evaluation of a mixture of *trans*-cinnamaldehyde and cinnamyl acetate (50 µg/cm³ *trans*-cinnamaldehyde + 25 µg/cm³ cinnamyl acetate) isolated from *Cinnamon loureirii* led to 90% mortality on RIFA workers. The sole application of 100 µg/cm³ trans-cinnamaldehyde for 20 hours resulted in 100% mortality in RIFA workers. Nevertheless, the application of essential oil from the stem bark and leaves of *C. loureirii* resulted in 100% mortality within 1-hour post-treatment (Xing et al., 2023a).

Likewise, the oxygenated hydrocarbons 1-decanol and 1-octanol from *Magnolia grandiflora* were toxic to different RIFA and *S. richteri* castes, with LC_50_ values of 140.6 and 486.8 µg/g, respectively (Ali et al., 2022). Similarly, 100 µL of the vaporized essential oil from *Cupressus nootkatensis* caused 100% mortality in the same species after 48 hours (Addesso et al., 2017). In addition, the essential oils of *Matricaria chamomilla* cv. Egyptian, *M. chamomilla* cv. German CO2, and *M. chamomilla* cv. German also exerted toxic effects on RIFA, *S. richteri*, and their respective hybrids. The essential oils from the three cultivars of *M. chamomilla* exhibited LC_50_ from 93.6 to 202.5 µg/g against RIFA and *S. richteri* (Shah et al., 2023). α-Bisabolol was the principal component of the *M. chamomilla* essential oils and had an LC_50_ of 159.23 µg/g for RIFA workers. At the highest assayed dose (250 µg/g), mortality rates were 73% for *M. chamomilla* cv. Egyptian, 20% for *M. chamomilla* cv. German CO2, 40% for *M. chamomilla* cv. German, and 80% for α-bisabolol against related hybrids (Shah et al., 2023). In parallel, *Curcuma longa* leaf essential oil showed higher potency against RIFA and *S. richteri*, while *C. longa* rhizome essential oil was more effective against related hybrids (Ali et al., 2024). Also, the essential oil of *Baccharis microdonta* exhibited LC_50_ of 78.9 µg/g for RIFA, 97.5 µg/g for *S. richteri*, and 136.5 µg/g for derived hybrids after 24 hours (Ali et al., 2023). Furthermore, 2-undecanone from *Houttuynia cordata* exerted an LC_50_ of 44.59 µg/g through fumigant experiments against RIFA (Kurmanbayeva et al., 2023). On the other hand, concentrations of ~50 µg/mg of *Artemisia annua* essential oil caused 42% mortality in *Solenopsis saevissima* (Seixas et al., 2018). In addition, the insecticidal properties of eugenol and its derivatives have been demonstrated on RIFA workers (Wen et al., 2021; He et al., 2023). At 24 hours, the LC_50_ values obtained from fumigant experiments for eugenol, isoeugenol, methyl isoeugenol, eugenol acetate, methyl eugenol, and isoeugenyl acetate were 0.020, 0.003, 0.003, 0.004, 0.018, and 0.556 mg/cm², respectively. Interestingly, 100% mortality was observed by the application of methyl isoeugenol, isoeugenol, and eugenol acetate at 72 h (He et al., 2023). Interestingly, the *Piper auritum* essential oil, containing methyl eugenol as a major constituent, exerted a faster lethal effect on *S. geminata*. This essential oil raised 100% mortality within 3 h post-treatment at 18 µg/cm², whereas methyl eugenol reached the same effect within 15 min at 3 µg/cm². Both treatments showed comparable LD_50_ values (7.08 and 7.1 µg/cm², respectively), but methyl eugenol displayed a significant TL_50_ (5 min) compared with the essential oil (1 h 36 min), highlighting its efficiency through fumigation experiments (Flores-Hernández, 2024). To date, the essential oil from *Piper auritum* and methyl eugenol can be considered as one of the most promising biocide agents against *S. geminata* (Flores-Hernández, 2024). Another study focused on the evaluation of the *Sophora flavescens* ethanol extract, which revealed LC_50_ values of 1426.3 mg/L and 2292.6 mg/L for minor and major RIFA workers, whereas matrine (the major compound) showed higher toxicity, with LC_50_ values of 46.8 mg/L for minor workers and 71.5 mg/L for major workers (Tian and Zhang, 2023). Similarly, sophocarpine had LC_50_ values of 50.1 mg/L for minor workers and 85.9 mg/L for major workers (Tian and Zhang, 2023).

Studies using crushed or mashed plant material as a crude volatile-emitting matrix demonstrate that the slow and continuous release of endogenous volatiles can induce time-dependent insecticidal effects against RIFA. Fumigation assays using mashed leaves of *Murraya exotica* and *Michelia alba* rich in sesquiterpenes (β-caryophyllene, germacrene D) or monoterpenes (linalool) were associated with a gradual increase in mortality in minor and major workers, reaching up to 100% mortality after 9 days and 16 h exposure, respectively (Huang et al., 2016; Qin et al., 2018). This effect suggested that exposure to terpene volatiles directly emitted from plant materials may exert a slow biocide effect, which contrasts with the rapid knockdown raised by concentrated essential oils. On the other hand, evidence from rhizosphere-based assays supports this mechanism, since the incorporation of *Viburnum odoratissimum* and *Cinnamomum aromaticum* leaf debris into surface soils (0–10 cm and 5–10 cm depths, respectively) produced 68–100% mortality in both minor and major workers after contact periods of 12 days and 5 days, respectively (Huang et al., 2015; Zhang et al., 2017). These later studies demonstrate that the gradual volatilization from plant residues and enriched soils can generate a natural insecticidal pressure. Overall, plant-derived biopesticides display complementary strengths against *Solenopsis* spp., rather than a single optimal solution. Rapid knockdown is better achieved with phenolic volatile compounds such as methyl eugenol and selected essential oils, which induce complete mortality within minutes to hours at low doses. In contrast, compounds with extremely low LD_50_ values (e.g., azadirachtin, celangulin, and isothiocyanates) exhibit high intrinsic potency but may be limited by formulation and application constraints. Available evidence suggests that long-term control is more effective with crude plant matrices and soil-enriched residues, which promote gradual volatilization and sustained insecticidal pressure over time (Huang et al., 2015; Zhang et al., 2017). Current findings indicate that effective fire ant management should integrate fast-acting fumigants with slow-release systems to balance efficacy, persistence, and environmental compatibility (**Table 4**). For example, the previously discussed methyl eugenol from *Piper auritum* may provide rapid knockdown during early infestation stages, whereas plant residues from *Murraya exotica* may sustain prolonged volatile-mediated effects (Huang et al., 2016). Future studies should therefore evaluate integrated formulations that combine rapid fumigants with slow-release delivery systems, including bait-based matrices, nanoencapsulation, or soil-amendment approaches, under field conditions to optimize persistence, environmental safety, and colony-level suppression. Other plants with toxic effects on RIFA castes are shown in **Supplementary Table 3**.

**Table 4:** Representative fast-acting and prolonged-effect plant-derived biopesticides against *Solenopsis* spp.

Biopesticide strategy	Plant	Major bioactive constituent(s)	Exposure time	Best mortality outcome	Reference
Fast-acting	*Piper auritum*	methyl eugenol	15 min	100 %	(Flores-Hernández, 2024)
Fast-acting	*Seriphidium brevifolium*	1,8-cineole, (-)-α-thujone	2 h	90 - 97 %	(Rizvi et al., 2025)
Fast-acting	*Citrus paradis*i	octanal	6 h	100 %	(Zhang et al., 2021)
Fast-acting	*Citrus paradisi*	α-pinene	8 h	100 %	(Zhang et al., 2021)
Fast-acting	*Allium sativum*	diallyl disulfide	12 h	100 %	(Song et al., 2023)
Fast-acting	*Cinnamon loureirii*	trans-cinnamaldehyde	20 h	100 %	(Xing et al., 2023a)
Fast-acting	*Litsea cubeba*	citral	24 h	76 - 96 %	(Xiao et al., 2020)
Prolonged-effect	*Michelia alba*	linalool	16–64 h	100 %	(Qin et al., 2018)
Prolonged-effect	*Azadirachta indica*	azadirachtin	72 h	91 %	(Liang et al., 2023)
Prolonged-effect	*Celastrus angulatus*	celangulin	72 h	97 %	(Liang et al., 2023)
Prolonged-effect		methyl isoeugenol, isoeugenol, and eugenol acetate	3 days	100 %	(He et al., 2023)
Prolonged-effect	*Cinnamomum aromaticum*	Cinnamic aldehyde	5 days	64 - 100 %	(Huang et al., 2015)
Prolonged-effect	*Murraya exotica*	β-caryophyllene	9 days	83 - 100 %	(Huang et al., 2016)
Prolonged-effect	*Viburnum odoratissimum*	Methyl salicylate	12 days	69 - 100 %	(Zhang et al., 2017)

### 4.2 Repellency

Repellency constitutes a more accepted agroecological alternative than the biocidal control of *Solenopsis* spp. This strategy is usually based on disrupting chemosensory-mediated foraging and trail-following behaviors at sublethal concentrations (Ali et al., 2024). Natural repellency is particularly suitable for attenuating side effects in crops through the conservation of beneficial arthropods and decreasing resistance to synthetic compounds. Plant-derived repellents that interfere with olfactory signal perception at low doses are more appropriate for spatial exclusion than for direct colony elimination (Du et al., 2021b). The repellency of natural products against fire ants can be evaluated through specific behaviors, such as ant-digging behavioral changes in RIFA, which is related to nest construction and foraging (**Supplementary Table 4**).

#### 4.2.1 Digging and spatial repellency

It has been demonstrated that nootka essential oil (*Cupressus nootkatensis*) significantly reduced digging behavior in hybrid fire ants, showing no sand removal from treatments of 1 to 100 µL essential oil. Experiments measuring digging behavior using 10 µl of nootka essential oil over 1–3 months resulted in 5-28% sand removal compared with untreated control groups (Addesso et al., 2017). The persistence of reduced digging activity over extended exposure periods suggests a chemical stability degree as a practical parameter for long-term field applications. Similarly, the *Magnolia grandiflora* seed essential oil and its major compounds 1-decanol and 1-octanol significantly decreased the digging behavior of hybrid fire ants at dosages of 4.9-156 µg/g, 0.31-156 µg/g, and 4.9-156 µg/g, respectively (Ali et al., 2022). Likewise, *Curcuma longa* leaf/rhizome essential oil induced ant-digging suppression at 19.5/19.5, 9.8/39, and 4.9/4.9 µg/g against RIFA, *S. ritcheri*, and derived hybrids, respectively. Ar-turmerone is one of the most abundant volatiles accumulated in *C. longa* and possesses strong repellency (19.5 µg/g) against RIFA workers (Ali et al., 2024). Concentrations of 4.9, 4.9, and 39 µg/g *Baccharis microdonta* essential oil were effective against RIFA, *S. ritcheri*, and derived hybrids, respectively (Ali et al., 2023). Common phenols such as carvacrol and thymol had a similar effect on RIFA, *S. ritcheri*, and their derived hybrids at concentrations ranging from 0.98 to 31.25 µg/g (Ali et al., 2023). Remarkably, the carboxylated derivatives of these compounds did not show comparable activity to that of carvacrol and thymol (Paudel et al., 2023). Consistently, the essential oil of *Matricaria chamomilla* also showed a significant repellency in digging experiments. The repellency of *M. chamomilla* cv. Egyptian, *M. chamomilla* German CO2 essential oils, and α-bisabolol against RIFA, *S. ritcheri*, and derived hybrids ranged from 3.9 to 31.25 µg/g. On the other hand, the repellence range for *M. chamomilla* cv. German essential oil was 15.6-125 µg/g against RIFA and 15.6–3.9 µg/g against *S. ritcheri* and derived hybrids (Shah et al., 2023). The high variability observed among *M. chamomilla* cultivars suggests the need for chemical standardization when comparing repellent performance across plant sources. Extending this approach to soil-based systems, the repellency of *Viburnum odoratissimum* at different soil depths inside the rhizosphere was evaluated (Zhang et al., 2017). No digging behavior was observed for both minor and major ants after 10 days of treatment with soil samples containing 4.88 mg/kg of methyl salicylate at a depth of 0–5 cm (Zhang et al., 2017). The soil impregnation of *Cinnamomum aromaticum* essential oil at 10–15 cm resulted in 73.7-79.6% minor and major worker repellency after 24 h post-treatment. The major compounds of this essential oil were eugenol and cinnamic aldehyde (Huang et al., 2015). The latter research works suggest that eugenol- and cinnamaldehyde-rich matrices can be used as lead compounds for soil-based repellency. *H. thyrsiforme* exhibited a moderate repellency at 100.0 mg/kg on RIFA workers, while *H. elatum* was effective at 1.0 and 10.0 mg/kg on the same caste (Sakhanokho et al., 2013). The essential oils from *Artemisia argyi*, *Aquilaria sinensis*, *Cymbopogon citratus*, *Mentha canadensis*, *Pogostemon cablin*, *Santalum album*, *Thymus mongolicus*, and *Zingiber officinale* show strong repellency against RIFA workers (~1% sand removal in 24 h) (Fan et al., 2024). Major compounds, including dipentene, α-terpineol, and linalool, exhibited significant repellency (~ 1% sand removal in 24 h) (Fan et al., 2024). Because digging relies on coordinated sensory input and motor output, its suppression provides an indirect indicator of physiological stress affecting neural and muscular function. In addition, the essential oils from *A. argyi* and *S. album* significantly reduced the ant climbing rate in the treated Y-type board arm to less than 20% within 60 seconds (Fan et al., 2024).

#### 4.2.2 Behavioral and olfactory disruption

Beyond digging suppression, ant repellency degree can also be evaluated by aggregation, walking, grooming, climbing, fighting, grasping, defending, crawling, drinking, foraging, and particle-covering behaviors. For instance, *Blumea balsamifera* and *Illicium verum* essential oils influenced RIFA workers’ aggregation at concentrations of 0.001-0.01 µL/cm³ (Fu et al., 2023). *Acorus tatarinowii* essential oil completely inhibited aggregation after 4 hours at 0.005 and 0.01 µL/cm³ (Fu et al., 2023). After 36 hours, aggression levels were reduced to 20% with *A. tatarinowii*, *I. verum*, and *B. balsamifera* at a concentration of 0.001 µL/cm³. *I. verum* significantly impaired grasping ability on RIFA workers at 0.001 µL/cm³ after 24 hours (Fu et al., 2023). Moreover, volatile emissions from crushed *A. sativum* bulbs significantly declined grasping and walking behavior from 2 to 12 hours of exposure, reaching negligible activity at ~16 µg/mL in minor and major workers (Song et al., 2023). Similarly, *C. loureirii* leaf essential oil reduced grasping and knocked down assayed ants at 320 µg/cm³ within 10 minutes post-treatment, whereas *C. loureirii* stem bark essential oil produced the same effect at 50 minutes post-treatment (Xing et al., 2023a). The highly effective knockdown times (KT_50_) for 500 µg/mL azadirachtin and celangulin were 5.96 and 7.06 min, respectively, in RIFA workers (Liang et al., 2023). Furthermore, RIFA workers’ foraging was fully suppressed on tape squares treated with 2 µL/cm² essential balm (menthol-48%, methyl salicylate-27%, eucalyptol-10%, D-(+)-camphor-7%, eugenol-4%, phenylethyl alcohol-2%, dipropylene glycol-1%) within 24 hours (Wen et al., 2016). Between 12 and 24 hours, ants displayed intense particle transport after exposure to sausage on patches impregnated with 1 and 2 µL/cm² essential balm (Wen et al., 2016). Foraging behavior was additionally evaluated through ham sausage baits. After 1 day of treatment at 50 mg/mL, the crude extract of *Kaempferia galanga* demonstrated the most effective non-selective antifeedant activity against RIFA, resulting in 52.67% decrease in foraging behavior. The crude extract of *Rosmarinus officinalis* exhibited the best selective antifeedant activity at 50 mg/mL, with a selective rate of 69.33% (Guo et al., 2025). Consistently, RIFA decreased mealworms foraging from 55 to 95% with 2.5 µL/ml essential oils from *Seriphidium brevifolium*, *S. kaschgaricum*, *S. terrae-albae*, *S. kurramense*, *S. mongolorum*, *S. gracilescens*, *S. schrenkianum*, and *S. tauricum* and their major compounds 1,8-cineole, D (+)-camphor, (α+β)-thujone, (-)-α-thujone, citral, and geraniol (Rizvi et al., 2025). RIFA significantly reduced defensive behavior and avoided surfaces impregnated with all essential oils assayed (Wen et al., 2016). Likewise, after 24 hours of exposure, a dose of 5.33 µL/mL of *L. cubeba* essential oil reduced the walking rates of major and minor ants to 32% and 16%, respectively. Similarly, 5.33 µL/mL citral (the main compound of *L. cubeba* oil) decreased walking rates up to 16% in major ants and 0% in minor ants for 24 hours. Mixtures of *L. cubeba* oil and citral reduced the climbing rate to 16% in major ants and 0% in minor ants after 24 hours post-treatment (Xiao et al., 2020). α-Pinene was the most toxic compound isolated from *Citrus paradisi*, causing walking changes in 80% of the RIFA workers assayed. Octanal, α-pinene, and β-phellandrene had a stronger impact on gripping ability, reducing it from 13 to 25% among RIFA workers (Zhang et al., 2021). At the behavioral level, RIFA workers exhibited increased self-cleaning and reduced allogrooming and aggregation when exposed to *L. gracilis* essential oil at 3.42 µg/mg (Dantas et al., 2023). RIFA workers treated with carvacrol (2.76 µg/mg) and carvacryl benzoate (2.21 µg/mg) led to a 27 and 38% increase in walking distance and speed compared with non-treated RIFA workers (Dantas et al., 2023). *C. loureirii* essential oil also induced a higher leg-antennal grooming frequency (bark: 88, leaf: 101 times/2 min) compared with *Cinnamomum cassia* essential oil (bark: 72, leaf: 50 times/2 min). Also, double-antennal grooming was also induced by *C. loureirii* essential oil (bark and leaf: 56 times/2 min) and *C. cassia* bark essential oil (55 times/2 min) compared with *C. cassia* leaf essential oil (41 times/2 min) (Xing et al., 2023b). Moreover, the application of *C. loureirii* bark essential oil (20%) increased fighting behavior compared to that caused by *C. cassia* bark oil (16.67%). For *trans*-cinnamaldehyde, leg-antennal grooming frequency was 77 times/2 min, double-antennal grooming 76 times/2 min, and a maximum fighting rate of 58% within 20 minutes (Xing et al., 2023b).

**Figure 2:**
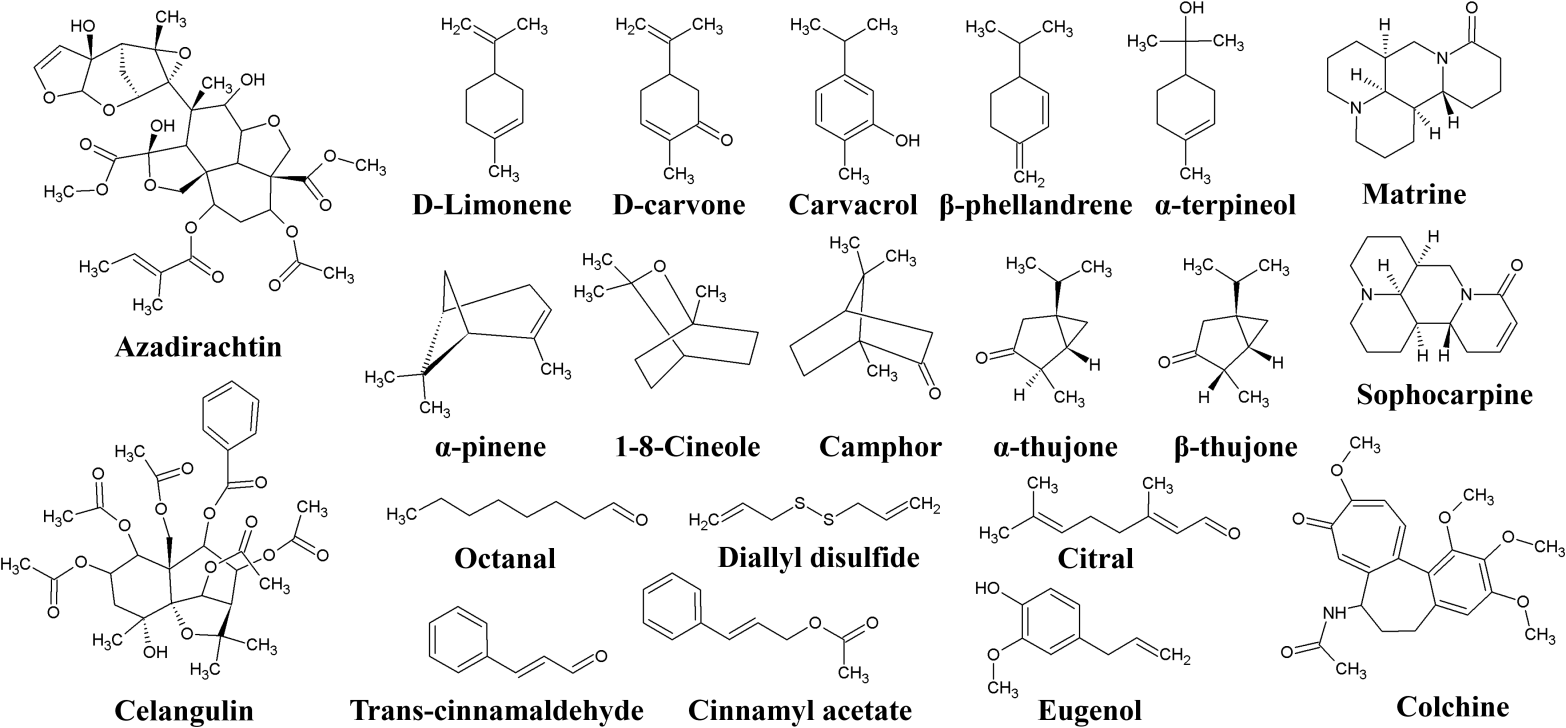
Natural products with potential application in the control of *Solenopsis* spp. obtained from PubChem SMILES codes and visualized in ACD/ChemSketch (Advanced Chemistry Development, Inc (2023)).

Complementary evidence from olfactometry assays showed that different concentrations of bioactive essential oils produce repellent effects on RIFA, as demonstrated by Y-type olfactometry. The essential oil from *Osmanthus fragans* showed 72% repellent activity on minor RIFA workers and 64% on major workers at 200 μg/ml, while male ants displayed a 62.5% repellent rate at 8 μg/ml. Similarly, the essential oil from *Eugenia caryophyllata* repelled RIFA workers up to 66% at 40 μg/mL (Li et al., 2022). The essential oil from *Ligustrum compactum* showed a repellent rate of 69% at 1000 μg/mL on minor RIFA workers and 63% at 200 μg/mL on major workers. On the other hand, the essential oil of *Jasminum sambac* produced a 63% repellent effect on male ants at 1000 μg/mL, while the *Allium polyrhizum* essential oil repelled small worker ants by 65% at the same concentration (Li et al., 2022). Interestingly, *Salvia miltiorrhiza* and *Perilla frutescens* essential oils repelled 65% small and large worker ants at 1000 μg/ml, whereas *P. frutescens* essential oil produced ~70% repellency in large workers and virgin queens at 1000 μg/mL. *Pogostemon cablin* essential oil repelled 64% small workers, as well as ~70% large workers and virgin queens at 2 μg/ml (Wang et al., 2024). Moreover, *Schizonepeta tenusfolia* essential oil exhibited 85% repellency against small workers and 68% against large workers. Also, *Mentha canadensis* essential oil had repellent effects on small workers (77%), large workers (63%), male ants (68%), and virgin queens (64%). Among specific compounds dissolved in the essential oil, menthone, menthol, and caryophyllene oxide produced a repellency of 74, 89, and 74%, respectively, in small workers, while 3-methylcyclopentanol showed 70% repellency in large workers (Wang et al., 2024). Collectively, methyl salicylate, eugenol/cinnamaldehyde-rich formulations, and phenolic monoterpenes such as carvacrol emerge as the most promising candidates for scalable repellency-based management of *Solenopsis* spp., combining low-dose efficacy, chemical stability, and broad behavioral disruption. The consistent suppression of digging, foraging, and locomotor activities supports a sublethal mode of action involving chemosensory interference and downstream neuromuscular regulation, impairing colony performance and spatial occupation. Although these properties can be exploited for preventive and area-based applications, further field validation should be considered. **Figure 2** shows some natural products visualized as promising plant-derived biopesticides discussed in previous sections. Also, **Supplementary Tables S1** and **S2** summarize the major bioactive compounds from biocidal plants against *Solenopsis* spp. including their relative abundance and chemical structures.

## 5 Mechanism of action of anti-fire ant molecules derived from plants

Overall, the mechanisms of action of insecticides can be grouped into specific targets, including the nervous and muscular systems, energy production pathways, and physiological processes related to growth and development (Rezende-Teixeira et al., 2022). In this section, we address two of the most studied insecticidal targets for *Solenopsis* spp.

### 5.1 Acetylcholinesterase

AChE is the most common target for organophosphate and carbamate insecticides, which act as enzyme inhibitors (Rezende-Teixeira et al., 2022). AChE inhibition leads to the accumulation of acetylcholine in the synaptic cleft, resulting in excessive stimulation of the nervous system and the insect’s death (Dos Santos et al., 2019a). Even in humans, AChE inhibition by organophosphates is highly hazardous because they can block the enzyme’s catalytic sites, leading to an acute cholinergic crisis (Gerlits et al., 2019). Under normal conditions, AChE (EC 3.1.1.7) is located in the synaptic cleft of cholinergic neurons and is involved in finishing nerve impulses by rapidly converting acetylcholine into acetate and choline in the nervous systems of most animals, including insects (Dvir et al., 2010). In insects, the locus ace1 and locus ace2 encode AChE1 and AChE2 proteins, respectively. Usually, AChE1 participates in the transmission of cholinergic signals, while AChE2 is involved in non-cholinergic functions (Kim and Lee, 2013). For some Hymenoptera species, such as *A. mellifera*, *Bombus ignitus*, and *Atta sexdens* (leaf-cutting ant), AChE2 is the main catalytic enzyme (Kim and Lee, 2013; Dos Santos et al., 2019a). The crystal structure of *Torpedo californica* acetylcholinesterase (TcAChE) provides insights into the three-dimensional structure of this enzyme (Dvir et al., 2010). AChE is a serine hydrolase belonging to the α/β hydrolase family. The active site is located near the bottom of a deep and narrow gorge and contains S200, E327, and H440 as the catalytic triad (Dvir et al., 2010). The gorge is lined with aromatic residues, such as W84 and F330, forming the catalytic anionic site (CAS), and with Y70, Y121, and W279, which constitute the peripheral anionic site (PAS) (Silman and Sussman, 2008). *Tc*AChE possesses a dipole moment putatively involved in substrate binding interaction (Dvir et al., 2010; Silman and Sussman, 2008). Interestingly, the crystallized structure of acetylcholinesterase of *D. melanogaster* (*Dm*AChE) shows a low similarity with that of *Tc*AChE. For the invertebrate *Dm*AChE, the active site is composed of the residues S238, E367, and H480 (Harel et al., 2000). It has been reported that the *Dm*AChE protein sequence shares 36% homology with *Tc*AChE, and ~54% against human (*Homo sapiens*) acetylcholinesterase (*Hs*AChE) and mouse acetylcholinesterase (*m*AChE). Although the low sequence homology among AChE orthologs, their three-dimensional structures are folded similarly (Harel et al., 2000). Marked structural differences between *Dm*AChE and *Tc*AChE are observed in the loop regions, the C-terminal domain, and the active-site gorge, which is approximately 50% smaller in *Dm*AChE than in *Tc*AChE. These features define distinct steric and physicochemical environments that are highly relevant for the rational design of selective inhibitors targeting insect acetylcholinesterase. For this purpose, candidate residues are Y71, Y73, E80, and D375, which are located near the gorge opening region in the active site of *Dm*AChE (Harel et al., 2000). Other candidates are W83, W472, and D482 residues that delimit an opening of ~5 Å diameter pore at the bottom of the gorge active site in *Dm*AChE. In *Hs*AChE, the residues W86, W472, and Y449 interact with each other through hydrogen bonds, and the conformational change would be required to form the pore (Nachon et al., 2020). Furthermore, an insect-specific cysteine residue located at the opening of the AChE active site, C290 in *Dm*AChE, is considered a good insecticidal target, as a phenylalanine in *Hs*AChE replaces that residue. Nonetheless, in a tridimensional projection, this residue is hidden by other residues and is inaccessible for interaction. Remarkably, C289 is conserved in at least 22 insect species, including various mosquito species, and is considered a promising target due to its location near the active-site entrance. On the other hand, *Hs*AChE replaces C289 with F295 (Pang et al., 2012). To date, no report has been published on the crystallized structure of AChE from *S. invicta*. Given this lack of scientific knowledge, further investigation into gene isolation and functional genomics is required to characterize the structure and biochemical properties of AChE from imported fire ants.

**Figure 3:**
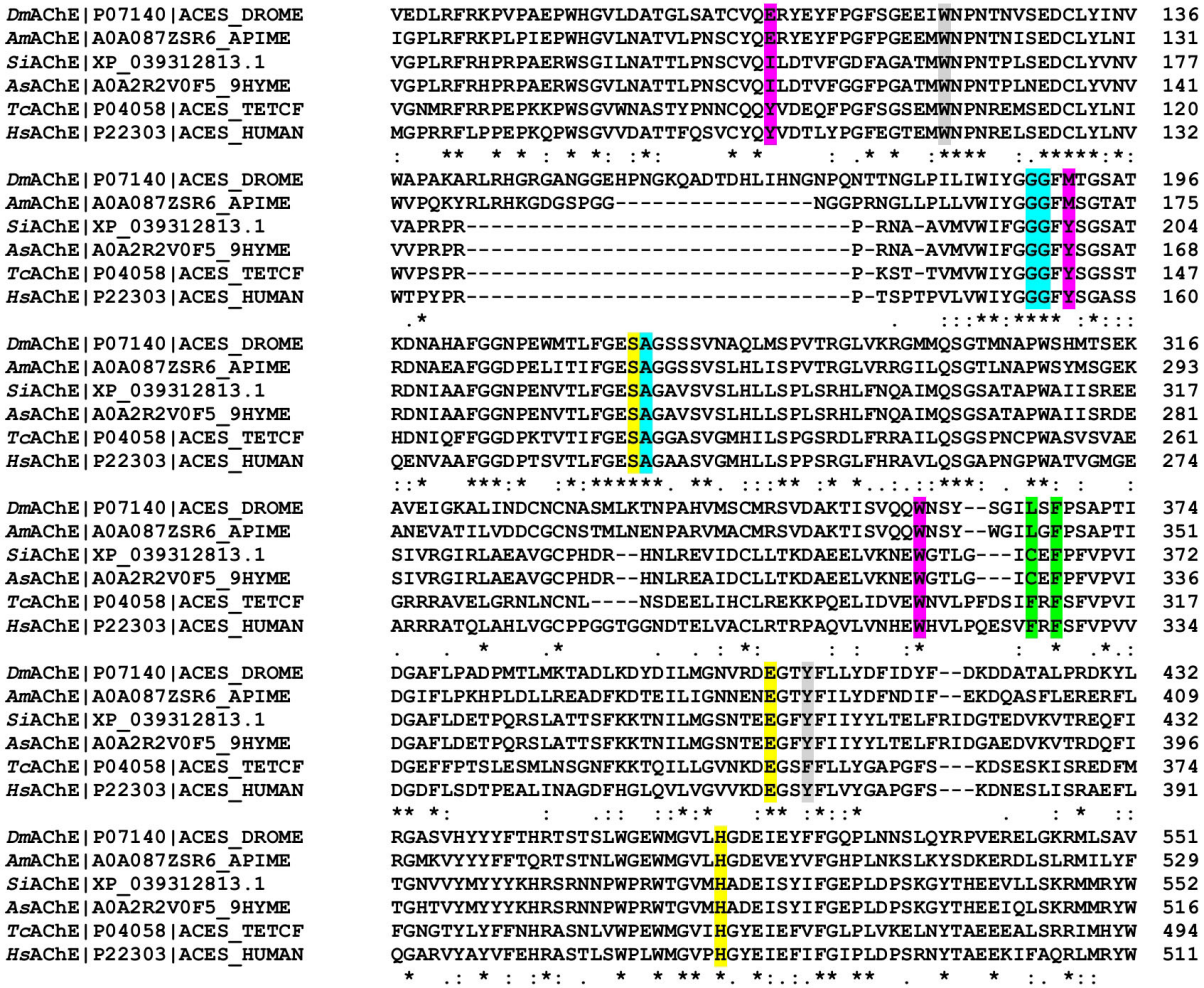
Multiple sequence alignment of acetylcholinesterase from *Dm*AChE (P07140), *Am*AChE (A0A087ZSR6), *Si*AChE (XP_039312813.1), *As*AChE (A0A2R2V0F5), *Tc*AChE (P04058), and *Hs*AChE (P22303). The homology analysis was generated using Clustal Omega (version 1.2.4). Major binding residues are highlighted in a color code. Active site residues are highlighted in yellow; oxyanion residues are highlighted in cyan; CAS residues are highlighted in grey; PAS residues are highlighted in magenta; and entrance residues are highlighted in green. Symbols at the bottom indicate complete homology (*), moderate homology (.), and similar homology (.) based on comparable amino acid properties.

Multiple sequence alignment of AChE using the predicted amino acid sequences of *S. invicta* (*Si*AChE), *D. melanogaster* (*Dm*AChE), *A. mellifera* (*Am*AChE), *A. sexdens rubropilosa* (*As*AChE), *T. californica* (*Tc*AChE), and *H. sapiens* (*Hs*AChE), revealed conserved and divergent regions across insect and vertebrate species (**Figure 3**). Multiple sequence alignments are generally useful for identifying differences among pest species and for generating targeted insecticides that minimize side effects to beneficial insects (e.g., *A. mellifera*), humans, and other vertebrates. *Tc*AChE has been identified as a classical model for drug discovery and for identifying toxic compounds with potential impact on human neurobiology and toxicology. A homology analysis of the active site, oxyanion hole, CAS, PAS, acyl pocket, and active-site entrance residues revealed clear differences among available AChE coding sequences. It was found that *As*AChE (95.07%), *Hs*AChE (43.40%), *Tc*AChE (42.86%), *Am*AChE (41.43%), and *Dm*AChE (38.67%) present different homology degrees when compared with *Dm*AChE. Interestingly, the active site, oxyanion hole, and entrance residues (including phenylalanine) are apparently well-conserved in the six AChEs analyzed (**Figure 3**). This finding indicates that the catalytic triad (S, D, and H) residues remain conserved across phylogenetic divergence. For the CAS site, there is high homology between tryptophan and tyrosine residues across the six AChEs, except for *Tc*AChE, which contains a phenylalanine instead of a tyrosine. In the case of PAS residues, tryptophan and tyrosine residues also showed high homology across all six species, except for *Dm*AChE and *Am*AChE, which contained methionine instead of tyrosine. For the third PAS position, *Dm*AChE and *Am*AChE present a glutamic acid residue, where the ant AChEs (*Si*AChE and *As*AChE) contain isoleucine, whereas *Tm*AChE and *Hs*AChE display tyrosine. This interspecies variability, particularly among ants, bees, and humans, suggests that the PAS position may contribute to differential ligand recognition and should be considered in the design of insect-selective AChE inhibitors (**Table 5**). Pang et al. (2012) suggested residues visualized as possible targets located at the active-site entrance. These residues correspond to L328 in *Dm*AChE and *Am*AChE, a C328 in ant species (*Si*AChE and *As*AChE), and an F295 in *Hs*AChE. Another insecticidal target described by Harel et al. (2000) corresponds to Y375 in *As*AChE, which has a similar location and function to that of G342 in *Hs*AChE and D375 in *Dm*AChE, located at the active-site entrance.

**Table 5:** Main residues of the AChE tridimensional structures.

Site	TcAChE	DmAChE	AsAChE	HsAChE
3D-Structure code	PDB: 2ACE	PDB: 6XYS	AlphaFoldDB: A0A2R2V0F5	PDB: 605R
Active site (AS)	S200, E327, H440	S238, E367, H480	S240, E367, H483	S203, E334, H447
Oxyanion hole	G118, G119, A201	G150, G151, A239	G160, G161, A242	G120, G121, A204
CAS	W84, F330	W83, Y370	W126, Y370	W86, Y337
PAS	Y70, Y121, W279	E69, M153, W321	I112, Y163, W322	Y72, Y124, W286
Acyl pocket	F288	L328	C328	F295
Entrance AS	F290	F330	F330	F297
Bottom AS	W84, W432, Y442	W83, W472, D482	W126, W475, D485	W86, W439, Y449

**Figure 4:**
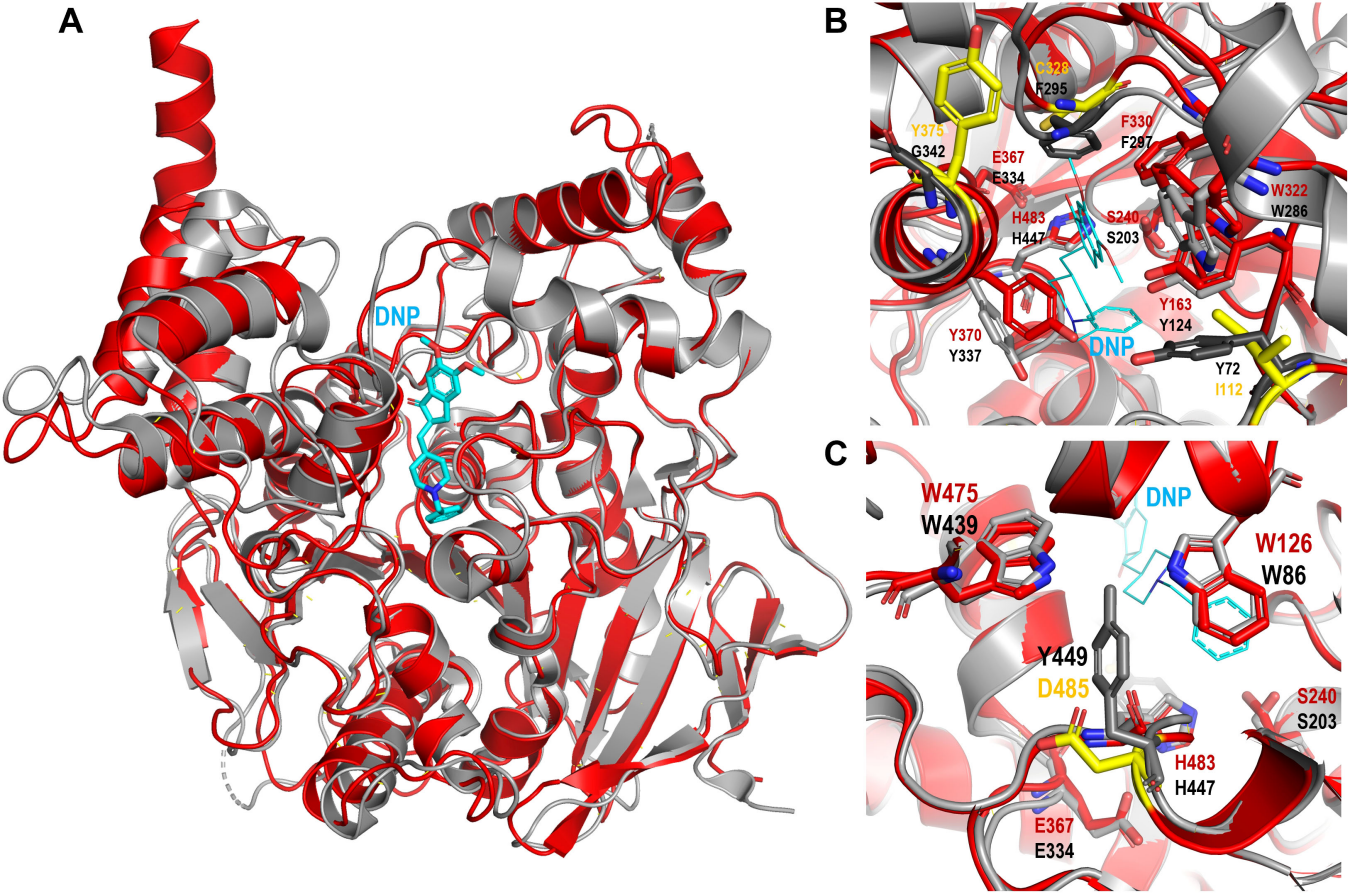
**(A)** Three-dimensional backbone alignment of the *As*AChE structure (red) (AlphaFold PDB: A0A2R2V0F5) over the human counterpart (PDB 4EY7) (grey) in complex with donepezil (DNP) inhibitor. **(B)** An active site close-up upper view showing the catalytic triad at the gorge (*As*AChE: S240, E367, and H483). **(C)** An active site close-up bottom view, showing residues forming the channel (*As*AChE: W126, W475, and D485) toward the active-site gorge. In **(B, C)** the main insecticidal target residues for *As*AChE are highlighted in yellow, and for *Hs*AChE in black.

**Figure 4** illustrates the structural superposition of acetylcholinesterase from *A. sexdens* and that of humans. The predicted structure of *As*AChE was selected due to its high sequence and structural homology with *Si*AChE. As shown in **Figure 4A**, the overall fold of both enzymes is highly conserved upon superimposition. Notably, residues C328, I112, and Y375 in *As*AChE were located at the entrance of the active-site gorge, suggesting their potential relevance for the design of insect-selective inhibitors (**Figure 4B**). In addition, D485 at the base of the *As*AChE active site forms a wider opening pore compared to the corresponding Y499 in *Hs*AChE, which is stabilized by W86, W472, and G82 (Nachon et al., 2020) (**Figure 4C**). **Supplementary Table 5** summarizes experimental evidence supporting the AChE inhibitory activity of plant-derived compounds that have previously been reported to exhibit insecticidal or repellent effects against *Solenopsis* spp.

### 5.2 Glutathione-S-transferase

The glutathione-S-transferases (GSTs) (EC 2.5.1.18) are involved in the resistance mechanism to a variety of well-known insecticides such as dichloro-diphenyl-trichloroethane (DDT) and organophosphates (Enayati et al., 2005). GSTs are critical enzymes involved in detoxification and catalyze the conjugation of glutathione (GSH) to a variety of endogenous and exogenous electrophilic compounds (Atkinson and Babbitt, 2009). Particularly, GSTs transform xenobiotics and endogenous toxins into more readily assimilable compounds, facilitating their solubilization and further elimination (Atkinson and Babbitt, 2009). In insects, GSTs play a critical role in the defense against insecticides, plant allelochemicals, and oxidative stress (Friedman, 2011). In humans, GSTs play a similar role in protecting cells from toxins, carcinogens, drugs, and oxidative stress products (Kado et al., 2012). GSTs are divided into cytosolic, mitochondrial, and microsomal families of proteins. Cytosolic GSTs are also divided into several classes, such as alpha, beta, delta, epsilon, mu, sigma, omega, etc (Oakley, 2011). Among these protein groups, delta and epsilon are restricted to insects and play a crucial role in insecticide resistance. GSTs grouped in the same family share approximately 40% sequence identity, while those from different classes show about 25% identity (Oakley, 2011). Indeed, the purified structures of GSTs from RIFA correspond to the sigma class, having 44% identity with that of the same class from *Anopheles gambiae*. Using naming conventions, the suggested code for the GST isolated from RIFA is *Si*GSTS1 (*S. invicta*, glutathione S-transferase, sigma class, enzyme 1) (Valles et al., 2003). The expression levels of *Si*GSTS1 differ between the monogyne and polygyne social forms of *S. invicta*, as well as during different developmental life stages. Early instars (13.1-fold) and workers (9.6-fold) showed the highest gene expression among all polygyne developmental stages. A comparable expression pattern was observed in the monogyne social form, where the queen had the lowest gene expression, followed by pupae (1.2-fold), late larvae (5.8-fold), early larvae (9.4-fold), and workers (10.1-fold). Because of the prolonged exposure to environmental toxins, workers often exhibit the highest levels of expression, which are directly proportional to their detoxification capacity (Valles et al., 2006).

Structurally, insect GSTs are typically cytosolic and dimeric proteins, with each monomer consisting of a conserved N-terminal domain that binds the sulfhydryl form of glutathione (GSH; G-site) and a more variable C-terminal domain involved in binding diverse substrates (Enayati et al., 2005). Residues from the C-terminal domain interact with electrophilic substrates in the hydrophobic H-site (Enayati et al., 2005). Also, each monomer contained a conserved thioredoxin domain, which includes the GSH binding site. The *Si*GSTS1 belongs to the Y-GSTs subgroup, where the Y8 hydroxyl group participates as a hydrogen bond donor to the GSH sulfur group. Overall, Y-GST subgroups contain the alpha, mu, pi, and sigma classes, while the S/C-GST subgroup is composed of phi, tau, theta, zeta, omega, and beta classes (Atkinson and Babbitt, 2009). Other important residues in the thioredoxin domain are R99 (involved in catalysis among the sigma GST class) and Q63-S64 residues (which stabilize the GSH interaction) (Valles et al., 2003). Human cytosolic GSTs (*Hs*GST) form homodimers or heterodimers. The sigma-class *Hs*GST, identified as hematopoietic prostaglandin D synthase (H-PGDS), has a crystallized structure with Ca²^+^ and Mg²^+^ binding at the dimer interface, which contributes to the enzyme’s activation. The H-PGDS has three distinct active sites, including pocket 1, formed by M99, F102, and W104, pocket 2, formed by R14, D96, Y152, and I155, and finally, pocket 3, formed by K112 and K198. Among these residues, W104 is located at the top of the active site pocket and plays an important role in maintaining the structural integrity of GST and in the catalytic activity of PGDS. Also, the K112 and K198 residues are involved in prostaglandin H2 recognition (Kado et al., 2012). For *A. mellifera* GST delta structure (*Am*GSTD1), the G-site is composed of S34, P35, P36, H63, Q74, H75, I77, P78, E89, S90, and Y130. Among these residues, S34 is a key amino acid involved in the GSH binding mechanism. Finally, the H-site comprises the residues F142, Y138, F229, M134, and Y130 (Moural et al., 2024).

**Figure 5:**
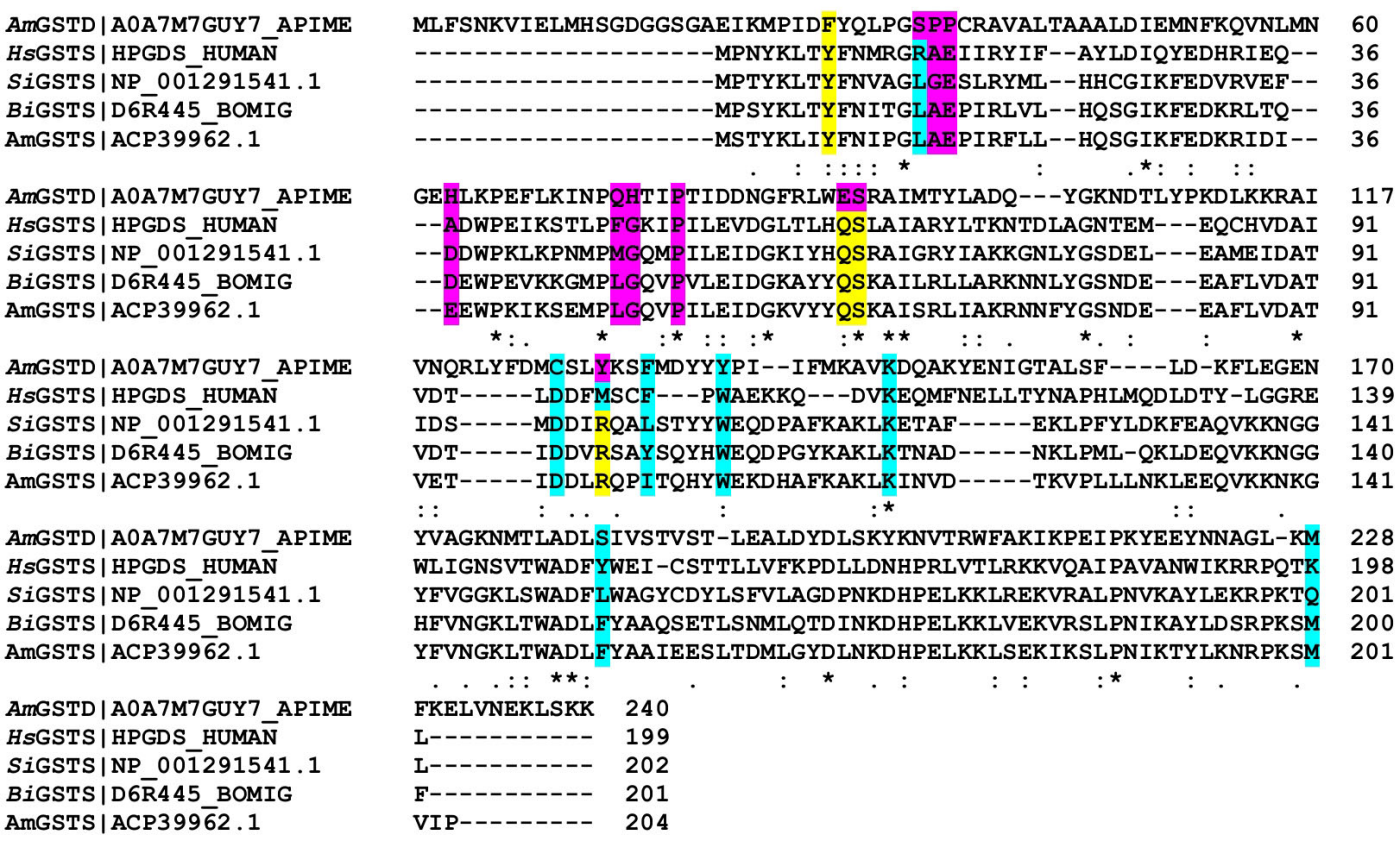
Multiple sequence alignment of GSTs from *Am*GSTD (A0A7M7GUY7), *Hs*GSTS (HPGDS), *Si*GSTS (NP_001291541.1), *Bi*GSTS (D6R445), and *Am*GSTS (ACP39962.1). The homology analysis was generated using Clustal Omega (version 1.2.4). Active site residues are color-coded as follows: yellow for S. invicta, cyan for humans, and magenta for the *A. mellifera* delta structure. Symbols at the bottom indicate complete homology (*), moderate homology (.), and similar homology (.) based on comparable amino acid properties.

**Figure 6:**
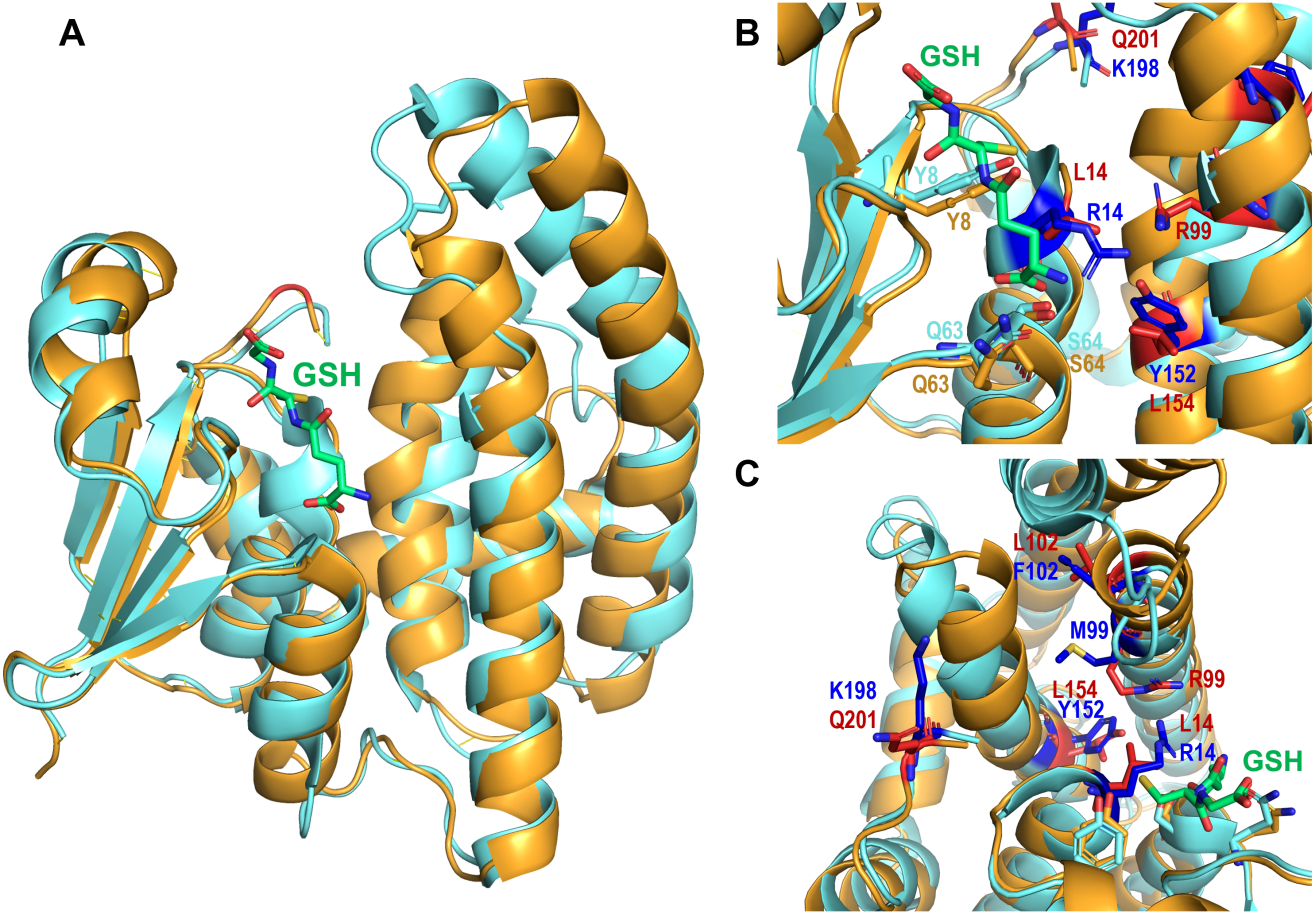
**(A)** Overlapping of the insect GST monomeric enzyme (orange) from *Solenopsis invicta* (AlphaFold PDB: Q3HV18) over the human counterpart (PDB 3VI7) structure (aquamarine). **(B)** An active site close-up is shown where the catalytic residues are indicated. **(C)** An active site upper view is shown to visualize residues that differ between the two organisms. In **(B, C)** the main proposal insecticidal target residues are highlighted for the *Si*GST (red) and *Hs*GST (blue).

**Figure 7:**
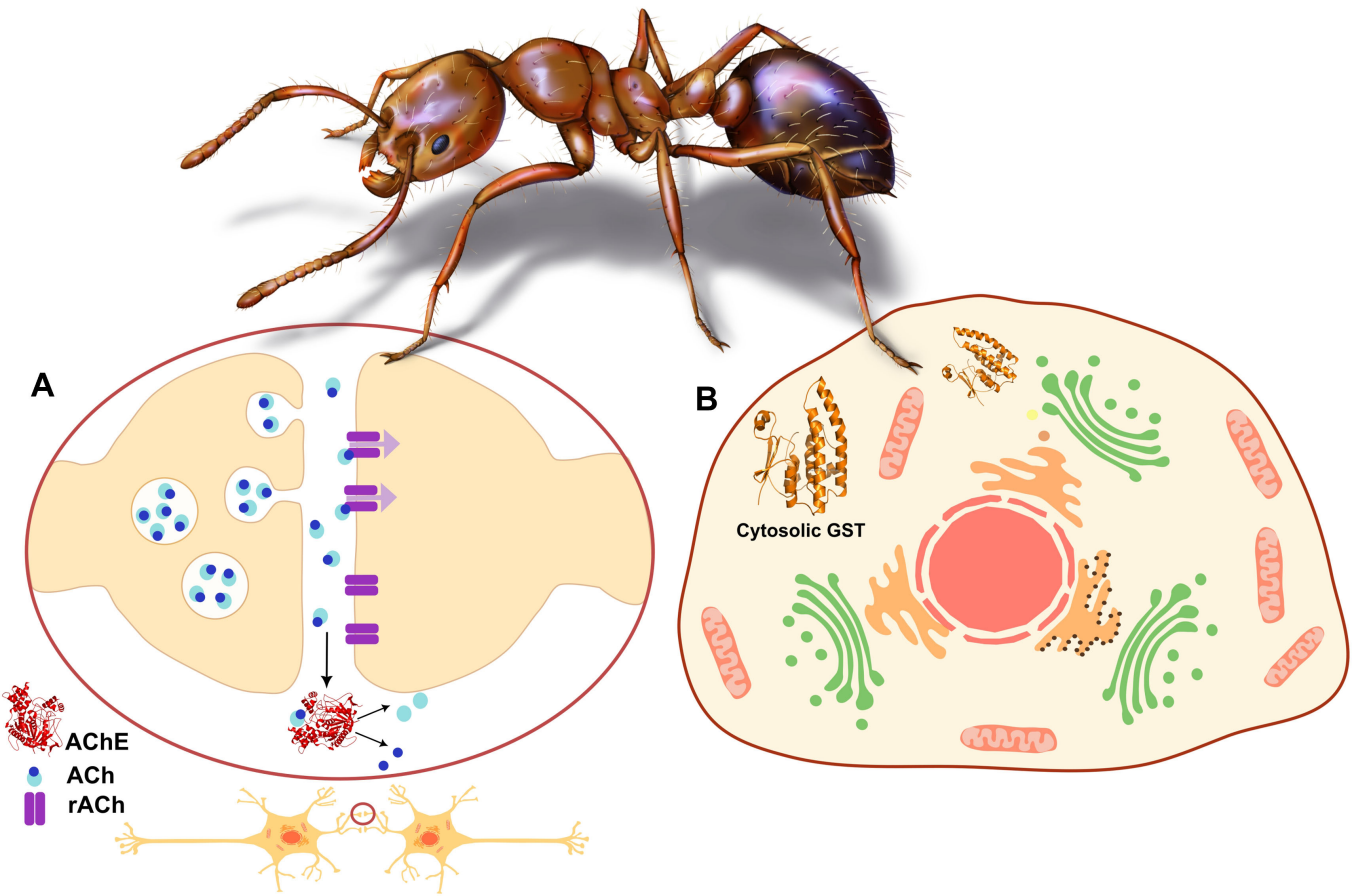
Mechanisms of action and AChE localization in *Solenopsis invicta*. **(A)** Localization of AChE in the synaptic cleft during synapse, where the primary function is to break down the acetylcholine (ACh). ACh binds to postsynaptic receptors (rACh). **(B)** The cytosolic GST enzyme is predominantly expressed in midgut epithelial cells and fat body cells.

Considering that the *S. invicta* GST primary sequence belongs to the sigma class, it was compared with the sigma-class GSTs from *H. sapiens* (*Hs*GSTS), *Am*GSTS, and *Bombus ignitus* (*Bi*GSTS). Additionally, delta-class *Am*GSTD was included for broader comparison. Analysis performed by Clustal Omega (version 1.2.4) sustains that *S. invicta* sigma-class GST shares 60% sequence identity with *B. ignitus* sigma GST, 60% with *A. mellifera* sigma GST, 39% with *H. sapiens* sigma GST, and 18% with *A. mellifera* delta GST. These findings were consistent with the available literature, which suggests that GSTs of the same class share over 40% sequence identity, whereas those from different classes only share around 25% (Oakley, 2011). The thioredoxin domain residues tyrosine, glutamine, serine, and arginine are highly conserved in the sigma-class GSTs analyzed. Nevertheless, the exception was the arginine residue, which was replaced by a methionine in *Hs*GSTS. For the delta-class GST in *Am*GSTD, the key catalytic serine residue aligns with the pocket 2 arginine in *Hs*GSTS, which is replaced by leucine in another GSTS. Also, the tryptophan, aspartic acid, and lysine residues in *Hs*GSTS were highly conserved in the sigma-class GSTs. From these findings, two residues can be used as insecticidal targets in sigma GST. These residues, leucine and glutamine in *Si*GSTS, correspond to tyrosine (pocket 2) and lysine (pocket 3) in *Hs*GSTS, and to phenylalanine and methionine in pollinator GSTs (*Bi*GSTS and *Am*GSTS), respectively. **Figure 5** shows the alignments already discussed, highlighting species-specific differences in the composition of these binding pockets. The overlapping three-dimensional structures of RIFA and human GSTs revealed clear coincidences, as shown in **Figure 6**. Nevertheless, the active-site residues differ and show punctual differences, which may be usable for developing insecticidal targets using sigma-class GSTs. In RIFA, these residues include L14, R99, L102, L154, and Q201, corresponding to R14, M99, F102, Y152, and K198 in humans (**Table 6**). An additional strategy to identify insecticidal blanks within GSTs could involve class-specific targeting based on delta and epsilon classes, which are restricted to insects (Oakley, 2011). In light of the GSTs’ molecular characterization, **Supplementary Table 6** presents selected evidence on the inhibitory activity of plant preparations with insecticidal or repellent effects against *Solenopsis* spp. In addition, **Figure 7** shows a diagram of the cellular localization of AChE and GST enzymes.

**Table 6:** Main residues of the GST tridimensional structures.

Site	SiGSTS	BiGSTS	HsGSTS	AmGSTD
3D-Structure code	AlphaFold PDB: Q3HVI8	AlphaFold PDB: D6R445	PDB: 3VI7	PDB: 7RHP
Catalytic residue	Y8	Y8	Y8	S34
G-site	Q63, S64	Q63, S64	Q63, S64	E89, S90
H-site	–	–	–	F142, Y138, F229, M134
Pocket site 1	R99, L102, W107	R99, Y102, W107	M99, F102, W104	–
Pocket site 2	L14, D96, L154	L14, D96, F153	R14, D96, Y152	–
Pocket site 3	K118, Q201	K118, M200	K112, K198	–

## 6 Side effects of non-target organisms

It is well known that synthetic pesticides show a broad-spectrum of toxic effects, often affecting non-target organisms such as pollinators, aquatic species, and soil microbiota, leading to ecological imbalances and biodiversity loss (Samanta et al., 2023). In contrast, plant-derived biopesticides tend to be more selective in their mode of action, causing fewer side effects to non-target organisms than conventional agrochemicals (Ayilara et al., 2023). Nevertheless, recent studies have shown that certain biopesticides can also exert adverse effects on non-target organisms. However, these impacts are generally less persistent and depend on specific doses, and they are environmentally reversible if compared with conventional agrochemicals (Seni, 2023). Because of these facts, natural biopesticides are still a safe alternative while further optimization is pursued (Seni, 2023). To contextualize this comparison, this section presents findings on the use of natural biocide or repellent compounds against *Solenopsis* spp., which can indirectly affect other insect species.

Among the most extensively studied plant-derived compounds, azadirachtin is the main limonoid from the *Azadirachta indica* plant. Azadirachtin affected flight abilities in *A. mellifera* and triggers a mortality rate of 40% in workers at 25-250 µL/L (Naiara Gomes et al., 2020). Field experiments have shown that azadirachtin reduces the flower-visiting rates of *A. mellifera* and *Halictus* sp., whereas *Plebeia* sp. bees are apparently unaffected (Tschoeke et al., 2019). Overall, bees are not repelled by azadirachtin, and they may collect contaminated food during foraging, leading to adverse sublethal effects after ingestion (Naiara Gomes et al., 2020). Doses of 100 mg/L of azadirachtin produce a mortality rate over 90% in *Apis cerana* workers (Zhao et al., 2022). This wide range of lethal and sublethal responses suggests a narrow safety margin that is strongly concentration-dependent across bee species. It is also observed that azadirachtin exerts sublethal effects on *A. cerana cerana* workers at 5–10 mg/L, accompanied by a significant reduction in gene expression or inhibition of midgut antioxidant enzyme polyphenol oxidase, *apidaecin*, *abaecin*, *defensin 2*, and *hymenoptaecin* genes, which are linked to immune responses. In addition, azadirachtin affects a variety of metabolic pathways, particularly leading to a significant decrease in mRNA levels of lysozyme, glucose-1-dehydrogenase, and alpha-amylase (Zhao et al., 2022). Nevertheless, azadirachtin did not affect the midgut microbiome or midgut antioxidant enzymes, such as superoxide dismutase and catalase (Zhao et al., 2022). This selective physiological impact highlights the complexity of interpreting toxicity beyond survival endpoints. In workers of the *Melipona quadrifasciata* stingless bee, the ingestion of azadirachtin at sublethal concentrations had no effects on the immune-related gene *abaecin* and superoxide dismutase expression but significantly decreased *vitellogenin* gene expression (Viana et al., 2021). The impregnation of surfaces with high concentrations of azadirachtin causes irritation in *M. quadrifasciata* workers, reducing their flower foraging capacity (Bernardes et al., 201*A. mellifera*7). Azadirachtin caused no mortality in *Partamona helleri* adult workers after superficial contact and oral exposure, but it inhibited feeding in *P. helleri* stingless bees in a dose-dependent way (Bernardes et al., 2017). This decrease in foraging behavior unravels a latent ecological concern that should be further addressed. The ingestion of azadirachtin at 24–48 ng did not cause high mortality effects in *P. helleri* queens, whereas doses of 240–480 ng resulted in a substantial mortality rate. High doses of this limonoid caused developmental delay and reproductive system deformities, potentially affecting colony survival (Bernardes et al., 2018). Consequently, reproductive impairment may represent a more critical ecological endpoint than worker mortality itself. Similarly, a 5% aqueous *Matricaria chamomilla* extract produced survival rates of 10 and 20% in *A. mellifera* after 200 hours contact and ingestion, respectively (Potrich et al., 2020). Nanoemulsions of peppermint essential oil (*Mentha piperita*) have also shown toxic effects on *A. mellifera* workers, resulting in higher mortality (LC_50_ = 4246.84 ppm) than the corresponding crude extract after oral or contact exposure (Youssef and Abdelmegeed, 2021). In contrast, garlic (*A. sativum*) essential oil nanoemulsion was non-toxic to *A. mellifera* workers during topical application experiments (Modafferi et al., 2025). This observation may suggest that new formulations based on the mixture of several materials can significantly alter the toxicity degree compared to crude extracts. Nevertheless, more studies are required to state or discard possible toxicity correlations based on case-by-case studies. In parallel, the *Artemisia annua* essential oil causes a high mortality rate in the melonworm second-instar *Diaphania hyalinata* larvae (96%; LD_95_ = 51.54 µg/mg) in comparison with its natural enemy *S. saevissima* (42%), and the pollinator bee *T. angustula* (74%). The individual components camphor (7.56 µg/mg) and 1,8-cineole (0.57 µg/mg) were putatively involved in insecticidal activity (Seixas et al., 2018). In a similar manner, *Lippia gracilis* essential oil, as well as its major compounds thymol and carvacrol, were effective insecticides for controlling *D. hyalinata*. *L. gracilis* essential oil produced 90% mortality on the second-instar of *D. hyalinata* larvae at 20 µg/mg, while thymol and carvacrol were more effective insecticides (LD_80_ = 5.17 and 3.21 µg/mg, respectively). Nevertheless, *L. gracilis* essential oil and its major compounds affected the pollinator *A. mellifera* and the wasp *Polybia micans*, resulting in mortality exceeding 80% (Melo et al., 2018). Furthermore, *L. gracilis* essential oil exhibited stronger toxicity against the coconut pest mites *Aceria guerreronis* (LD_50_ = 4.28 mg/mL) and *Raoiella indica* (LD_50_ = 4.99 mg/mL) than thymol (52%), its major constituent, which showed LC_50_ values of 5.34 and 9.03 mg/mL for *A. guerreronis* and *R. indica*, respectively (Dos Santos et al., 2019b). However, it also affected the predatory mite *Amblyseius largoensis*, causing 48% mortality at the LC_50_ determined for *R. indica* (Dos Santos et al., 2019b). The observed overlapping between target and beneficial insects raises the need for careful biopesticide selectivity assessment prior to field implementation.

The essential oils from *Litsea cubeba* and *Cinnamomum zeylanicum* seem to be less aggressive for non-target organisms than other essential oils. Interestingly, around 70% mortality was induced by both essential oils in *Varroa destructor* according to the glass-vial residual bioassay (Hýbl et al., 2021). Notably, these essential oils were innocuous to adult honeybee workers, with LC_50_ values of 4.8 and 11.7 µL of *L. cubeba* and 4.3 and 10.6 µL of *C. zeylanicum* for *V. destructor* and *A. mellifera*, respectively (Hýbl et al., 2021). Unfortunately, assayed amounts are reported as a sole volume of essential oil (µL per vial) instead of doses expressed in weight/volume or surface concentrations. This fact may be considered an obstacle to reproducing claimed findings. On the other hand, the essential oils of cinnamon (*Cinnamomum verum*), clove (*Syzygium aromaticum*), peppermint (*Mentha* sp.), and garlic (*A. sativum*) were effective in controlling wax moth larvae (*Galleria mellonlla*) and relatively safe for *A. mellifera* workers, producing a 9-16% mortality rate after 72-hour treatment (Helaly et al., 2022). Furthermore, a study evaluated the effects of a commercially essential oil blend (EcoTec+) and four individual volatiles, including β-bisabolene, cinnamaldehyde, 1,8-cineole, and eugenol, on worker honeybees (Caren et al., 2025). After 48 h of treatment, cinnamaldehyde caused 45% mortality at 8.5 mg/mL and 89% mortality at 68.5 mg/mL, whereas eugenol showed an LC_50_ of 86 mg/mL, causing 90% mortality at 135.0 mg/mL (Caren et al., 2025). Additionally, EcoTec+ (composed of 10% rosemary oil, 5% geraniol, and 2% peppermint oil) increased the expression of cytochrome P450 coding sequences. Similarly, bisabolene inhibited EST and AChE, increased GST levels, and triggered the synthesis of P450 enzymes. In contrast, cinnamaldehyde was toxic after ingestion, suppressed cytochrome P450 activity, and showed mixed inhibition on AChE (Caren et al., 2025). Likewise, cineole inhibited EST activity and reduced P450 gene expression after contact exposure, while inducing an increase in P450 transcripts after ingestion. Moreover, eugenol was found to be topically toxic and suppressed the expression of EST and AChE (Caren et al., 2025). These findings indicate that essential oil constituents differentially modulate detoxification enzymes and neural targets in honeybees. Overall, observed toxic effects were triggered at extremely high doses, considering a risk overestimation for honeybees under field conditions. Moreover, essential oils show differential effects on non-target organisms, and their reproducibility should be influenced by dose, chemical composition, and application methods. Unlike synthetic pesticides, plant-derived biopesticides generally exhibit low persistence and limited potential for resistance development. Nevertheless, their practical deployment is constrained by rapid volatilization and variable field performance. Large-scale applications would require improved formulations, sustainable platforms to produce them, as well as standardized doses, regulation criteria, and agricultural frameworks to be utilized under open field conditions.

## 7 Alternative methods for the application of semiochemical-based formulations

The use of baits in the management and control of *Solenopsis* spp. is an outstanding alternative that leverages the attractiveness, repellency, and biocidal properties of essential oils and semiochemicals (Chen and Oi, 2020). In this context, bait supplemented with repellent compounds prevents RIFA invasion of offices, homes, and plots. In contrast, the use of attractants enhances the efficiency of baits pre-loaded with toxic compounds (Guan et al., 2014). Two bait formulations, named Arino-su-korori (a granule-formulated bait containing hydramethylnon) and Hyper Arino-su-korori (a paste-formulated bait containing fipronil), were developed in Japan for the RIFA population control. Both formulations resulted in 99% mortality among RIFA workers after 8 weeks under laboratory conditions and reduced foraging under field conditions (Yasudai et al., 2022). Similarly, baits supplemented with a mixture of *cis-* and *trans*-allofarnesene (10 μg/μL) efficiently attracted up to 68% of RIFA workers, compared with those containing 2-ethyl-3,5-dimethylpyrazine (10 μg/μL or 100 μg/μL) that attracted 40-50% of workers within the first 30 minutes (Gokulanathan et al., 2024). The addition of higher (100 or 1000 μg/μL) or lower (1 μg/μL) concentrations of either mixture to baits reduced worker attraction (less than 40% or 20% for *cis*- and *trans*-allofarnesene, or less than 30% for 2-ethyl-3,5-dimethylpyrazine). However, bait attraction was affected by time and distance, decreasing as both parameters increased (Gokulanathan et al., 2024). Sliced sausage is commonly used to assess the attraction or repellency of RIFA workers to semiochemicals because of its effectiveness, low cost, and ease of management (Li et al., 2019; Shen et al., 2025*).* The effectiveness of baits for recruitment was improved by adding anethole, 1S-(−)-β-pinene, and β-caryophyllene at a concentration of 0.1 µg/µL (Li et al., 2019; Shen et al., 2025). Similarly, sausage baits enhanced worker attraction when supplemented with 100 μg of 2-ethyl-3,5-dimethylpyrazine or its isomers 2-ethyl-3,6-dimethylpyrazine, 2,3,5-trimethylpyrazine, and 2,3-diethyl-5-methylpyrazine (Guan et al., 2014). The compound 2-ethyl-3,5-dimethylpyrazine, along with its isomers, triggered antennae responses in electroantennography assays at concentrations ranging from 1 to 1000 μg. Notably, the optimal efficiency was observed at 100 and 1000 μg (Guan et al., 2014). Therefore, determining the critical concentrations of semiochemicals, their analogs, and essential oils that affect fire ant responses is crucial for designing strategies for the application of plant-derived biopesticides. Beyond bait formulation, advances in delivery systems may further improve the stability and effectiveness of strategies based on semiochemicals. Nanoencapsulation is a technology that has been explored for the delivery and protection of semiochemicals and essential oils (Sakamoto and Hashimoto, 2024; Nabi et al., 2025). For example, nanoparticles loaded with allyl isothiocyanate, a repellent and biocidal compound derived from wasabi (*Eutrema japonicum*), were used to prevent the invasion of RIFA in sea containers used for exportation (Sakamoto and Hashimoto, 2024). Sheets embedded with these nanoparticles significantly reduced the survival rate of RIFA ants to just 0.01% after seven days of transport in sea containers (Sakamoto and Hashimoto, 2024). These results highlight the importance of evaluating such delivery systems under field conditions. Overall, these findings support the potential of nanotechnology to deliver plant-derived repellents and biopesticides, limiting fire ant population and spreading.

## 8 Challenges and opportunity areas for plant volatiles and essential oils

A clear disadvantage of using essential oils and semiochemicals for pest control is their limited availability and high costs. Producing essential oils and semiochemicals in sufficient quantities to meet market demand is one of the most important challenges to be faced (Chen and Oi, 2020; Nabi et al., 2025). These limitations have dramatically influenced the low commercialization of semiochemical- and essential-oil-based biopesticides and repellents (Chen and Oi, 2020). To address these constraints, biotechnological approaches hold promise for improving the yields of semiochemicals and plant essential oils (Kant and Kumar, 2022; Nabi et al., 2025). To date, microorganisms and plant tissue cultures can serve as factories for synthesizing semiochemicals through metabolic pathway reconstruction and/or plant elicitation (Nabi et al., 2025). In addition, these methods can be complemented by cutting-edge metabolic engineering tools such as CRISPR, which can optimize biosynthetic pathways in both plants and microorganisms to produce bioactive compounds in large amounts (Nabi et al., 2025). Alongside improvements in biosynthesis, advances in extraction technologies can also increase the availability of plant-derived compounds. Non-conventional methods for essential oil extraction, such as enzyme-assisted ultrasound extraction, molecular distillation, and solar distillation, are now considered more efficient techniques to increase yields of volatile fractions (Kant and Kumar, 2022; Nabi et al., 2025). These novel methods spend less energy than conventional methods, require fewer toxic carriers such as deep eutectic solvents, and produce lower CO_2_ emissions. Eco-friendly techniques include supercritical fluid extraction, solvent-free microwave extraction, ultrasound-assisted extraction, microwave-assisted hydrodistillation, microwave-generated hydrodistillation, and microwave hydro-diffusion. Furthermore, non-conventional methods could aid in preserving essential oil bioactivity for production at an industrial scale (Kant and Kumar, 2022; Nabi et al., 2025).

Another strategy to overcome the limited availability of natural compounds is the use of structurally related synthetic analogs. The use of commercially available isomers and analogs of plant and insect semiochemicals that produce similar effects on RIFA behavior or toxicity is a plausible alternative in the short term (Li et al., 2019; Du et al., 2021a). For example, it was demonstrated that 2-ethyl-3,6-dimethylpyrazine, 2,3,5-trimethylpyrazine, and 2,3-diethyl-5-methylpyrazine attracted RIFA workers according to electrophysiological responses in the workers’ antennae (Guan et al., 2014). Similarly, the structure and size of acetate ester isomers and analogs seem to affect their interaction with olfactory proteins. Among acetate esters, those with shorter (one to four carbons) or longer (eight to twelve carbons) carbon chains do not elicit behavioral responses in any RIFA caste (Du et al., 2021a). However, linear alkyl acetate esters containing five to seven carbon atoms, such as pentyl acetate, isopropyl acetate, 2-methylbutyl acetate, hexyl acetate, and heptyl acetate, elicit strong electroantennography responses in RIFA workers, males, and females (Du et al., 2021a). Additionally, alkenyl acetates such as prenyl acetate, *cis*-3-hexenyl acetate, 3-methyl-3-butenyl acetate, and *trans*-2-hexenyl acetate exhibit a similar effect in workers and females. Ethoxyl, benzyl-substituted alkyl acetates, and aromatic acetates such as 2-ethoxyethyl acetate, benzyl acetate, β-phenethyl acetate, ethyl phenylacetate, and p-cresol acetate produce a similar electroantennography response (Du et al., 2021a). Pentyl acetate and *trans*-2-hexenyl acetate were stronger attractants, whereas hexyl acetate and cis-3-hexenyl acetate were outstanding repellents for all RIFA castes. Notably, the strongest effect was recorded in workers and queens (Du et al., 2021b). Benzyl acetate and prenyl acetate were initially identified in ylang ylang oil extracted from the flowers of *Cananga odorata*, both eliciting a potent electroantenographic response in alate males compared to alate females and workers and strongly attracting all three castes in choice and digging assays (Du et al., 2021b). Overall, these studies emphasize the wide variety of semiochemical analogs for use in fire ant population control. Nevertheless, translating laboratory findings into industrial-scale production remains a critical step that depends on technological maturity.

## 9 Conclusions and perspectives

The global spread of *Solenopsis* spp., particularly *S. invicta*, continues to impose substantial ecological, agricultural, and economic costs, underscoring the need for safer, more sustainable control strategies. Plant-derived biopesticides, especially essential oils, volatile compounds, and semiochemical-based formulations, offer considerable promise for fire ant management. Their value lies not only in insecticidal activity, but also in their capacity to disrupt key behaviors such as foraging, aggregation, digging, trail following, and colony organization. Recent advances in the molecular understanding of olfactory proteins in *Solenopsis* spp. aided in establishing the fundamentals for olfactory system disruption for behavior-based pest control. OBPs and CSPs mediate the detection of pheromones and environmental semiochemicals that regulate fire ants’ behavioral responses. For example, *Si*OBP2 and *Si*OBP14 are associated with foraging behavior, whereas *Si*OBP15 is linked to sanitation behavior and social immunity, highlighting these proteins as promising molecular targets for semiochemical-based biopesticide strategies. However, key components of the olfactory pathway, including ORs and Orco, remain poorly characterized and require further investigation to elucidate transduction pathways to provide a temporal and causal sequence that elicits behavioral responses in fire ants. In contrast, enzymatic targets such as AChE and GST are better characterized and fully addressed in this review. However, additional molecular targets may further broaden the range of potential biocidal strategies. Together, these molecular targets support a transition from broad-spectrum chemical suppression toward more selective and behavior-informed control strategies.

Plant-based biopesticides, including extracts, chemical fractions, and sole natural products, have demonstrated strong insecticidal effects against *Solenopsis* spp. Azadirachtin, celangulin, octanal, α-pinene, citral, isothiocyanates, carvacrol derivatives, and eugenol analogs exhibited strong lethality on *Solenopsis* spp. Additionally, essential oils and volatiles from *I. verum*, *B. balsamifera*, *L. cubeba*, *C. loureirii*, *A. sativum*, *C. longa*, and *B. microdonta* have demonstrated rapid and high mortality across different ant castes under simulated open-field conditions. However, no single plant-derived compound currently provides an integrative or infallible solution. The available evidence points to complementary modes of action based on compounds that induce rapid mortality, combined with others exhibiting high potency at low doses, which may exert repellency or sustained-release activity to optimize efficacy, persistence, and environmental compatibility. For instance, volatile-rich compounds such as methyl eugenol may provide rapid knockdown during early infestation stages, whereas plant-derived matrices containing persistent volatiles may sustain longer-term insecticidal and repellent activity under field conditions. Accordingly, future management of *Solenopsis* spp. will likely benefit most from integrated formulations that combine toxic, repellent, and attractive properties with baiting systems and improved delivery platforms.

Several challenges still limit field deployment, including variability in chemical composition, rapid volatilization, variable performance under open field conditions, and undesired side effects. Toxicity reported for most of the essential oils described in this review widely varies and depends on the assayed concentration, formulation, and evaluation protocols. Then, there is an increasing need to establish validated protocols to normalize toxicity measurements in *Solenopsis* spp. castes. Although some essential oil components alter detoxifying and neural enzyme activity in bees, most show low direct lethality. Overall, biopesticides offer a promising alternative for integrated pest management, but their ecological safety should be carefully evaluated on a case-by-case basis, depending on the target organism. In parallel, large-scale applications remain constrained by production costs and the limited availability of natural compounds. In this sense, the use of low-cost isomers and analogs in repellent and biopesticide formulations, such as 2,3-diethyl-5-methylpyrazine and acetate ester derivatives, is a viable alternative. Future research should therefore prioritize formulation optimization (e.g., nanoencapsulation) of controlled-release systems, scalable production through biotechnology and metabolic engineering, and rigorous field validation under realistic ecological conditions. Overall, plant volatiles and semiochemical-inspired formulations represent a promising platform for the sustainable management of imported fire ants. Advancing this field will require tighter integration of natural product chemistry, insect chemical ecology, molecular target characterization, and formulation science to translate laboratory evidence into selective and scalable pest-control tools.

## References

[B1] Addesso K. M. Oliver J. B. O’Neal P. A. Youssef N. (2017). Efficacy of nootka oil as a biopesticide for management of imported fire ants (Hymenoptera: Formicidae). J. Econ. Entomol. 110, 1547–1555. doi: 10.1093/jee/tox114 28402466

[B2] Advanced Chemistry Development, Inc (2023). ACD/Chemsketch (Version 2023.1) (Toronto, ON, Canada: Advanced Chemistry Development, Inc.).

[B3] Ali A. Chen J. Khan I. A. (2022). Toxicity and repellency of Magnolia grandiflora seed essential oil and selected pure compounds against the workers of hybrid imported fire ants (Hymenoptera: Formicidae). J. Econ. Entomol. 115, 412–416. doi: 10.1093/jee/toab262 35048988

[B4] Ali A. Shah F. M. Manfron J. Monteiro L. M. de Almeida V. P. Raman V. . (2023). Baccharis species essential oils: Repellency and toxicity against yellow fever mosquitoes and imported fire ants. J. Xenobiot. 13, 641–652. doi: 10.3390/jox13040041 37987442 PMC10660731

[B5] Ali A. Shah F. M. Radwan M. M. Elhendawy M. A. Elsohly M. A. Khan I. A. (2024). Curcuma longa essential oils: toxicity and repellency against imported fire ants (Formicidae: Hymenoptera). J. Med. Entomol. 61, 191–200. doi: 10.1093/jme/tjad151 37983140

[B6] Alvarez F. M. Vander Meer R. K. Lofgren C. S. (1987). Synthesis of homofarnesenes: trail pheromone components of the fire ant, Solenopsis invicta. Tetrahedron 43, 2897–2900. doi: 10.1016/s0040-4020(01)86827-7

[B7] Angulo E. Hoffmann B. D. Ballesteros-Mejia L. Taheri A. Balzani P. Bang A. . (2022). Economic costs of invasive alien ants worldwide. Biol. Invasions 24, 2041–2060. doi: 10.1007/s10530-022-02791-w 30311153

[B8] Atkinson H. J. Babbitt P. C. (2009). Glutathione transferases are structural and functional outliers in the thioredoxin fold. Biochemistry 48, 11108–11116. doi: 10.1021/bi901180v 19842715 PMC2778357

[B9] Ayilara M. S. Adeleke B. S. Akinola S. A. Fayose C. A. Adeyemi U. T. Gbadegesin L. A. . (2023). Biopesticides as a promising alternative to synthetic pesticides: A case for microbial pesticides, phytopesticides, and nanobiopesticides. Front. Microbiol. 14, 1040901. doi: 10.3389/fmicb.2023.1040901 36876068 PMC9978502

[B10] Bernardes R. C. Barbosa W. F. Martins G. F. Pereira-Lima M. A. (2018). The reduced-risk insecticide azadirachtin poses a toxicological hazard to stingless bee Partamona helleri (Friese 1900) queens. Chemosphere 201, 550–556. doi: 10.1016/j.chemosphere.2018.03.030 29533804

[B11] Bernardes R. C. Tomé H. V. Barbosa W. F. Guedes R. N. Pereira-Lima M. A. (2017). Azadirachtin-induced antifeeding in Neotropical stingless bees. Apidologie 48, 275–285. doi: 10.1007/s13592-016-0473-3 30311153

[B12] Brindis Y. Lachaud J. P. Gómez Y Gómez B. Rojas J. C. Malo E. A. Cruz-López L. (2008). Behavioral and olfactory antennal responses of Solenopsis geminata (Fabricius)(Hymenoptera: Formicidae) workers to their dufour gland secretion. Neotrop. Entomol. 37, 131–136. doi: 10.1590/s1519-566x2008000200004 18506290

[B13] Caren J. Zhu Y. C. Read Q. D. Du Y. (2025). Risk assessment of effects of essential oils on honey bees (Apis mellifera L.). Insects 16, 303. doi: 10.3390/insects16030303 40266795 PMC11942678

[B14] Chan K. H. Guénard B. (2020). Ecological and socio-economic impacts of the red import fire ant, Solenopsis invicta (Hymenoptera: Formicidae), on urban agricultural ecosystems. Urban Ecosyst. 23, 1–12. doi: 10.1007/s11252-019-00893-3 30311153

[B15] Chen J. Oi D. H. (2020). Naturally occurring compounds/materials as alternatives to synthetic chemical insecticides for use in fire ant management. Insects 11, 758. doi: 10.3390/insects11110758 33158097 PMC7694179

[B16] Cheng D. Lu Y. Zeng L. Liang G. He X. (2015). Si-CSP9 regulates the integument and moulting process of larvae in the red imported fire ant, Solenopsis invicta. Sci. Rep. 5, 9245. doi: 10.1038/srep09245 25784646 PMC4363891

[B17] Cohanim A. B. Amsalem E. Saad R. Shoemaker D. Privman E. (2018). Evolution of olfactory functions on the fire ant social chromosome. Genome Biol. Evol. 10, 2947–2960. doi: 10.1093/gbe/evy204 30239696 PMC6279166

[B18] Cohen P. Privman E. (2020). The social supergene dates back to the speciation time of two Solenopsis fire ant species. Sci. Rep. 10, 11538. doi: 10.1038/s41598-020-67999-z 32665692 PMC7360596

[B19] Dang V. D. Cohanim A. B. Fontana S. Privman E. Wang J. (2019). Has gene expression neofunctionalization in the fire ant antennae contributed to queen discrimination behavior? Ecol. Evol. 9, 12754–12766. doi: 10.1002/ece3.5748 31788211 PMC6875580

[B20] Dantas J. O. Cavalcanti S. C. H. Araújo A. P. A. Blank A. F. Silva J. E. Picanço M. C. . (2023). Synthetic carvacrol derivatives for the management of Solenopsis ants: toxicity, sublethal effects, and horizontal transfer. Agriculture 13, 1988. doi: 10.3390/agriculture13101988 30654563

[B21] Dos Santos A. M. Moreira A. C. Lopes B. R. Fracola M. F. de Almeida F. G. Bueno O. C. . (2019a). Acetylcholinesterases from Leaf‐Cutting ant Atta sexdens: Purification, Characterization, and Capillary Reactors for On‐Flow Assays. Enzyme Res. 2019, 6139863. doi: 10.1155/2019/6139863 31354985 PMC6633970

[B22] Dos Santos M. C. Teodoro A. V. Menezes M. S. Pinto-Zevallos D. M. Arrigoni-Blank M. F. Cruz-Oliveira E. M. . (2019b). Bioactivity of essential oil from Lippia gracilis Schauer against two major coconut pest mites and toxicity to a non-target predator. Crop Prot. 125, 104913. doi: 10.1016/j.cropro.2019.104913 38826717

[B23] Du Y. Chen J. (2021). The odorant binding protein, SiOBP5, mediates alarm pheromone olfactory recognition in the red imported fire ant, Solenopsis invicta. Biomolecules 11, 1595. doi: 10.3390/biom11111595 34827593 PMC8615367

[B24] Du Y. Grodowitz M. J. Chen J. (2020). Insecticidal and enzyme inhibitory activities of isothiocyanates against red imported fire ants, Solenopsis invicta. Biomolecules 10, 716. doi: 10.3390/biom10050716 32380698 PMC7277602

[B25] Du Y. Zhou A. Chen J. (2021a). Olfactory and behavioral responses to acetate esters in red imported fire ant, Solenopsis invicta. Pest Manage. Sci. 77, 1371–1382. doi: 10.1002/ps.6152 33089649

[B26] Du Y. Zhou A. Chen J. (2021b). Olfactory and behavioral responses of red imported fire ants, Solenopsis invicta, to ylang ylang oil and its components. J. Pest Sci. 94, 1031–1044. doi: 10.1007/s10340-020-01312-9 30311153

[B27] Dvir H. Silman I. Harel M. Rosenberry T. L. Sussman J. L. (2010). Acetylcholinesterase: from 3D structure to function. Chem. Biol. Interact. 187, 10–22. doi: 10.1016/j.cbi.2010.01.042 20138030 PMC2894301

[B28] EFSA Panel on Plant Health (PLH) Bragard C. Baptista P. Chatzivassiliou E. Di Serio F. Gonthier P. (2023). Pest categorisation of Solenopsis invicta. EFSA J. 21 (5), e07998. doi: 10.2903/j.efsa.2023.7998 37234270 PMC10205889

[B29] Enayati A. A. Ranson H. Hemingway J. (2005). Insect glutathione transferases and insecticide resistance. Insect Mol. Biol. 14, 3–8. doi: 10.1111/j.1365-2583.2004.00529.x 15663770

[B30] Epperson D. M. Allen C. R. Hogan K. F. E. (2020). Red imported fire ants reduce invertebrate abundance, richness, and diversity in gopher tortoise burrows. Diversity 13, 7. doi: 10.3390/d13010007 30654563

[B31] Fan M. Ye T. Wang Z. Li Q. Li C. Shi Q. . (2024). The repellent effects of eight Chinese herbal essential oils on red imported fire ants, Solenopsis invicta Buren (Hymenoptera: Formicidae), and analysis of active components. Ind. Crop Prod. 219, 119111. doi: 10.1016/j.indcrop.2024.119111 38826717

[B32] Flores-Hernández M. (2024). Evaluación del aceite esencial de Piper auritum (hoja santa) y sus principales volátiles en el control de la hormiga de fuego Solenopsis geminata. Benemérita Universidad Autónoma de Puebla, Puebla (Pue. Mexico.

[B33] Friedman R. (2011). Genomic organization of the glutathione S-transferase family in insects. Mol. Phylogenet. Evol. 61, 924–932. doi: 10.1016/j.ympev.2011.08.027 21930223

[B34] Fu J. Ma Z. Wang L. Zhang Y. Luo Y. (2023). Fumigant toxicity and behavioral alterations of six plant essential oils against the red fire ant (Solenopsis invicta Buren). Environ. Sci. pollut. Res. 30, 68677–68690. doi: 10.1007/s11356-023-27329-y 37126171

[B35] Gerlits O. Kong X. Cheng X. Wymore T. Blumenthal D. K. Taylor P. . (2019). Productive reorientation of a bound oxime reactivator revealed in room temperature X-ray structures of native and VX-inhibited human acetylcholinesterase. J. Biol. Chem. 294, 10607–10618. doi: 10.1074/jbc.RA119.008725 31138650 PMC6615692

[B36] Gokulanathan A. Mo H. H. Park Y. (2024). Attraction behavior of the red imported fire ant, Solenopsis invicta Buren, to candidate attractants and poisoning baits. J. Asia-Pac. Entomol. 27, 102347. doi: 10.1016/j.aspen.2024.102347 38826717

[B37] González D. Zhao Q. McMahan C. Velasquez D. Haskins W. E. Sponsel V. . (2009). The major antennal chemosensory protein of red imported fire ant workers. Insect Mol. Biol. 18, 395–404. doi: 10.1111/j.1365-2583.2009.00883.x 19523071 PMC2771726

[B38] Gotzek D. Robertson H. M. Wurm Y. Shoemaker D. (2011). Odorant binding proteins of the red imported fire ant, Solenopsis invicta: an example of the problems facing the analysis of widely divergent proteins. PloS One 6, e16289. doi: 10.1371/journal.pone.0016289 21305009 PMC3031547

[B39] Guan D. Lu Y. Y. Liao X. L. Wang L. Chen L. (2014). Electroantennogram and behavioral responses of the imported fire ant, Solenopsis invicta Buren, to an alarm pheromone component and its analogues. J. Agric. Food. Chem. 62, 11924–11932. doi: 10.1021/jf505191s 25415443

[B40] Guo Y. B. Liu Z. J. Ye Y. Y. Chen Y. Y. Wu Z. W. (2025). Antifeedant and contact toxicity activity of crude extracts from 10 plants to red imported fire ant, Solenopsis invicta Buren (Hymenoptera: Formicidae). J. Asia-Pac. Entomol. 28, 102350. doi: 10.1016/j.aspen.2024.102350 38826717

[B41] Hamdeni I. Boukhris-Bouhachem S. Louhaichi M. Boulila A. Amri I. Coque J. J. R. . (2026). Synergistic and additive interactions in essential oils obtained from combined plant materials: Enhanced control of insect pests. Molecules 31, 945. doi: 10.3390/molecules31060945 41900044 PMC13029542

[B42] Harel M. Kryger G. Rosenberry T. L. Mallender W. D. Lewis T. Fletcher R. J. . (2000). Three-dimensional structures of Drosophila melanogaster acetylcholinesterase and of its complexes with two potent inhibitors. Protein Sci. 9, 1063–1072. doi: 10.1110/ps.9.6.1063 10892800 PMC2144661

[B43] He Y. Zhang J. Shen L. Wang L. Qian C. Lyu H. . (2023). Eugenol derivatives: Strong and long-lasting repellents against both undisturbed and disturbed red imported fire ants. J. Pest Sci. 96, 327–344. doi: 10.1007/s10340-022-01501-8 30311153

[B44] Helaly E. G. Ali M. A. M. Ghazala N. A. (2022). Evaluation of some essential oils against wax moth larvae (Lepedoptera: Galleria mellonlla L.) and adult honeybee workers (Hymenoptera: Apis mellifera L). Arab. Univ. J. Agric. Sci. 30, 157–162. doi: 10.21608/ajs.2022.108974.1441

[B45] Hezakiel H. E. Thampi M. Rebello S. Sheikhmoideen J. M. (2024). Biopesticides: a green approach towards agricultural pests. Appl. Biochem. Biotechnol. 196, 5533–5562. doi: 10.1007/s12010-023-04765-7 37994977

[B46] Huang C. L. Fu J. T. Liu Y. K. Cheng D. M. Zhang Z. X. (2015). The insecticidal and repellent activity of soil containing cinnamon leaf debris against red imported fire ant workers. Sociobiology 62, 46–51. doi: 10.13102/sociobiology.v62i1.46-51

[B47] Huang R. L. Li Z. H. Wang S. Y. Fu J. T. Cheng D. M. Zhang Z. X. (2016). Insecticidal effect of volatile compounds from plant materials of Murraya exotica against red imported fire ant workers. Sociobiology 63, 783–791. doi: 10.13102/sociobiology.v63i2.972

[B48] Hýbl M. Bohatá A. Rádsetoulalová I. Kopecký M. Hoštičková I. Vaníčková A. . (2021). Evaluating the efficacy of 30 different essential oils against Varroa destructor and honey bee workers (Apis mellifera). Insects 12, 1045. doi: 10.3390/insects12111045 34821845 PMC8623799

[B49] Jiang X. Shen J. Lin P. Hou Y. (2025). High antennal expression of CYP6K1 and CYP4V2 participate in the recognition of alarm pheromones by Solenopsis invicta Buren. Insects 16, 43. doi: 10.3390/insects16010043 39859624 PMC11765799

[B50] Kado Y. Aritake K. Uodome N. Okano Y. Okazaki N. Matsumura H. . (2012). Human hematopoietic prostaglandin D synthase inhibitor complex structures. J. Biochem. 151, 447–455. doi: 10.1093/jb/mvs024 22418579

[B51] Kant R. Kumar A. (2022). Review on essential oil extraction from aromatic and medicinal plants: techniques, performance and economic analysis. Sustain. Chem. Pharm. 30, 100829. doi: 10.1016/j.scp.2022.100829 38826717

[B52] Kenfack J. M. Dzokou V. J. Djomaha E. S. Aoudou Y. Tamouh R. G. (2022). Impact of Solenopsis geminata pest (Hymenoptera: Formicidae), the tropical fire ant on soybean (Glycine max) production in Littoral, Cameroon. J. Entomol. Zool. Stud. 10, 10–18. doi: 10.22271/j.ento.2022.v10.i6a.9091

[B53] Kim Y. H. Lee S. H. (2013). Which acetylcholinesterase functions as the main catalytic enzyme in the class insecta? Insect Biochem. Mol. Biol. 43, 47–53. doi: 10.1016/j.ibmb.2012.11.004 23168079

[B54] Krieger M. J. Ross K. G. (2005). Molecular evolutionary analyses of the odorant-binding protein gene Gp-9 in fire ants and other Solenopsis species. Mol. Biol. Evol. 22, 2090–2103. doi: 10.1093/molbev/msi203 15987877

[B55] Kulmuni J. Wurm Y. Pamilo P. (2013). Comparative genomics of chemosensory protein genes reveals rapid evolution and positive selection in ant-specific duplicates. Heredity 110, 538–547. doi: 10.1038/hdy.2012.122 23403962 PMC3656642

[B56] Kurmanbayeva A. Ospanov M. Tamang P. Shah F. M. Ali A. Ibrahim Z. M. A. . (2023). Regioselective Claisen–Schmidt adduct of 2-undecanone from Houttuynia cordata Thunb as insecticide/repellent against Solenopsis invicta and repositioning plant fungicides against Colletotrichum fragariae. Molecules 28, 6100. doi: 10.3390/molecules28166100 37630353 PMC10458534

[B57] Lewis D. F. Ito Y. (2008). “ Cytochromes P450: role in the metabolism and toxicity of drugs and other xenobiotics,” in Cytochromes P450. Ed. Ioannides C. (Cambridge, UK: The Royal Society of Chemistry), 3–45. doi: 10.1039/9781847558428-00003

[B58] Li D. Li Z. Wang X. Wang L. Khoso A. G. Liu D. (2023). Climate change and international trade can exacerbate the invasion risk of the red imported fire ant Solenopsis invicta around the globe. Entomol. Gen. 43, 315–323. doi: 10.1127/entomologia/2023/1686

[B59] Li Y. Y. Liu D. Chen L. (2019). Electrophysiological and alarm responses of Solenopsis invicta Buren (Hymenoptera: Formicidae) to 2-ethyl-3, 5-dimethylpyrazine. Insects 10, 451. doi: 10.3390/insects10120451 31847156 PMC6955860

[B60] Li Y. Yu S. Huang J. Wang Z. Zeng Y. Wu X. . (2022). Study of behavioral, electrophysiological response, and the active compounds of the essential oils from six kinds of flowers against Solenopsis invicta Buren (Hymenoptera: Formicidae). Ind. Crop Prod. 188, 115603. doi: 10.1016/j.indcrop.2022.115603 38826717

[B61] Liang L. Li J. Jin L. Yan K. Pan Y. Shang Q. (2024). Identification of inducible CYP3 and CYP4 genes associated with abamectin tolerance in the fat body and Malpighian tubules of Spodoptera litura. Pestic. Biochem. Physiol. 198, 105751. doi: 10.1016/j.pestbp.2023.105751 38225094

[B62] Liang Y. Liang M. Chen H. Hong J. Song Y. Yue K. . (2023). The effect of botanical pesticides azadirachtin, celangulin, and veratramine exposure on an invertebrate species Solenopsis invicta (Hymenoptera: Formicidae). Toxins 16, 6. doi: 10.3390/toxins16010006 38276530 PMC10821215

[B63] Liu J. Zhao W. Hu C. Xia Y. Li L. Zhang F. . (2025). An antennal-specific OBP mediates bait odorant perception in fire ants. Int. J. Biol. Macromol. 293, 139416. doi: 10.1016/j.ijbiomac.2024.139416 39746423

[B64] Melo C. R. Picanço M. C. Santos A. A. Santos I. B. Pimentel M. F. Santos A. C. C. . (2018). Toxicity of essential oils of Lippia gracilis chemotypes and their major compounds on Diaphania hyalinata and non-target species. Crop Prot. 104, 47–51. doi: 10.1016/j.cropro.2017.10.013 38826717

[B65] Mier P. Fontaine J. F. Stoldt M. Libbrecht R. Martelli C. Foitzik S. . (2022). Annotation and analysis of 3902 odorant receptor protein sequences from 21 insect species provide insights into the evolution of odorant receptor gene families in solitary and social insects. Genes 13, 919. doi: 10.3390/genes13050919 35627304 PMC9141868

[B66] Modafferi A. Giunti G. Urbaneja A. Laudani F. Latella I. Pérez-Hedo M. . (2025). High-energy emulsification of Allium sativum essential oil boosts insecticidal activity against Planococcus citri with no risk to honeybees. J. Pest Sci. 98, 337–348. doi: 10.1007/s10340-024-01800-2 30311153

[B67] Morgan E. D. (2008). Chemical sorcery for sociality: exocrine secretions of ants (Hymenoptera: Formicidae). Myrmecol. News. 11, 79–90. doi: 10.25849/myrmecol.news_011:079

[B68] Moural T. W. BK S. K. Bhattarai G. He Z. Guo H. Phan N. T. . (2024). Architecture and potential roles of a delta-class glutathione S-transferase in protecting honey bee from agrochemicals. Chemosphere 350, 141089. doi: 10.1016/j.chemosphere.2023.141089 38163465

[B69] Nabi M. H. B. Ahmed M. M. Mia M. S. Islam S. Zzaman W. (2025). Essential oils: advances in extraction techniques, chemical composition, bioactivities, and emerging applications. Food Chem. Adv. 8, 101048. doi: 10.1016/j.focha.2025.101048 38826717

[B70] Nachon F. Rosenberry T. L. Silman I. Sussman J. L. (2020). A second look at the crystal structures of Drosophila melanogaster acetylcholinesterase in complex with tacrine derivatives provides insights concerning catalytic intermediates and the design of specific insecticides. Molecules 25, 1198. doi: 10.3390/molecules25051198 32155891 PMC7179448

[B71] Naiara Gomes I. Ingred Castelan Vieira K. Moreira Gontijo L. Canto Resende H. (2020). Honeybee survival and flight capacity are compromised by insecticides used for controlling melon pests in Brazil. Ecotoxicology 29, 97–107. doi: 10.1007/s10646-019-02145-8 31832831

[B72] Nalini T. Sasinathan S. (2020). Evaluation of toxicity of botanicals and essential oils against Solenopsis geminata (Fabricius)(Hymenoptera: Formicidae). Eco. Env. Cons. 26, 210–215.

[B73] Oakley A. (2011). Glutathione transferases: a structural perspective. Drug Metab. Rev. 43, 138–151. doi: 10.3109/03602532.2011.558093 21428697

[B74] Pang Y. P. Brimijoin S. Ragsdale D. W. Yan Zhu K. Suranyi R. (2012). Novel and viable acetylcholinesterase target site for developing effective and environmentally safe insecticides. Curr. Drug Targets 13, 471–482. doi: 10.2174/138945012799499703 22280344 PMC3343382

[B75] Paudel P. Shah F. M. Guddeti D. K. Ali A. Chen J. Khan I. A. . (2023). Repellency of carvacrol, thymol, and their acetates against imported fire ants. Insects 14, 790. doi: 10.3390/insects14100790 37887802 PMC10607101

[B76] Potrich M. da Silva R. T. L. Maciel R. M. A. Costa-Maia F. M. Lozano E. R. Rossi R. M. . (2020). Are plant extracts safe for honey bees (Apis mellifera)? J. Apic. Res. 59, 844–851. doi: 10.1080/00218839.2020.1735733 37339054

[B77] Pracana R. Levantis I. Martínez-Ruiz C. Stolle E. Priyam A. Wurm Y. (2017). Fire ant social chromosomes: differences in number, sequence and expression of odorant binding proteins. Evol. Lett. 1, 199–210. doi: 10.1002/evl3.22 30283649 PMC6121795

[B78] Qin D. Huang R. Li Z. Wang S. Cheng D. Zhang Z. (2018). Volatile component analysis of Michelia alba leaves and their effect on fumigation activity and worker behavior of Solenopsis invicta. Sociobiology 65, 170–176. doi: 10.13102/sociobiology.v65i2.2014

[B79] Qiu H. L. Cheng D. F. (2017). A chemosensory protein gene Si-CSP1 associated with necrophoric behavior in red imported fire ants (Hymenoptera: Formicidae). J. Econ. Entomol. 110, 1284–1290. doi: 10.1093/jee/tox095 28444203

[B80] Rana A. Sharma D. Choudhary K. Kumari P. Ruchika K. Yangchan J. . (2024). Insight into insect odorant binding proteins: an alternative approach for pest management. J. Nat. Pestic. Res. 8, 100069. doi: 10.1016/j.napere.2024.100069 38826717

[B81] Redmond K. M. Grahmann E. D. Hernández F. Brennan L. A. Morrow M. E. Anderson T. (2022). Northern bobwhite response to control of red imported fire ants in the Gulf Coast Prairie of Texas. Natl. Quail Symposium Proc. 9, 64. doi: 10.7290/nqsp09S1cE

[B82] Renthal R. Velasquez D. Olmos D. Hampton J. Wergin W. P. (2003). Structure and distribution of antennal sensilla of the red imported fire ant. Micron 34, 405–413. doi: 10.1016/S0968-4328(03)00050-7 14680927

[B83] Reyes-Ávila A. López-Ruiz R. González F. J. E. Romero-González R. Frenich A. G. (2024). Chemistry and development of bioinsecticides for safe and sustainable use. Curr. Opin. Environ. Sci. Health 41, 100568. doi: 10.1016/j.coesh.2024.100568 38826717

[B84] Rezende-Teixeira P. Dusi R. G. Jimenez P. C. Espindola L. S. Costa-Lotufo L. V. (2022). What can we learn from commercial insecticides? Efficacy, toxicity, environmental impacts, and future developments. Environ. pollut. 300, 118983. doi: 10.1016/j.envpol.2022.118983 35151812

[B85] Rizvi S. A. H. Li Y. Ullah R. M. K. Lu Y. (2025). Exploring the fumigant potential of Artemisia subg. Seriphidium essential oils and their dominant constituents against the red imported fire ants Solenopsis invicta. Ind. Crop Prod. 226, 120603. doi: 10.1016/j.indcrop.2025.120603 38826717

[B86] Saad R. Cohanim A. B. Kosloff M. Privman E. (2018). Neofunctionalization in ligand binding sites of ant olfactory receptors. Genome Biol. Evol. 10, 2490–2500. doi: 10.1093/gbe/evy131 29982411 PMC6161762

[B87] Sakamoto H. Hashimoto Y. (2024). Establishment and application of microencapsulated wasabi ingredients to prevent Solenopsis invicta from entering containers. Glob. Environ. Res. 28, 109–115.

[B88] Sakhanokho H. F. Sampson B. J. Tabanca N. Wedge D. E. Demirci B. Baser K. H. C. . (2013). Chemical composition, antifungal and insecticidal activities of Hedychium essential oils. Molecules 18, 4308–4327. doi: 10.3390/molecules18044308 23579997 PMC6270349

[B89] Samanta S. Maji A. Sutradhar B. Banerjee S. Shelar V. B. Khaire P. B. . (2023). Impact of pesticides on beneficial insects in various agroecosystem: a review. Int. J. Environ. Clim. Change 13, 1928–1936. doi: 10.9734/ijecc/2023/v13i82149 42260324

[B90] Seixas P. T. L. Demuner A. J. Alvarenga E. S. Barbosa L. C. A. Marques A. Farias E. D. S. . (2018). Bioactivity of essential oils from Artemisia against Diaphania hyalinata and its selectivity to beneficial insects. Sci. Agric. 75, 519–525. doi: 10.1590/1678-992X-2016-0461 41099703

[B91] Seni A. (2023). Potential of the various oils for insect pests’ management and their effect on beneficial insects. Int. J. Trop. Insect Sci. 43, 321–337. doi: 10.1007/s42690-023-00970-3 30311153

[B92] Shah F. M. Guddeti D. K. Paudel P. Chen J. Li X. C. Khan I. A. . (2023). Matricaria chamomilla essential oils: repellency and toxicity against imported fire ants (Hymenoptera: Formicidae). Molecules 28, 5584. doi: 10.3390/molecules28145584 37513455 PMC10384828

[B93] Shah J. S. Renthal R. (2020). Antennal proteome of the Solenopsis invicta (Hymenoptera: Formicidae): caste differences in olfactory receptors and chemosensory support proteins. J. Insect Sci. 20, 29. doi: 10.1093/jisesa/ieaa118 33098433 PMC7585320

[B94] Shen J. Wu S. Y. Lin P. Jiang X. Hou Y. (2025). Identification and optimization of volatile organic compounds to enhance bait attractiveness for red imported fire ants (Solenopsis invicta Buren). Pest Manage. Sci. 81, 3240–3249. doi: 10.1002/ps.8696 39906913

[B95] Silman I. Sussman J. L. (2008). Acetylcholinesterase: how is structure related to function? Chem. Biol. Interact. 175, 3–10. doi: 10.1016/j.cbi.2008.05.035 18586019

[B96] Song Y. Chen M. Wu J. Hong J. Ouyang T. Liang Y. . (2024). Impact on ant communities by chemical pesticides applied in controlling the red imported fire ant (Solenopsis invicta Buren) in the field. Insects 15, 876. doi: 10.3390/insects15110876 39590475 PMC11594959

[B97] Song Z. Wang Y. Li C. Tan Y. Wu J. Zhang Z. (2023). Fumigant toxicity and behavioral inhibition of garlic against red imported fire ants (Solenopsis invicta). Environ. Sci. pollut. Res. 30, 1889–1897. doi: 10.1007/s11356-022-22091-z 35927401

[B98] Souto R. N. P. Harada A. Y. Andrade E. H. A. Maia J. G. S. (2012). Insecticidal activity of Piper essential oils from the Amazon against the fire ant Solenopsis saevissima (Smith)(Hymenoptera: Formicidae). Neotrop. Entomol. 41, 510–517. doi: 10.1007/s13744-012-0080-6 23949677

[B99] Suckling D. M. Stringer L. D. Bunn B. El-Sayed A. M. Vander Meer R. K. (2010). Trail pheromone disruption of red imported fire ant. J. Chem. Ecol. 36, 744–750. doi: 10.1007/s10886-010-9810-6 20549330

[B100] Sun Y. Shao K. M. Lu Y. Y. Shi Q. H. Wang W. K. Chen L. (2017). Electrophysiological and alarm behavioral responses of Solenopsis invicta Buren (Hymenoptera: Formicidae) to alkoxypyrazines. J. Asia-Pac. Entomol. 20, 541–546. doi: 10.1016/j.aspen.2017.03.015 38826717

[B101] Swartwout M. C. Willson J. D. (2022). Southeastern US snake species are vulnerable to egg predation by red imported fire ants (Solenopsis invicta). Herpetologica 78, 139–144. doi: 10.1655/HERPETOLOGICA-D-21-00004 40071500

[B102] Tian Y. Zhang Z. (2023). Insecticidal activities of Sophora flavescens Alt. towards red imported fire ants (Solenopsis invicta Buren). Toxins 15, 105. doi: 10.3390/toxins15020105 36828419 PMC9965024

[B103] Travanty N. V. Vargo E. L. Schal C. Apperson C. S. Ponnusamy L. (2022). Bacterial isolates derived from nest soil affect the attraction and digging behavior of workers of the red imported fire ant, Solenopsis invicta Buren. Insects 13, 444. doi: 10.3390/insects13050444 35621779 PMC9145412

[B104] Tschoeke P. H. Oliveira E. E. Dalcin M. S. Silveira-Tschoeke M. C. A. C. Sarmento R. A. Santos G. R. (2019). Botanical and synthetic pesticides alter the flower visitation rates of pollinator bees in neotropical melon fields. Environ. pollut. 251, 591–599. doi: 10.1016/j.envpol.2019.04.133 31108292

[B105] Valles S. M. Perera O. P. Strong C. A. (2003). Purification, biochemical characterization, and cDNA cloning of a glutathione S-transferase from the red imported fire ant, Solenopsis invicta. Insect Biochem. Mol. Biol. 33, 981–988. doi: 10.1016/s0965-1748(03)00104-8 14505691

[B106] Valles S. M. Perera O. P. Strong C. A. (2006). Gene structure and expression of the glutathione S‐transferase, SiGSTS1, from the red imported fire ant, Solenopsis invicta. Arch. Insect Biochem. Physiol. 61, 239–245. doi: 10.1002/arch.20116 16552769

[B107] Viana T. A. Barbosa W. F. Lourenço A. P. Santana W. C. Campos L. O. Martins G. F. (2021). Changes in innate immune response and detoxification in Melipona quadrifasciata (Apinae: Meliponini) on oral exposure to azadirachtin and spinosad. Apidologie 52, 252–261. doi: 10.1007/s13592-020-00814-w 30311153

[B108] Wanchoo A. Zhang W. Ortiz-Urquiza A. Boswell J. Xia Y. Keyhani N. O. (2020). Red imported fire ant (Solenopsis invicta) chemosensory proteins are expressed in tissue, developmental, and caste-specific patterns. Front. Physiol. 11, 585883. doi: 10.3389/fphys.2020.585883 33192598 PMC7646262

[B109] Wang G. Zhou H. Yu S. Wang Z. Zeng Y. Wu X. . (2024). Behavioral preferences of Solenopsis invicta Buren to essential oils and active compounds from amiaceae plants. Ind. Crop Prod. 214, 118471. doi: 10.1016/j.indcrop.2024.118471 38826717

[B110] Wei Z. Ortiz-Urquiza A. Keyhani N. O. (2021). Altered expression of chemosensory and odorant binding proteins in response to fungal infection in the red imported fire ant, Solenopsis invicta. Front. Physiol. 4, 596571. doi: 10.3389/fphys.2021.596571 33746766 PMC7970113

[B111] Wen C. Chen J. He Y. Wang F. Qian C. Wen J. . (2021). Electrophysiological and behavioral responses of red imported fire ants (Hymenoptera: Formicidae) to an essential balm and its components. Pest Manage. Sci. 77, 1971–1980. doi: 10.1002/ps.6225 33314506

[B112] Wen Y. Ma T. Chen X. Liu Z. Zhu C. Zhang Y. . (2016). Essential balm: a strong repellent against foraging and defending red imported fire ants (Hymenoptera: Formicidae). J. Econ. Entomol. 109, 1827–1833. doi: 10.1093/jee/tow130 27298425

[B113] Wicher D. Marion-Poll F. (2018). Function and regulation of chemoreceptors. Front. Cell. Neurosci. 12, 496. doi: 10.3389/fncel.2018.00496 30618642 PMC6305422

[B114] Wicher D. Miazzi F. (2021). Functional properties of insect olfactory receptors: ionotropic receptors and odorant receptors. Cell Tissue Res. 383, 7–19. doi: 10.1007/s00441-020-03363-x 33502604 PMC7873100

[B115] Wilson E. O. (1978). Division of labor in fire ants based on physical castes (Hymenoptera: Formicidae: Solenopsis). J. Kans. Entomol. Soc 51, 615–636.

[B116] Wu D. Zeng L. Lu Y. Xu Y. (2016). Effect of Solenopsis invicta (Hymenoptera: Formicidae) on flower-visiting behavior of insects on Brassica napus (Brassicales: Brassicaceae). Fla. Entomol. 99, 166–171. doi: 10.1653/024.099.0202

[B117] Wurm Y. Wang J. Riba-Grognuz O. Corona M. Nygaard S. Hunt B. G. . (2011). The genome of the fire ant Solenopsis invicta. Proc. Natl. Acad. Sci. U.S.A. 108, 5679–5684. doi: 10.1073/pnas.1009690108 21282665 PMC3078418

[B118] Wylie R. Yang C. C. S. Tsuji K. (2020). Invader at the gate: The status of red imported fire ant in Australia and Asia. Ecol. Res. 35, 6–16. doi: 10.1111/1440-1703.12076 40046247

[B119] Xiao C. X. Tan Y. T. Wang F. F. Wu Q. H. Qin D. Q. Zhang Z. X. (2020). The fumigating activity of Litsea cubeba oil and citral on Solenopsis invicta. Sociobiology 67, 41–47. doi: 10.13102/sociobiology.v67i1.4481

[B120] Xiao X. Zhao W. Shao Y. Hu C. Liu J. Zhang G. . (2024). Environmental exposure to cadmium induces olfactory neurotoxicity in fire ants and the molecular basis. Environ. pollut. 362, 124945. doi: 10.1016/j.envpol.2024.124945 39265771

[B121] Xie F. Rizvi S. A. H. Zeng X. (2020). Fumigant toxicity and biochemical properties of (α+ β) thujone and 1, 8-cineole derived from Seriphidium brevifolium volatile oil against the red imported fire ant Solenopsis invicta (Hymenoptera: Formicidae). Rev. Bras. Farmacogn. 29, 720–727. doi: 10.1016/j.bjp.2019.04.013 38826717

[B122] Xing H. Hu Y. Yang L. Lin J. Bai H. Li Y. . (2023a). Fumigation activity of essential oils of Cinnamomum loureirii toward red imported fire ant workers. J. Pest Sci. 96, 647–662. doi: 10.1007/s10340-022-01540-1 30311153

[B123] Xing H. Lin J. Li X. Huang J. Liang X. Li Y. . (2023b). Changes in dopamine and octopamine levels caused disordered behaviour in red imported fire ants exposed to cinnamon essential oils. Ind. Crop Prod. 199, 116801. doi: 10.1016/j.indcrop.2023.116801 38826717

[B124] Xu T. Chen L. (2021). Chemical communication in ant-hemipteran mutualism: Potential implications for ant invasions. Curr. Opin. Insect Sci. 45, 121–129. doi: 10.1016/j.cois.2021.04.004 33901733

[B125] Xu J. Lv M. Fang S. Wang Y. Wen H. Zhang S. . (2023b). Exploration of synergistic pesticidal activities, control effects and toxicology study of a monoterpene essential oil with two natural alkaloids. Toxins 15, 240. doi: 10.3390/toxins15040240 37104178 PMC10142011

[B126] Xu T. Xu M. Lu Y. Zhang W. Sun J. Zeng R. . (2021). A trail pheromone mediates the mutualism between ants and aphids. Curr. Biol. 31, 4738–4747. doi: 10.1016/j.cub.2021.08.032 34496221

[B127] Xu T. Zhang N. Xu M. Glauser G. Turlings T. C. Chen L. (2023a). Revisiting the trail pheromone components of the red imported fire ant, Solenopsis invicta Buren. Insect Sci. 30, 161–172. doi: 10.1111/1744-7917.13047 35451550

[B128] Yan H. Liebig J. (2021). Genetic basis of chemical communication in eusocial insects. Genes Dev. 35, 470–482. doi: 10.1101/gad.346965.120 33861721 PMC8015721

[B129] Yang F. Shao R. Zhao J. Li L. Wang M. Zhou A. (2021). Cadmium exposure disrupts the olfactory sensitivity of fire ants to semiochemicals. Environ. pollut. 287, 117359. doi: 10.1016/j.envpol.2021.117359 34020258

[B130] Yang F. Zhang G. Liu J. Duan S. Li L. Lu Y. . (2022). Sublethal exposure to cadmium induces chemosensory dysfunction in fire ants. Environ. Sci. Technol. 56, 12440–12451. doi: 10.1021/acs.est.2c03108 35944015 PMC9454817

[B131] Yasudai R. Matsubara A. Hsu P. W. Lee C. C. Lin C. C. Yang C. C. S. (2022). Laboratory and field evaluations of two bait formulations against the invasive fire ant, Solenopsis invicta (Hymenoptera: Formicidae). J. Econ. Entomol. 115, 624–630. doi: 10.1093/jee/toab255 35022766

[B132] Youssef D. A. A. Abdelmegeed S. M. (2021). Polymer-based encapsulation of peppermint oil (Mentha piperita) nanoemulsion and its effects on life and some physiological activities of honeybees Apis mellifera (Hymenoptera: Apidae). Egypt. Pharm. J. 20, 313–322. doi: 10.4103/epj.epj_49_21 41918118

[B133] Yu X. M. Luo X. Wang J. Wang L. H. Yu S. R. (2018). Cytochrome P450 4X1: Structure, distribution, regulation and function. Chin. J. Hosp. Pharm. 38, 1985–1988.

[B134] Zeng H. (2023). Functional properties of ant queen pheromones as revealed by behavioral experiments. Behav. Ecol. Sociobiol. 77, 113. doi: 10.1007/s00265-023-03378-8 30311153

[B135] Zeng H. Millar J. G. Chen L. Keller L. Ross K. G. (2022). Characterization of queen supergene pheromone in the red imported fire ant using worker discrimination assays. J. Chem. Ecol. 48, 109–120. doi: 10.1007/s10886-021-01336-0 34850312

[B136] Zhang W. Chen X. Tian J. Schal C. Mohamed A. Zang L. S. . (2025). An odorant-binding protein functions in fire ant social immunity interfacing with innate immunity. Open Biol. 15, 240254. doi: 10.1098/rsob.240254 39933575 PMC11813584

[B137] Zhang Y. Fu J. Huang C. Cheng D. Huang R. Zhang Z. X. (2017). Insecticidal activity of the soil in the rhizosphere of Viburnum odoratissimum against Solenopsis invicta (Hymenoptera: Formicidae). Sociobiology 64, 1–6. doi: 10.13102/sociobiology.v64i1.1067

[B138] Zhang G. Fu Y. Shao Y. Zhao J. Lei X. Fu Y. . (2023a). Semiochemicals produced by microbes in mealybug honeydew attract fire ants. J. Agric. Food. Chem. 71, 15456–15465. doi: 10.1021/acs.jafc.3c04444 37843466

[B139] Zhang N. Liao Y. Xie L. Zhang Z. Hu W. (2021). Using essential oils from Citrus paradisi as a fumigant for Solenopsis invicta workers and evaluating the oils’ effect on worker behavior. Environ. Sci. pollut. Res. 28, 59665–59672. doi: 10.1007/s11356-021-14910-6 34142322

[B140] Zhang W. Wanchoo A. Ortiz-Urquiza A. Xia Y. Keyhani N. O. (2016). Tissue, developmental, and caste-specific expression of odorant binding proteins in a eusocial insect, the red imported fire ant, Solenopsis invicta. Sci. Rep. 6, 35452. doi: 10.1038/srep35452 27765943 PMC5073229

[B141] Zhang B. Yang R. R. Jiang X. C. Xu X. X. Wang B. Wang G. R. (2023b). Genome-wide analysis of the odorant receptor gene family in Solenopsis invicta, Ooceraea biroi, and Monomorium pharaonis (Hymenoptera: Formicidae). Int. J. Mol. Sci. 24, 6624. doi: 10.3390/ijms24076624 37047591 PMC10095046

[B142] Zhao K. Wu H. Hou R. Wu J. Wang Y. Huang S. . (2022). Effects of sublethal azadirachtin on the immune response and midgut microbiome of Apis cerana cerana (Hymenoptera: Apidae). Ecotoxicol. Environ. Saf. 229, 113089. doi: 10.1016/j.ecoenv.2021.113089 34929506

[B143] Zhou W. Li M. Achal V. (2024). A comprehensive review on environmental and human health impacts of chemical pesticide usage. Emerging Contam. 11, 100410. doi: 10.1016/j.emcon.2024.100410 38826717

