# Supplementary material for: Plant derived biopesticides for controlling imported fire ants (Solenopsis spp.): a comprehensive review on biochemical traits, promising alternatives, and mechanisms of action
Source: Front Plant Sci. 2026 Jul 6;17:1848932. doi: 10.3389/fpls.2026.1848932 (PMC13381485; doi:10.3389/fpls.2026.1848932)
Supplement: Supplementary file 1 [file DataSheet1.docx]

Supplementary Material

Plant derived biopesticides for controlling imported fire ants (*Solenopsis* spp.): a comprehensive review on biochemical traits, promising alternatives, and mechanisms of action

Maricruz Rangel-Galván^1,2^, Yesenia Ithaí Ángeles-López^1^, Nemesio Villa-Ruano^1*^

^1^SECIHTI-Dirección de Innovación y Transferencia de Conocimiento, Benemérita Universidad Autónoma de Puebla, Puebla, México

^2^Facultad de Ciencias Biológicas, Benemérita Universidad Autónoma de Puebla, Puebla, México

# Supplementary Data index

**Table S1** Corresponding bioactive compounds in biopesticide plants against *Solenopsis* spp

**Table S2** Biocidal compounds with mortality and repellent effects on *Solenopsis* spp.

**Table S3** Mortality effect of biopesticides based on plants against *Solenopsis* spp.

**Table S4** Repellent properties of plant-derived biopesticides against *Solenopsis* spp**.**

**Table S5** Biopesticides based on plants as inhibitors of AChE enzyme

**Table S6** Biopesticides based on plants as inhibitors of GST enzyme

**Table S1** Corresponding bioactive compounds in biopesticide plants against *Solenopsis* spp

| **Num** | **Plant** | **Concentration of major compound** | **Reference** |
| --- | --- | --- | --- |
| 1 | *Piper aduncum* | Dillapiole (64.4%) | Souto *et al.*, 2012 |
| 2 | *Piper marginatum* (chemotypes A) | P-Mentha-1(7), 8-diene (39.0%), 3,4-methylenedioxypropiophenone (19.0%), and (E)-β-ocimene (9.8%) | Souto *et al.*, 2012 |
| 3 | *Piper marginatum* (chemotypes B) | (E)-Isoosmorhizole (32.2%), (E)-anethole (26.4%), isoosmorhizole (11.2%), (Z)-anethole (6.0%) | Souto *et al.*, 2012 |
| 4 | *Piper divaricatum* | Methyleugenol (69.2%), eugenol (16.2%) | Souto *et al.*, 2012 |
| 5 | *Piper callosum* | Safrole (69.2%), methyleugenol (8.6%), β-pinene (6.2%). | Souto *et al.*, 2012 |
| 6 | *Hedychium coccineum* | Linalool (27%), α-pinene (14%) | Sakhanokho *et al.*, 2013 |
| 7 | *Hedychium flavum* | 1,8-Cineole (28.3%), α-pinene (12.1%) | Sakhanokho *et al.*, 2013 |
| 8 | *Hedychium flavescens* | Linalool (35%), β-pinene (27%), 1,8-cineole (13%). | Sakhanokho *et al.*, 2013 |
| 9 | *Hedychium oils* | 1,8-Cineole (0.1%–42%), linalool (<0.1%–56%), α-pinene (3%–17%), β-pinene (4%–31%), (E)-nerolidol (0.1%–20%). | Sakhanokho *et al.*, 2013 |
| 10 | Cinnamon's younger green leaves | Cinnamic aldehyde (0.2%), eugenol (1.3%) | Huang *et al.*, 2015 |
| 11 | Cinnamon's fallen yellow green leaves | Cinnamic aldehyde (0.5%), eugenol (4.3%) | Huang *et al.*, 2015 |
| 12 | Cinnamon's fallen brown leaves | Cinnamic aldehyde (0.2%), eugenol (1.7% ) | Huang *et al*., 2015 |
| 13 | *Murraya exotica* (fresh leaves) | β-Caryophyllene (40.20%), (+)-b-himachalene (17.76%), linalool (8.81%) | Zhang *et al.*, 2016 |
| 14 | *Murraya exotica* (fallen leaves) | β-Caryophyllene (25.30%), α-cedrene (14.01%), curcumene (10.56%) | Zhang *et al.*, 2016 |
| 15 | *Murraya exotica* (dried leaves) | α-Cedrene (25.05%), β-caryophyllene (23.43%), germacrene D (9.13%) | Zhang *et al*., 2016 |
| 16 | Esencial balm | Menthol 48.0%, methyl salicylate 27.4%, eucalyptol 9.9%, D-(+)-camphor 7.3%, eugenol 4.2%, phenylethyl alcohol 1.9%, dipropylene glycol 1.3% | Wen *et al*., 2016 |
| 17 | *Cupressus nootkatensis* | Nootkatene (57%), valencene (15%) | Adesso *et al.*, 2017 |
| 18 | *Viburnum odoratissimum* (soil) | Methyl salicylate: 0.01-5 mg/L standard; fresh soil: 0.13 -4.88 mg/kg; dry soil: 0.16 to 5.97 mg/kg; in the soil at depths 0-5 cm: 4.88 mg/kg, 5-10cm: ~ 2mg/kg,10-15cm: ~0.5mg/kg, 15-20cm: ~0.1mg/kg. | Zhang *et al*., 2017 |
| 19 | *Viburnum odoratissimum* (leaves) | Methyl salicylate: fresh leaves: 133.3, 150.8, and 106.3 mg/kg; dry leaves: 486.6, 401.2, and 193.9 mg/kg. Fresh yellow leaves: 106.3 mg/kg; dry yellow leaves: 193.9 mg/kg | Zhang *et al.*, 2017 |
| 20 | *Michelia alba* (Fresh leaves) | Linalool 26.10%, like isocaryophyllene, aromadendrene, α-caryophyllene, and (-)-γ-cadinene account for more than 40% | Qin *et al.*, 2018 |
| 21 | *Michelia alba* (Fallen leaves) | Linalool 40.52%, β-elemene (11.94%), β-caryophyllene (10.78%) | Qin *et al.*, 2018 |
| 22 | *Michelia alba* Leaves (Dried leaves) | Linalool 36.52%, β-elemene, β-caryophyllene, α-selinene, α-cubebene ~33% | Qin *et al.*, 2018 |
| 23 | *Artemisia annua* | Camphor (32 %), germacrene D (21 %) | Seixas *et al.*, 2018 |
| 24 | *Artemisia absinthium* | Z-Isocitral (22 %), myrcene(18 %), β-pinene (15 %) | Seixas *et al.*, 2018 |
| 25 | *Artemisia camphorata* | Germacrene D-4-ol (22 %), 1,8-cineole (12 %), ascaridole(10 %), borneol (10 %) | Seixas *et al.*, 2018 |
| 26 | *Artemisia vulgaris* | Methyleugenol (57 %), β-thujone (31 %), β-pinene (26 %), 1,8-cineole (19 %) | Seixas *et al.*, 2018 |
| 27 | *Eutrema japonicum* (Miq.) Kiudz | Allyl isothiocyanate | Hashimoto *et al.*, 2019 |
| 28 | *Seriphidium brevifolium* | 2-Bornanone (28.2%), 1,8-cineole (19.9%), α-thujone (7.5%), β-thujone (6.7%) | Xie *et al.*, 2020 |
| 29 | Brassicaceae | Allyl isothiocyanate, 3-butenyl isothiocyanate, 3-(methylthio) propyl isothiocyanate, and 2-phenylethyl isothiocyanate | Du *et al.*, 2020 |
| 30 | *Colchicum autumnale* L. | Colchicine | Lin *et al.*, 2020 |
| 31 | *Litsea cubeba* | Citral (27.76%) and (z)-3,7-dimethylocta-2,6-diena (26.13%) | Xiao *et al.*, 2020 |
| 32 | *Gelsemium elegans* | Koumine | Zheng *et al.*, 2020 |
| 33 | *Citrus paradisi* (crude grapefruit oil (GO1)) | D-Limonene (70.1%), β-pinene (5.09 %), α-pinene (2.05 %), β-phellandrene (1.93%), octanal (0.88 %), d-carvone (0.52 %), α-terpineol (0.52 %), linalool (0.55 %) | Zhang *et al.*, 2021 |
| 34 | *Citrus paradisi* (concentrated grapefruit oil (GO2)) | D-Limonene (73.96%), β-pinene (5.85%), α-pinene (2.74%), β-phellandrene (2.22%), octanal (1.02%), d-carvone (0.63%), α-terpineol (0.54%), linalool (0.59%) | Zhang *et al.*, 2021 |
| 35 | *Magnolia grandiflora* L. (seed essential oil) | Aliphatic esters (10.9%), 1-decanol (3.3%), 1-octanol (6.2%), and 1-heptanol (0.5%) | Ali *et al.*, 2022 |
| 36 | *Osmanthus fragrans* (Thunb.) Lour. | Butyl phthalate (37.95 %), p-methoxyphenethyl alcohol (16.76 %) | Li *et al.*, 2022 |
| 37 | *Sophora japonica* Linn | Benzeneethanol (20.62 %), phenol (11.16 %) | Li *et al.*, 2022 |
| 38 | *Eugenia caryophμllata* Thunb. | Eugenol (82.5 %), aceteugenol (15.81 %) | Li *et al.*, 2022 |
| 39 | *Ligustrum compactum* (Wall. exG. Don) Hook.f. | Benzyl alcohol (17.45 %), phenethyl alcohol (27.63 %) | Li *et al.*, 2022 |
| 40 | *Jasminum sambac* (L.) Ait. | Benzyl alcohol (19.33 %), linalool (18.70 %) | Li *et al.*, 2022 |
| 41 | *A. polyrhizum* | Pentacosane (11.78 %), heptacosane (10.37 %), oleylamide (11.9 %), non-acosane (20.15 %), octacosane (8.24 %) | Li *et al.*, 2022 |
| 42 | *Curcuma longa* (leaf) | *Ar*-Curcumene 20.49%, Ar-turmerone 42.39% | Ali *et al.*, 2024 |
| 43 | *Curcuma longa* (rhizome) | *A*r-Turmerone 40.35%, tumerone 23.73 %, curlone 5.81%, *Ar*-curcumene 5.01% | Ali *et al.*, 2024 |
| 44 | *Baccharis microdonta* | Kongol (22.22%), spathulenol (22.74%) | Ali *et al.*, 2023 |
| 45 | *Baccharis pauciflosculosa* | Limonene (18.77%), β-pinene (18.33%) | Ali *et al.*, 2023 |
| 46 | *Baccharis punctulata* | α-Bisabolol (23.63%) | Ali *et al.*, 2023 |
| 47 | *Baccharis reticularioides* | α-Pinene (24.50%) | Ali *et al.*, 2023 |
| 48 | *Baccharis sphenophylla* | α-Pinene (10.74%); spathulenol (13.15%), limonene (14.33%), β-pinene (15.24%) | Ali *et al.*, 2023 |
| 49 | *Lippia gracilis* | Carvacrol (50.7%) | Dantas *et al.*, 2023 |
| 50 | *Illicium verum* | Acetaldehyde (15.47 %), acetic acid, methyl ester (10.44%), benzene, 1-methoxy-4-(1-propenyl)- (16.59%), D-limonene (5.87%) | Fu *et al.*, 2023 |
| 51 | *Blumea balsamifera* | Ethanol (10.32%), (+)-2-bornanone (15.6%), D-limonene (0.09%*); Citrus limon:* β-pinene (19.61%), D-limonene (48.8%) | Fu *et al.*, 2023 |
| 52 | *Citrus limon* | β-Pinene (19.61%), D-limonene (48.8%) | Fu *et al.*, 2023 |
| 53 | *Acorus tatarinowii* | Acetone (15.47 %), 3-cyclohexene-1-methanol, 4-trimethyl-, acetate (30.43%), D-limonene (6.18%) | Fu *et al.*, 2023 |
| 54 | *Mosla chinensis* | D-Limonene (15.46%) | Fu *et al.*, 2023 |
| 55 | *Cinnamomum cassia* | D-Limonene (10.48%), eucalyptol (16.47%) | Fu *et al.*, 2023 |
| 56 | *Houttuynia cordata* Thunb | 2-Undecanone | Kurmanbayeva *et al.*, 2023 |
| 57 | *Celastrus angulatus* | Azadirachtin, celangulin, vetramine | Liang *et al.*, 2023b |
| 58 | *Matricaria chamomilla* (flower essential oils (EOs) blue Egyptian (EO-1)) | α-Bisabolol oxide A (49.49 %), (E)-β-farnesene (13.98%) | Shah *et al.*, 2023 |
| 59 | *Matricaria chamomilla* (flower essential oils (EOs) chamomile German CO2 (EO-2)) | α-Bisabolol (81.85%) | Shah *et al.*, 2023 |
| 60 | *Matricaria chamomilla* (flower essential oils (EOs) chamomile German (EO-3) | α-Bisabolol oxide A (27.83 %), (E)-β-farnesene (16.56 %) | Shah *et al.*, 2023 |
| 61 | Garlic | Diallyl disulfide (46.51%), (E)-1-allyl-2-(prop-1-en-1-yl) disulfane (34.68%) | Song *et al.*, 2023 |
| 62 | *Sophora flavencens* | Matrine and sophocarpine | Tian *et al.*, 2023 |
| 63 | *Cinnamomum loureirii* | *Trans*-Cinnamaldehyde (bark oil: 823.08 mg/g ± 7.39; leaf oil: 549.46 mg/g ± 9.38) | Xing *et al.*, 2023a |
| 64 | *Cinnamomum cassia* | *Trans*-Cinnamaldehyde (bark oil: 525.96 mg/g ± 12.71; leaf oil: 482.57 mg/g ± 1.19). | Xing *et al.*, 2023a |
| 65 | *Cinnamomum loureirii* | *Trans*-Cinnamaldehyde 63.20%, cinnamyl acetate 27.52%; (bark essential oils 803.28 mg/g) (leaf: 532.85 mg/g) | Xing *et al.*, 2023b |
| 66 | *Cinnamomum cassia* | *Trans*-Cinnamaldehyde in bark: 531.25 mg/g, in leaf: 499.52 mg/g) | Xing *et al.*, 2023b |
| 67 | *Artemisia argyi* Levl. et Vant. | Dipentene (24.04 ± 0.53%), 1-caryophyllene (21.18 ± 0.42%), p-cymene (7.97 ± 0.17%), α-terpineol (7.57 ± 0.06%), Δ-cadinene (4.28 ± 0.13 %%) | Fan *et al.*, 2024 |
| 68 | *Santalum album* L. | Diethyl phthalate (11.56 ± 0.21%), α-cetone (8.23 ± 0.21%), 2-phenylethanol (8.19 ± 0.50%), sandacanol (5.32 ± 0.29%) | Fan *et al.*, 2024 |
| 69 | *Salvia miltiorrhiza* (active compounds) | Phenylethyl alcohol, *cis*-anethole, thexyl alcohol, β-ionol, hexahydrofarnesyl acetone in minor workers, 2-ethyl-3,5-dimethyl-pyrazine, phenylethyl alcohol, isoobtusilactone in major workers; phenylethyl alcohol. 4-ethyl-benzaldehyde, thexyl alcohol, *trans*-anethole for male ants; borneol for virgin queens | Wang *et al.*, 2024 |
| 70 | *Perilla frutescens* | Diketone alcohol and patchouli alcohol in minor worker ants; eugenol and 3-methylcyclo pentanol in major workers; isocaproic acid for male ants; 3-methylcyclopentanol, 2-phenylpropane, benzyl alcohol, phenylethyl alcohol, isoegomaketone, perilla ketone, eugenol, phytol for virgin queens | Wang *et al.*, 2024 |
| 71 | *Pogostemon cablin* | α-Bulnesene for minor worker ants; isoaromadendrene epoxide and pogostol for larger ants; caryophyllene oxide, pogostol for males; caryophyllene, α-guaiene, seychellene for virgin queens | Wang *et al.*, 2024 |
| 72 | *Schizonepeta tenusfolia* | *S. tenusfolia*: ethenyl-benzene, benzaldehyde, *cis*-carveol, pulegone, widdrol for minor workers; ethenyl-benzene, menthofuran, 2-cyclopentylidenecyclopentanone, dodecane, pulegone, mintlactone, 7a-hydroxymintlactone, 13-methylpentadecanoate methyl for major workers; ethenyl-benzene, benzaldehyde, isopulegone, 2-cyclopentylidene cyclopentanone, dodecane for male ants; pulegone for virgin queens | Wang *et al.*, 2024 |
| 73 | *Mentha canadensis* | 3-Octanol, benzyl alcohol, menthone, menthol, caryophyllene oxide, tofisopam for minor workers; linalool, pulegone, and dehydrodiisoeugenol for major workers | Wang *et al.*, 2024 |
| 74 | 20 commercial EOs including: *Mentha spicata, Acorus calamus, Pinus massoniana, Mentha haplocalyx, Nepeta cataria, Artemisia argyi, Eugenia Caryophyllus, Forsythia suspensa, Cymbopogon citratus, Ruta graveolens, Lavandula angustifolia, Artemisia annua, Angelica sinensis, Pogostemon cablin, Melaleuca alternifolia, Prunus amygdalus, Dendranthema indicum, Cinnamomum camphora, Eucalyptus globulus, and Cinnamomum cassia.* | (+)- 2-Bornanone,.beta.-pinene, eucalyptol, isoborneol, linalool, L-.alpha.-terpineol | Zhou *et al.*, 2024a |
| 75 | *Artemisia* subg. *Seriphidium* | 1,8-Cineole, camphor, α-thujone, β-thujone, citral, geraniol | Rizvi *et al.*, 2025 |

**Table S2** Biocidal compounds with mortality and repellent effects on *Solenopsis* spp.

| **Compound** | **Chemical Family** | **Structure** | **References** |
| --- | --- | --- | --- |
| Azadirachtin | Limonoid |  | Liang *et al.*, 2023b |
| Celangulin | Sesquiterpene |  | Liang *et al.*, 2023b |
| d-Limonene | Monoterpene |  | Zhang *et al.*, 2021 |
| Octanal | Aldehyde |  | Zhang *et al.*, 2021 |
| α-Pinene | Monoterpene |  | Zhang *et al.*, 2021 |
| β-Phellandrene | Monoterpene |  | Zhang *et al.*, 2021 |
| d-Carvone | Monoterpene (Ketone) |  | Zhang *et al.*, 2021 |
| α-Terpineol | Monoterpene (Alcohol) |  | Zhang *et al.*, 2021 |
| Allyl isothiocyanate | Isothiocyanate |  | Du *et al.*, 2020 |
| 2-Phenylethyl isothiocyanate | Isothiocyanate |  | Du *et al.*, 2020 |
| 3-(Methylthio) propyl isothiocyanate | Isothiocyanate |  | Dantas *et al.*, 2023 |
| Carvacrol | Monoterpene (Phenol) |  | Dantas *et al.*, 2023 |
| Carvacryl benzoate | Ester of phenol Monoterpene |  | Dantas *et al.*, 2023 |
| Carvacryl pivaloate | Monoterpene |  | Dantas *et al.*, 2023 |
| Diallyl disulfide | Organosulfur (Disulfide) |  | Song *et al*., 2023 |
| Methyl allyl disulfide | Organosulfur (Disulfide) |  | Song *et al*., 2023 |
| Citral | Aldehyde Terpenoid |  | Xiao *et al.*, 2020 |
| 1,8-Cineole | Ether Monoterpene |  | Rizvi *et al.*, 2025 |
| Camphor | Monoterpene (Ketone) |  | Rizvi *et al.*, 2025 |
| α-Thujone | Monoterpene (Ketone) |  | Rizvi *et al.*, 2025 |
| β-Thujone | Monoterpene (Ketone) |  | Rizvi *et al.*, 2025 |
| Geraniol | Terpenoid (Alcohol) |  | Rizvi *et al.*, 2025 |
| 1-Decanol | Alcohol (Aliphatic) |  | Ali *et al.*, 2022 |
| 1-Octanol | Alcohol (Aliphatic) |  | Ali *et al.*, 2022 |
| 2-Undecanone | Ketone |  | Ali *et al.*, 2022 |
| α-Bisabolol | Sesquiterpene (Alcohol) |  | Shah *et al.*, 2023 |
| *Trans*-Cinnamaldehyde | Aromatic (Aldehyde) |  | Xing *et al.*, 2023a |
| Cinnamyl acetate | Aromatic (Ester) |  | Xing *et al.*, 2023a |
| Eugenol | Phenylpropanoid (Phenol) |  | He *et al.*, 2023 |
| Isoeugenol | Phenylpropanoid (Phenol) |  | He *et al.*, 2023 |
| Methyl isoeugenol | Ester of Phenylpropanoid (Phenol) |  | He *et al.*, 2023 |
| Eugenol acetate | Ester of Phenylpropanoid (Phenol) |  | He *et al.*, 2023 |
| Methyl eugenol | Phenylpropanoid (Phenol) |  | He *et al.*, 2023 |
| Matrine | Alkaloid |  | Tian *et al.*, 2023 |
| Sophocarpine | Alkaloid |  | Tian *et al.*, 2023 |
| Koumine | Alkaloid |  | Zheng *et al.*, 2020 |
| Colchicine | Alkaloid |  | Lin *et al.*, 2020 |
| Methyl salicylate | Salicylate (Ester) |  | Wen *et al.*, 2016 |
| Cinnamic aldehyde | Aromatic (Aldehyde) |  | Huang *et al.*, 2015 |
| Linalool | Monoterpene (Alcohol) |  | Qin *et al*., 2018 |

**Table S3** Mortality effect of biopesticides based on plants against *Solenopsis* spp.

| **Plant** | **Target** | **Mortality Effect** | **Reference** |
| --- | --- | --- | --- |
| *Piperaceae* spp. | *Solenopsis saevissima* (Smith) | **500 mg/L (48 h):**   1. *P. aduncum*: 100% 2. *P. marginatum* chemotype A: 100% 3. *P. marginatum* chemotype B: 100% 4. *P. divaricatum*: 79.8% 5. *P. callosum:* 70.6%. | Souto *et al.,* 2012 |
| *Cinnamomum camphora* (L. J.) Presl, *Artemisia annua* (L.), *Eucalyptus globulus* (Labill.), *Artemisia argyi* (H. Lév. and Vaniot), *Ilex chinensis* (Sims), *Dendranthema indicum* (L. Des Moul), *Pinus massoniana* (Lamb), *Forsythia suspense* (Thunb. Vahl) | *Solenopsis invicta* | **2 mg/centrifuge tube**:   1. *C. camphora*, *A. annua*, *E. globulus* oils: 100% **(**minor workers) 2. *I. chinensis*, *A. argyi* , *P.massoniana*, *F. suspense* oils: >80% (minor workers). 3. *C. camphora, E. globulus* oils: >80% (major workers)   **5 mg/centrifuge tube** (12 h):   1. *C. camphora*, *A. annua*, *E. globulus*, *A. argyi*, *P.massoniana* oils: 100% (minor/major workers) | Tang *et al.,* 2013 |
| *Cinnamomum aromaticum* Nees | *Solenopsis invicta* | **Major workers after 5 days of treatment:**   1. 64.0% (0 - 5 cm) 2. 88.0% (5 - 10 cm) 3. 80.0% (10 - 15 cm) 4. 72.0% (15 - 20 cm)   **Minor workers after 5 days of treatment:**   1. 75.0% (0 - 5 cm) 2. 100.0% (5 - 10 cm) 3. 91.7% (10 - 15 cm) 4. 87.5% (15 - 20 cm) | Huang *et al.,* 2015 |
| *Murraya exotica* | *Solenopsis invicta* | **Major workers after 9 days of treatment:**   1. 93.33% (fresh leaves) 2. 83.33% (fallen leaves) 3. 83.33% (dried leaves)   **Minor workers after 9 days of treatment:**   1. 100.00% (fresh leaves) 2. 93.33% (fallen leaves) 3. 83.33% (dried leaves) | Zhang *et al.,* 2016 |
| *Cupressus nootkatensis* | *Solenopsis invicta* (Buren); *Solenopsis richteri* (Forel) | **24-h Exposure:**   1. 84% with 2% nootka oil   **48-h Exposure:**   1. 100 % with 100 µl nootka oil vapor treatment | Addesso *et al.,* 2017 |
| *Viburnum odoratissimum* | *Solenopsis invicta* | **Major ants after 12 days of treatment**   1. 100% (soil depth 0-5 cm) 2. 68.75% (soil depth 5-10 cm)   **Minor ants after 11 days of treatment**   1. 100% (soil depth 0-5 cm) 2. 65% (soil depth 5-10 cm) | Zhang *et al.,* 2017 |
| *Michelia alba* (leaves) | *Solenopsis invicta* | **Major ants after 64 h of treatment**   1. 100.0% (fallen leaves) 2. 46.67% (fresh leaves) 3. 20.00% (dried leaves)   **Minor ants after 64 h of treatment**   1. 100.0% (fallen leaves) 2. 81.67% (fresh leaves) 3. 11.67% (dried leaves) | Qin *et al.,* 2018 |
| *Artemisia annua* | *Solenopsis saevissima* (Smith) | 51.54 µg/mg caused 42% of mortality | Seixas *et al.,* 2018 |
| *Seriphidium brevifolium* | *Solenopsis invicta* | **Volatile oil** **at 20 µl/L**   1. 94.95 % (18 h) 2. 100 % (24 h)   **(α+ β) Thujone** **at 28 µl/L**   1. 92.27 % (18 h) 2. 100 % (24 h)   **1,8-Cineole at 80 µl/l**   1. 94.47 % (18 h) 2. 100 % (24 h) | Xie *et al.,* 2020 |
|  | *Solenopsis geminata* | **100% Mortality**   1. Clove powder 81mg (120 min) 2. Cinnamon powder 108 mg (240 min) 3. Coriander powder 108 mg (240 min) 4. Clove oil 2-2.5% (30 min) 5. Peppermint oil 1.5, 2, and 2.5% (60 min) 6. Eucalyptus oil 2-2.5% (120 min) | Nalini *et al.,* 2020 |
| *Colchicum autumnale* L. | *Solenopsis invicta* | **3-Day treatment:**   1. Bulb powder 54.67% sugar water/5000 mg/L 2. Colchicine 45.33% sugar water/50 mg/L | Lin *et al.,* 2020 |
| *Litsea cubeba* | *Solenopsis invicta* | **24 h of treatment (5µL/cm3)**   1. *L.cubeba* oil: 60% (major) - 90% (minor) 2. Citral: 76% (major) - 96% (minor) | Xiao *et al.,* 2020 |
| *Gelsemium elegans* | *Solenopsis invicta* | **10th Day treatment:**   1. *G. elegans* extract: 55.00% (1/20 leaf extract) 2. *G. elegans* extract: 46.67% (1/20 stem extract) 3. Koumine: 45.00% (1 mg/kg) | Zheng *et al.,* 2020 |
| *Citrus paradisi* | *Solenopsis invicta* | **5µL/tube:**   1. GO2: 86% mortality (5 h); 100% (7 h) 2. GO1: >78% mortality (9 h); 100% (10 h) 3. Octanal: 88.33% (4 h) 4. Octanal:100% (6 h) 5. α-Pinene: 100% (6 h) 6. β-Phellandrene: 91.67% (6 h); 100% (8 h) 7. d-Carvone: 88.33% (6 h); 100% (8 h) 8. α-Terpineol: 81.67% (6 h): 100% (8 h) | Zhang *et al.,* 2021 |
| *Illicium verum, Blumea balsamifera, Citrus limon, Acorus tatarinowii, Mosla chinensis, Cinnamomum cassia* | *Solenopsis invicta* | **0.01 µL/cm^3^:**   1. *Citrus limon*: 60% (12 h); 100% (12 d) 2. *Acorus tatarinowii*: 100% (12 h) 3. *Mosla chinensis*: 100% (12 h) 4. *Cinnamomum cassia*: 100% (12 h) 5. *Blumea balsamifera*: 100% (12 h) 6. *Illicium verum*: 100% (12 h)   **0.001 µL/cm^3^:**   1. *Citrus limon*: 20% (12 d) 2. *Acorus tatarinowii*: 100% (12 d) 3. *Mosla chinensis*: 68% (12 d) 4. *Cinnamomum cassia*: 90% (12 d) 5. *Blumea balsamifera*: 100% (12 d) 6. *Illicium verum*: 90% (12 d) | Fu *et al.,* 2023 |
|  | *Solenopsis invicta* | **After 72 h of treatment:**   1. Methyl isoeugenol (0.5,0.15,0.05, and 0.015 mg/cm^2^) 2. Isoeugenol (0.5, 0.15, 0.05, and 0.015 mg/cm^2^) 3. Eugenol acetate (0.5, 0.15, 0.05, and 0.015 mg/cm^2^) 4. Methyl eugenol (0.5, 0.15, and 0.05 mg/cm^2^) 5. Eugenol (0.5, 0.15, and 0.05 mg/cm^2^). 6. Isoeugenyl acetate (None of the concentrations caused 100% mortality) | He *et al.,* 2023 |
| *Matricaria chamomilla* | *Solenopsis invicta (RIFA), Solenopsis richteri (BIFA), Hybrids (HIFA)* | **250 µg/g against HIFA:**   1. EO-1 73% 2. EO-2 20% 3. EO-3 40% 4. α-bisabolol 80% | Shah *et al.,* 2023 |
| *Allium sativum* | *Solenopsis invicta* | **Major ants after 12 h of treatment**   1. 63.33 % (4 µg/ml) 2. 91.67 % (16 µg/ml)   **Minor ants after 12 h of treatment**   1. 68.33% (4 µg/ml) 2. 100.0 % (16 µg/ml) | Song *et al.,* 2023 |
| *Cinnamomum loureirii* | *Solenopsis invicta* | **320 µg/cm^3^:**   1. *C. loureirii* leaf: 100% (1 h) 2. *C. loureirii* bark: 100% (1 h)   **160 µg/cm^3^:**   1. *C. loureirii* leaf: 100% (10 h) 2. *C. loureirii* bark: 96.67% (10 h) 3. **µg /cm^3^:** 4. *Trans*-Cinnamaldehyde: 100% (20 h)   Note: 2:1 mixture of 75 µg/cm^3^ (50µg/cm^3^ trans-cinnamaldehyde + 25 µg/cm^3^ cinnamyl acetate produced 90% mortality) | Xing *et al.,* 2023a |
| 20 commercial EOs including: *Mentha spicata, Acorus calamus, Pinus massoniana, Mentha haplocalyx, Nepeta cataria, Artemisia argyi, Eugenia Caryophyllus, Forsythia suspensa, Cymbopogon citratus, Ruta graveolens, Lavandula angustifolia, Artemisia annua, Angelica sinensis, Pogostemon cablin, Melaleuca alternifolia, Prunus amygdalus, Dendranthema indicum, Cinnamomum camphora, Eucalyptus globulus, and Cinnamomum cassia.* | *Solenopsis invicta* | **5 mg/Centrifuge tube (24 h) for minor ants:**   1. *Pinus massoniana* 97.8% 2. *Mentha haplocalyx* 86.7% 3. *Nepeta cataria* 95.6% 4. *Artemisia argyi* 100% 5. *Forsythia suspensa* 100% 6. *Cymbopogon citratus* 86.7% 7. *Artemisia annua* 97.8% 8. *Dendranthema indicum* 100% 9. *Cinnamomum camphora* 100% 10. *Eucalyptus globulus* 100%   **5 mg/Centrifuge tube (24 h) for major ants:**   1. *Pinus massoniana* 100% 2. *Mentha haplocalyx* 73.3% 3. *Nepeta cataria* 20.0% 4. *Artemisia argyi* 97.8% 5. *Forsythia suspensa* 100% 6. *Cymbopogon citratus* 66.7% 7. *Artemisia annua* 100% 8. *Dendranthema indicum* 100% 9. *Cinnamomum camphora* 100% 10. *Eucalyptus globulus* 10%   **5 mg/Centrifuge tube (24 h) for molecules:**   1. (+)- 2-Bornanone, beta-pinene (100% minor/100% major) 2. Eucalyptol (100% minor/100% major) 3. Isoborneol (82.2% minor/15.6% major) 4. Linalool (100% minor/20% major) 5. L-Alpha.-terpineol (100% minor/24.4% major) | Zhou *et al.,* 2024 |
| *Cnidium monnieri, Melia toosendan, Tripterygium wilfordii* | *Solenopsis invicta* | **50 mg/mL (2 days post-treatment):**   1. *T. wilfordii* exhibited 100 % mortality 2. *C. monnieri* exhibited 90.67% mortality 3. *M. toosendan* exhibited 90.67% mortality 4. *M. toosendan* exhibited 100% mortality | Guo *et al.,* 2025 |
| *S. brevifolium, S. kaschgaricum, S. badghysum, S. terrae-albae, S. kurramense, S. mongolorum, S. balchanorum, S. gracilescens, S. schrenkianum*, and *S. tauricum*. | *Solenopsis invicta* | **4μL/L (120 min) for minor and major workers:**   1. *S. brevifolium, S. kaschgaricum, S. terrae-albae, and S. kurramense* achieved 100% mortality 2. *S. mongolorum, S. schren kianum, S. tauricum, and S. gracilescens* achieved 90–97% mortality 3. 1,8-Cineole, (-)-α-thujone, (α+β)-thujone, D (+)-camphor achieved 100% mortality. | Rizvi *et al.,* 2025 |

**Table S4** Repellent properties of plant-derived biopesticides against *Solenopsis* spp**.**

| **Plant** | **Target** | **Repellency and other behavior effects** | **Reference** |
| --- | --- | --- | --- |
| *Hedychium* ssp. | *S. invicta* | **Digging behavior** (mg/kg)**:**   1. *H. hyrsiforme* EO repelled at 100 2. *H.* *elatum* EO at 1 and 10 | Sakhanokho *et al.,* 2013 |
| *Cinnamomum aromaticum* Nees | *S. invicta* | **Digging behavior (**24 h/repellency%/10 - 15 cm):   1. Minor workers: 74% 2. Major workers: 80% | Huang *et al.,* 2015 |
| *Murraya exotica* | *S. invicta* | **Grasping abilities** (fresh leaves):   1. Major workers: 87% to 7% 2. Minor workers: 95% to 0%   **Walking abilities** (fresh leaves)**:**   1. Major workers: 87% to 7% 2. Minor workers: 95% to 0%   **Aggregation rate** (fallen leaves):   1. Major workers: 87% to 17% 2. Minor workers: 68% to 3% | Zhang *et al.,* 2016 |
| Essential balm | *S. invicta* | **Foraging behavior**:   1. Completely inhibited (24 h) at 2 µl/cm^2^ 2. Increased particle transportation and be less defensive at 0.5, 1, 2 µl/cm^2^ | Wen *et al.,* 2016 |
| *Petasites fragrans*  (4-methoxy benzaldehyde) | *S. invicta* | **Olfactometer trials:**   1. 21% in treatment airspace vs 50% control | Pattrick *et al.,* 2017 |
| *Cupressus nootkatensis* | *S. invicta* (Buren) *S. richteri* (Forel) | **Digging behavior:**   1. No sand removed at 1, 10, 100 µl. In 1-3 months of 10 µl nootka oil a 5 and 28% digging amount vs control | Addesso *et al.,* 2017 |
| *Viburnum odoratissimum* | *S. invicta* | **Digging behavior** (10 days):   1. Major workers: 100% (0-5 cm)   **Walking abilities** (12 days)**:**   1. Major and minor workers: 100% (0-5cm)   **Grasping abilities** (9 days):   1. Major and minor workers: 96% and 100% (0-5cm) | Zhang *et al.,* 2017 |
| *Michelia alba*  (Fresh leaves) | *S. invicta* | **Aggregating** (From 64h to 6 h):   1. Major workers: 60% to 0% 2. Minor workers: 28% to 0%   **Drinking ability:**   1. Major workers: 23% to 3% 2. Minor workers: 5% to 1.7% | Qin *et al.,* 2018 |
| *Eutrema japonicum* (Miq.) Kiudz | *S. invicta* Buren | **Bait traps**:   1. Ordinary polyethylene film (control-40 min) at 157 ± 45 ants vs baits with microencapsulated AITC films (6mg - 250 ppm AITC gas) at 0 ants. | Hashimoto *et al.,* 2019 |
| *Colchicum autumnale* L. | *S. invicta* | **Foraging behavior** (colony weight after 15 days/mg/L):   1. 59% Bulb powder (100) 2. 45% Colchicine (1)   **Aggregation rate** (15 days)**:**   1. 35% Bulb powder (100) 2. 49% Colchicine (1)   **Grasping rate:**   1. 17% Bulb powder (100) 2. 39% Colchicine (1)   **Walking speed:**   1. 2.0 cm/s Control 2. 0.7 cm/s Bulb powder (100) 3. 1.1 cm/s Colchicine (1) | Lin *et al.,* 2020 |
| *Litsea cubeba* | *S. invicta* | **Walking rate** (24 h/ 5.3 µL/mL)**:**   1. Major workers: 32% *L.cubeba* oil, 16% citral 2. Minor workers: 16% *L.cubeba* oil, 0% citral   **Climbing rate** (24 h/ 5.3 µL/mL)**:**   1. Major workers: 20% *L.cubeba* oil, 16% citral 2. Minor workers: 0% *L.cubeba* oil and citral | Xiao *et al.,* 2020 |
| *Gelsemium elegans* | *S. invicta* | **Aggregation rate** (reduced in %):   1. 57% (1/100 leaf extract) 2. 60% (1/100 stem extract) 3. 60% (0.5 mg/kg koumine)   **Climbing rate** (reduced in %)**:**   1. 60% (1/100 leaf extract) 2. 58% (1/100 stem extract) 3. 58% (0.5 mg/kg koumine)   **Walking rate:**   1. 1.5cm/s (1/100 leaf extract) 2. 1.6 cm/s (1/100 stem extract) 3. 1.5 cm/s (0.5 mg/kg koumine) | Zheng *et al.,* 2020 |
| *Citrus paradisi* | *S. invicta* | **Walking rate:**   1. 20% workers (α-Pinene)   **Gripping ability** (5µL/tube)**:**   1. 13%- 25% workers (Octanal, α- pinene, and β-phellandrene) | Zhang *et al.,* 2021 |
|  | *S. invicta* | **Foraging behavior** (24 h)**:**   1. Eucalyptol, camphor, menthol, and methyl salicylate (all at 2 mg/cm^2^) triggered particle covering behavior 2. Eugenol, under field conditions, effectively suppressed the foraging behaviors | Wen *et al.,* 2021 |
| *Magnolia grandiflora* L. | Hybrids (*S. invicta/S. richteri*) | **Digging behavior** (19.5 µg/g)**:**   1. 0.06 g Sand removed (seed EO) vs 1.2g control 2. 0.28g Sand removed (1-Decanol) vs 1.6g control 3. 0.8g Sand removed (1-Octanol) vs 1.7g control | Ali *et al.,* 2022 |
| *Osmanthus fragrans* (Thunb.) Lour.  *Sophora japonica* Linn, *Eugenia caryophμllata* Thunb.,  *Ligustrum compactum* (Wall. exG. Don) Hook.f.,  *Jasminum sambac* (L.) Ait., *Allium polyrhizum* Turcz. Ex Regel | *S. invicta Buren* | **Olfactometer trials** (μg/ml):   1. *O. fragans* EO (200): Minor workers: 73% repellent effect. Major workers 64% repellent effect 2. *S.japonica* EO: Minor and major workers: 66% attraction rates (200). Male ants: 63% repellent rate (8) 3. *E. caryophμllata* EOs: Minor and major workers: 66% repellent rate (40) and 63% attraction rate (2) 4. *L. compactum* EO: Minor workers: 69 % repellent rate (1000) and 66% attraction rate (2). Major workers: 63 % repellent rate (200) and 69 % attractive rate (40) 5. *J.samba*c EO: Minor workers: 65% attraction rate (40). Male ants 63% repellent effect (1000) 6. *A. polyrhizum* EO: Minor workers: 63 % attraction rate (200) and 65% repellent rate (1000) | Li *et al.,* 2022 |
| *Curcuma longa* | *S. invicta, S. richteri, Hybrids* | **Digging behavior** (removed sand/ 19.5 µg/g):   1. *S. invicta*: 0.04g leaf oil vs 1.80g control, 0.36g rhizome oil vs 1.86g control 2. *S. richteri*:0.31g Leaf oil vs 1.90g control, 0.18g rhizome oil (156 µg/g) vs 1.16g control 3. *Hybrids*: 0.58g Leaf oil vs 1.64g control, 0.11g rhizome oil vs 2.02g control, 0.78g Ar-turmerone vs 21.9g control | Ali *et al.,* 2024 |
| *Baccharis microdonta* | *S. invicta, S. richteri, Hybrids* | **Digging behavior**  **(**EO shows repellency at):   1. *S. invicta*: 5 µg/g 2. *S. richteri*: 5 µg/g 3. *Hybrids*:39 µg/g   (Removed sand/24-h/19.5 µg/g):   1. *S. invicta*: 0.61g EO vs 1.80g control 2. *S. richteri*: 0.003 EO vs 1.20g control 3. *Hybrids*: 0.59 EO vs 2.25 control | Ali *et al.,* 2023 |
| *Lippia gracilis* |  | **Self-cleaning**   1. Increased self-cleaning and lower allogrooming and aggregation at 3.42 µg/mg of *L. gracilis* EO 2. Carvacrol and carvacryl benzoate at LD_30_ increase distance and speed walked with 27.28% and 38.47% rise compared to the control (817.7 mm) | Dantas *et al.,* 2023 |
| *Illicium verum, Blumea balsamifera, Acorus tatarinowii* | *S. invicta* | **Aggregation:**   1. *B.balsamifera* and *I. verum* EOs suppressed at 0.001-0.01µL/cm^3^ 2. *A. tatarinowii* EOs suppressed at 0.005 and 0.01 µL/cm^3^   **Aggressiveness:**   1. ~20% reduction (36 h) with *A. tatarinowii, I. verum*, and *B. balsamifera* (0.001 µL/cm^3^)   **Grasping ability**:   1. *I. verum* (0.001 µL/cm^3^) significantly reduced to 24h | Fu *et al.,* 2023 |
| Eugenol derivatives | *S. invicta* | **Foraging behavior** (1 hr/0.5 mg/cm^2^):   1. Fewer foraging ants and shorter duration of food transportation with methyl isoeugenol, isoeugenol, eugenol acetate, methyl eugenol, or eugenol vs controls and isoeugenyl acetate 2. Significant particle relocation for methyl isoeugenol (2 mg/cm^2^)   **Digging behavior (**sand digging**)**:   1. Complete suppression at 500 mg/kg of methyl isoeugenol, isoeugenol, methyl eugenol, and eugenol 2. Few nesting activities were found in flowerpots treated with methyl isoeugenol | He *et al.,* 2023 |
| *Houttuynia cordata* Thunb | *S. invicta* | **Digging behavior**:   1. 0.5 gr of sand removed with 125 µg/g of *H. cordata* vs ~2 g control 2. 1 gr of sand removed with 62.5µg/g of 2-undecanone vs ~2 g control | Kurmanbayeva *et al.,* 2023 |
| *Cymbopogon nardus* and Peppermint | *S. invicta* | 90% repellency | Lao *et al.,* 2023 |
| *Celastrus angulatus* | *S. invicta* | **Knockdown times (KT_50_)- 300 *S. invicta* workers:**   1. Azadirachtin: 5.963 min (500 µg/ml); 23.825 min (10 µg/ml) 2. Celangulin**:** 7.061 min (500 µg/ml); 19.447 min (10 µg/ml) | Liang *et al.,* 2023b |
|  | *S. invicta, S. richteri, Hybrids* | **Digging behavior** (removed sand):   1. Carvacrol: 0.98, 7.8, and 0.98 µg/g against *S. invicta, S. richteri, Hybrids* 2. Thymol: 31.25, 31.25, and 7.8 µg/g against *S. invicta, S. richteri, Hybrids* 3. Carvacrol acetate: 31.25, 31.25, and 15.6 µg/g *S. invicta, S. richteri, Hybrids* 4. Thymol acetate: 62.5, 31.25, and 125 µg/g against *S. invicta, S. richteri, Hybrids* | Paudel *et al.,* 2023 |
| *Matricaria chamomilla* cv. Egyptian *(1), M.chamomilla* German CO2 *(2), M.chamomilla* cv. German (3) | *S. invicta, S. richteri, Hybrids* | **Digging behavior** (removed sand):   1. Repellency (1) at 7.8, 7.8, and 31.25 µg/g against *S. invicta, S. richteri, Hybrids* 2. Repellency (2) at 125–15.6 µg/g against *S. invicta*, and 15.6–3.9 µg/g against *S. richteri, Hybrids* 3. Repellent at (3) 3.9, 7.8, and 31.25 µg/g against *S. invicta, S. richteri, Hybrids* 4. Repellency of α-bisabolol at 7.8, 7.8, and 31.25 µg/g *S. invicta, S. richteri, Hybrids* | Shah *et al.,* 2023 |
| Garlic | *S. invicta* | **Grasping ability** (exposure 2-12 h/ 16 µg/ml)**:**   1. Major workers: 15 to 1.8% vs 90 to 82% (control) 2. Minor workers: 13 to 0% vs 92 to 82% (control)   **Walking behaviour:**   1. Major workers: 22 to 0% vs 98 to 93% (control) 2. Minor workers: 20 to 0% vs 98 to 97 % (control) | Song *et al.,* 2023 |
| *Sophora flavencens* | *S. invicta* | **Foraging behavior** (5 days):   1. Matrine: 97% (control) vs 71% (15mg/L). 2. Sophocarpine: 97% (control) vs 74% (15mg/L)   **Foraging behavior** (*T. molitor* rates consumption on day 12):   1. Matrine: 0.14 mg/ant (control) vs 0.06 mg/ant (15 mg/L) 2. Sophocarpine: 0.16 mg/ant (control) vs 0.07 mg/ant (15 mg/L)   **Aggregation behavior:**   1. Matrine: 85% (control) vs 55% (15.0 mg/L) 2. Sophocarpine: 90% (control) vs 61% (15.0 mg/L) | Tian *et al.,* 2023 |
| *Cinnamomum loureirii; Cinnamomum cassia* | *S. invicta* | **Leg-antennal grooming behaviour:**   1. *C. loureirii* EO bark: 88 times⋅2 min^-1^, leaf: 101 times⋅2 min^-1^ 2. *C. cassia* EO bark: 72 times⋅2 min^-1^, leaf: 50 times⋅2 min^-1^ 3. *Trans*-cinnamaldehyde: 77 times⋅2 min^-1^   **Double-antennal grooming behaviour:**   1. *C. loureirii* EO bark: 56 times⋅ 2 min^-1^, leaf: 56 times⋅2 min^-1^ 2. *C. cassia* EO bark: 55 times⋅2 min^-1^, leaf: 41 times⋅2 min^-1^ 3. *Trans*-cinnamaldehyde: 76 times⋅2 min^-1^   **Figthing behavior** (maximum fighting rate)**:**   1. *C. loureirii* bark oil: 20.00% ± 0.00 2. *C. cassia* bark oil: 16.67% ± 2.72 3. *Trans*-cinnamaldehyde: 58.33% ± 3.60 | Xing *et al.,* 2023b |
| *Cinnamomum loureirii* | *S. invicta* | **Grasping effect** (320 µg/cm^3^):   1. *C. loureirii* leaf EO: decreased to 0% (10 min) 2. *C. loureirii* bark EO: decrease to 0% (50 min)   **Knockdown effect** (320 µg/cm^3^):   1. *C. loureirii* leaf knocks down all ants (10 min) 2. *C. loureirii* bark knocks down all ants (50 min) | Xing *et al.,* 2023a |
| *Artemisia argyi* Levl. et Vant.,  *Aquilaria sinensis* (Lour.) Gilg,  *Cymbopogon citratus* (DC.) Stapf,  *Mentha canadensis* Linnaeus, *Pogostemon cablin* (Blanco) Benth,  *Santalum album* L.,  *Thymus mongolicus* (Ronniger) Ronniger,  *Zingiber officinale* Roscoe | *S. invicta* | **Digging behavior:**   1. All EOs (1% concentration) showed significant supression (all P < 0.001), with *A. argyi* and *S. album* EOs having the strongest effects ~1% (24 h) 2. Dipentene, α-terpineol, 1-caryophyllene, linalool, 2 phenylethanol, and benzyl acetate showed significant effects ~1% against (all P < 0.001) (2ml)   **Climbing behavior:**   1. All EOs (1 mL, <20%, 60 s) and individual compounds of *A. argyi* and *S. album* reduce climbing in Y-type arm board | Fan *et al.,* 2024 |
| *Salvia miltiorrhiza, Perilla frutescens,Pogostemon cablin, Schizonepeta tenusfolia, Mentha canadensis* | *S. invicta* | **Digging behavior** (μg/ml)**:**  *S. miltiorrhiza* EO:   1. Minor workers (repellency rate): 65% (1000) 2. Major workers (repellency rate): 65% (1000) 3. Male (attractive rate): 63% (1000) 4. Virgin queen (attractive rate) 70% (2)   *P. frutescens* EO:   1. Minor workers (repellency rate): 65% (1000) 2. Major workers: 73% (1000) (repellency rate) 3. Major workers: 61% (2) (attractive rate) 4. Virgin queens (repellency rate) ~70% (2-1000)   *P. cablin* EO:   1. Minor workers (repellency rate): 64% (1000) 2. Major workers (repellency rate): 65% (1000) 3. Virgin queens (repellency rate): ~70% (2-8)   *S. tenusfolia* EO:   1. Minor worker (repellency rate) 85% (1000) 2. Major workers (repellency rate): 68% (1000)   *M. canadensis* EO:   1. Minor workers (repellency rate) 78% (1000) 2. Major workers (repellency rate): 63% (1000) 3. Male ants (repellency rate) 68% (1000) 4. Virgin queens (repellency rate): 64% (1000)   *Individual compounds:*   1. Minor workers (repellency rate): menthone: 88% (1000), menthol: 78% (1000), caryophyllene oxide: 74% (1000) 2. Major workers: 3-methylcyclopentanol: 70% (1000) (repellency rate), eugenol: 78% (1) (attractive rate) 3. Virgin queens: eugenol: 74% (1) (attractive rate), borneol: 64% (0.1) (attractive rate) | Wang *et al.,* 2024 |
| *Kaempferia galanga, Rosmarinus officinalis* | *S. invicta* | **Foraging behavior:**   1. *K. galanga* crude extract (50 mg/mL-1 d) demonstrated the most effective non-selective antifeedant activity rate of 52.67% 2. *R. officinalis* crude extract showed the best selective antifeedant activity rates of 69.33 % (50 mg/mL) and 56.33 % (10 mg/mL) | Guo *et al.,* 2025 |
| Seriphidiums pp. | *S. invicta* | **Digging behavior**   1. All the selected species except *S. badghysum* and *S. balchanorum* showed significant repellent activities of 55–95% at 2.5μL/mL. | Rizvi *et al.,* 2025 |

**Table S5** Biopesticides based on plants as inhibitors of AChE enzyme

| **Plant species** | **Compound or Essential oil** | **concentration** | **Inhibition** | **Target (Organism)** | **Reference** |
| --- | --- | --- | --- | --- | --- |
| *Azadirachta indica* | Azadirachtin | 0.5 ppm | Decreased AchE activity (µmol/min/mg protein) to 0.65 vs 1.15 control | Brown planthopper (*Nilaparvata lugens*) | Nathan *et al.,* 2007 |
|  | *A. indica* extract | 1.0 µg/mL | % Inhibition 55.7 ± 11.9 (Adult) | Golden Apple Snail (*Pomacea canaliculata*) | Zaib *et al.,* 2024 |
| *Illicium verum* | *I.verum* ethyl acetate extract | 1.0 mg/mL | 62.8 % (24 h) | Aphididae (*Myzus persicae*) | Zhou *et al.,* 2016 |
|  | Carvacrol; *I.verum* essential oil |  | IC_50_ (mg/mL): 0.029 ± 0.004; 0.117 ± 0.002 | Spotted wing drosophila (*Drosophila suzukii*) | De Souza *et al.,* 2022 |
|  | *I. verum* essential oil | 1% | Decreased AchE activity (mmoles/min/mg protein) to ~0.075 vs 0.45 control | Litter beetle (*Alphitobius diaperinus*) | Peter *et al.,* 2022 |
| *Blumea balsamifera* | Blumeatin | 136 ± 12.6 µM | 36 ± 0.04% Inhibition; K_i_= 413 ± 46.2 µM | Electric eel *(Electrophorus electricus)* | Acero and Amor, 2022 |
| *Acorus tatarinowii* | *A. tatarinowii* volatile oil | 150 µM | 80% |  | Xie *et al.,* 2024 |
| *Citrus limon* | *Citrus limon* essential oil | 10,000 µl/L | 0.34 ± 0.16 mU/mg vs ~2.5 mU/mg control for *C. chinensis* and 1.67 ± 0.07 mU/mg vs ~6 mU/mg control for *C. maculatus* | Pulse beetle (*Callosobruchus chinensis* / *Callosobruchus maculatus*) | Gupta *et al.,* 2025 |
|  | *Citrus limon* essential oil | EC_50_= 164.35 µL/L |  |  | Oboh *et al.,* 2014 |
| *Cinnamomum cassia* | *Cinnamomum* cassia essential oil | 3.81 mg/mL | 125U/L vs 150 U/L control | *Haemaphysalis longicornis* | Nwanade *et al.,* 2024 |
|  | *Cinnamomum* cassia essential oil | 0.66 mg/mL | 10 U/mg prot vs 30 U/mg prot control | *Haemaphysalis longicornis* | Bai *et al.,* 2024 |
| *Citrus paradisi* | Nootkatone/auraptene | 1.62 µg/mL | Inhibition of AChE of 17.6% vs 23.1% control | AchE (bovine erythtocytes) | Miyazawa *et al.,* 2001 |
| *Allium sativum* | *A. sativum* essential oil | 80% of 24-h LC50 (0.26 µl/cm3) | AChE activity reduced to 30.9% | *Tribolium castaneum* | Chaubey, 2013 |
|  | *A. sativum* essential oil |  | IC_50_ = 43.5 µg/mL | AchE (bovine erythtocytes) | Akinyemi *et al.,* 2018 |
|  | *A. sativum* essential oil | 80% of 24-h LC50 (0.30 µl/cm3) | 0.0462±0.0009(52.20) vs 0.0886±0.0019(100) Control | *Sitophilus oryzae* | Chaubey, 2016 |
| *Litsea cubeba* | *L. cubeba* essential oil | 0.1 µL/cm2 | AChE level (0 days): 1505.70 U/g vs AChE level (1 days): 224.73 U/g | *Drosophila suzukii* | Pan *et al.,* 2022 |
| *Seriphidium brevifolium* | *S. brevifolium* essential oil*/*1,8-cineole | For both it was used LD30, been <LC50 = 16.47 µl/l (*S. brevifolium* EO)/ <LC50 = 19.11 µl/l (1,8-cineole) | AChE activity (mg/protein/min) 0.6 control vs 0.2 for SBEO and 1,8-Cineole | *Solenopsis invicta* | Xie *et al.,* 2020 |
| *Cupressus nootkatensis* | Carvacrol; Nootkatone |  | IC_50_ (µM Carvacrol):  a) *M. domestica* 1638  b) *D. variabilis* 224  c) *P. americana* 51  d) >30, 000; IC50 (µM Nootkatone): all >30, 000; | House fly (*Musca domestica*); American dog tick (*Dermacentor variabilis*); American cockroach (*Periplaneta americana*); Yellow fever mosquito (*Aedes aegypti*) | Anderson *et al.,* 2012 |
| *Matricaria chamomilla* | *M. chamomilla var. recutita* essential oils from Bursa and Istanbul | 10 mg/mL | AChE inhibition (%): a) 11.9 ± 1.7 (Bursa); b) 4.7 ± 0.6 (Istanbul) | *Sitophilus granarius*, *S. oryzae* (Coleoptera: Curculionidae), *Tribolium confusum*, and *T. castaneum* (Coleoptera: Tenebrionidae). | Alkan *et al.,* 2024 |
| *Curcuma longa* | *C. longa* essential oil |  | IC_50_ (mg/mL): 95.10 ± 1.25 | electric eel *(Electrophorus electricus)* | Tamfu *et al.,* 2020 |
| *Baccharis microdonta* | Carvacrol |  | IC_50_ (µg/mL): 40.00 ± 0.05 | *Drosophila melanogaster* | Muñoz-Nuñez *et al.,* 2025 |
| *Houttuynia cordata* | *H. cordata* essential oil (areial parts and underground stem) | 100 µg/mL | Enzyme activity (picomole of acetylcholine iodide hydrolyzed/min/mg of protein): 0.36 ± 0.01 | Male Swiss albino mice | Verma *et al.,* 2017 |
| *Artemisia annua* | *Artemisia annua* (post-flowering phase) |  | AChE inhibitory activity IC_50_= 0.13 ± 0.02 mg/mL | Electric eel *(Electrophorus electricus)* | Yu *et al.,* 2011 |
|  | *Artemisia annua* ethanol extract | 500 µg/mL | 71.83 ± 7.32 %; IC_50_ (µg/mL): 87.43 ± 4.04 |  | Chougouo *et al.,* 2016 |
| *Viburnum odoratissimum* | Compounds 1-4, and 7 (ursonic acid); ursolic acid (4), 6α-hydroxyl-20(29)-en-3-on-28-oic acid (3), split-ring iridoids (1–2) |  | IC_50_ values from 1.284 ± 0.277 to 2.090 ± 0.095 µM |  | Liang *et al.,* 2023a |
| *Cnidium monnieri* | Cindimine, imperatorin | 100 µg/mL | % Inhibition:  Cindimine (80.4%), imperatorin (71.4%); <50% Aloperine, Osthole, Xanthotoxin, Isoimperatorin;  <40% Isopimpinellin, marmesin;  <20% bergapten | Pine Wood Nematode (*Bursaphelenchus xylophilus*) | Feng *et al.,* 2023 |
| *Piper aduncum* | *P. aduncum* essential oil; dillapiole |  | Application of LC_50_ = 3.49 *P. aduncum* essential oil and LC_50_ = 3.38 dillapiole significantly reduce AChE activity | *Amblyomma sculptum* (Acari: Ixodidae) | Pereira Filho *et al.,* 2024 |
| *Eucalyptus globulus Labill.* | *E. globulus* essential oil | 2.16 ± 0.19 (mg GALAE/g) GALAE: Galantamine equivalent | Significant inhibition | *Helicoverpa armigera* | Kobenan *et al.,* 2022 |
| *Syzygium aromaticum* | *S. aromaticum* methanol extract; *S. aromaticum* essential oil; eugenol |  | AChE inhibition IC_50_ value (μg/ml); *S. aromaticum* methanol extract (61.5 ± 1.88); *S. aromaticum* essential oil (49.73 ± 1.33); eugenol (42.44 ± 1.21) | AChE from bovine erythrocytes | Dalai *et al.,* 2014 |
| *Coriandrum sativum* | *C. sativum* essential oil |  | IC_50_ (mg/mL): 0.68 ± 0.03 |  | Hajlaoui *et al.,* 2021 |
| *Colchicum autumnale* L. | *Colchicum autumnale* alkaloid extract (corms) |  | % Inhibition: 14.82 ± 0.64 | Electric eel *(Electrophorus electricus)* | Dincheva *et al.,* 2025 |
| *Gelsemium elegans* | *G. elegans* leaf extract |  | Increased AChE activity (U/mg prot): 75 control vs 150 leaf extract in 48 h | Fire ant | Zheng *et al.,* 2024 |
| *Mentha spicata* | IC_50_ (µg/mL) or % inhibitory activity: a) 1,8-cineole: 41 µg/mL; b) (-)-carvone: 43% (164 µg/mL); c) (-)-linalool: 37% (164 µg/mL); d) (-)-limonene: 27% (164 µg/mL) |  | IC_50_ (µg/mL):  a) *M. spicata* (self-pollinated Oranda-hakka): 37;  b) M. spicata (Mesidai-Ke-hakka): 57;  c) M. spicata (native spearmint): 88;  d) M. spicata (Soren-hakka): 130 |  | Miyazawa *et al.,* 1998 |
|  | *M. spicata* essential oil |  | IC_50_ (µg/mL) or % inhibitory activity:  *Mentha spicata* 372.7 µg/mL 60%;  Alpha-pinene 495.7 µM <90%;  Limonene 2532.2 µM <90% |  | Chu *et al.,* 2025 |
|  | *M. spicata* essential oil*,* linalool, and carvone | *M. spicata:* 20 µL/mL; linalool: 6.0 µL/mL; carvone 6.0 µL/mL | IC_50_ (µg/mL) or % inhibitory activity:  *M. spicata* 3.228 µL/mL 80% (20 µL/mL); Linalool 0.14 µL/mL 90% (6.0 µL/mL)  Carvone 1.92 µL/mL ~55% (6.0 µL/mL) | *Reticulitermes dabieshanensis* | Wu *et al.,* 2023 |
| *Acorus calamus* | *A. calamus* rhizomes hydroalcoholic extract and *A. calamus* rhizomes essential oil |  | IC_50_ (µg/mL):  A  *A. calamus* rhizomes hydroalcoholic extract 182.31 ± 16.78  *A. calamus* rhizomes essential oil 10.67 ± 0.81 |  | Mukherjee *et al.,* 2007 |
| *Cymbopogon citratus* | *C. citratus* leaf extract | 200 mg/mL | AChE activity (µmol/min/ mg protein): 1.7 control vs 0.6 *C. citratus* leaf extract | *Drosophila melanogaster* | Johnson *et al.,* 2021 |
| *Ruta graveolens* | *R. graveolens* hexane extract | 400 µg/mL | *R. graveolens* aqueous extract IC_50_ = 50 µg/mL; *R. graveolens* hexane extract IC_50_ = 34 µg/mL, 94.9 ± 2.1 % |  | Luo *et al.,* 2024 |
| *Lavandula angustifolia* | *Lavandula angustifolia* essential oil | 10 mg/mL | AChE inhibition of 65% | Electric eel *(Electrophorus electricus)* | Talić *et al.,* 2023 |
| *Melaleuca alternifolia* | *M. alternifolia* essential oil | 40 mg/ml | AChE activity (U/mg prot) from 0.325 control to 0.175 *M. alternifolia* essential oil | *Helicoverpa armigera* | Liao *et al.,* 2017 |
|  | *M. alternifolia essential oil* |  | IC_50_= 96.4 ± 0.98 µg/mL | AChE horse serum | Alfred Ngenge *et al.,* 2021 |

**Table S6** Biopesticides based on plants as inhibitors of GST enzyme

| **Plant specie** | **Compound or Essential oil** | **Concentration** | **Effect** | **Target (Organism)** | **Reference** |
| --- | --- | --- | --- | --- | --- |
| *Azadirachta indica* | Neem seed extract | 35 mg L^-1^ | Control 40.60 ± 0.00 vs 80.10 ± 0.10 mM CDNB conjugated product/mg protein/min (increased) | Silkworms *(Bombyx mori)* | Rattanapan and Sujayanont, 2024 |
| *Illicium verum* | Ethyl acetate extract | 1 mg/mL (24 h treatment) | Inhibition 60.34% | Aphididae (*Myzus persicae*) | Zhou *et al.*, 2016 |
|  | Essential oil | 0.50% | Nmol/min/mg of protein: Control 0.5 vs 5.5 *I. verum* essential oil | Panzer (*Alphitobius diaperinus*) | Peter *et al.*, 2022 |
|  | Fruit extracts (ethyl acetate extract) | 20 mg/L | Control ~45.00 vs 20.00 U\mg prot *I. verum* extract | *Sitophilus zeamais* | Li *et al.*, 2013 |
|  | Essential oil | 0.001 µL/cm^3^ (24 h) | Control 7.5 vs 17.5 U/g | *Solenopsis invicta* Buren | Fu *et al.*, 2023 |
| *Blumea balsamifera* | Essential oil | 0.001 µL/cm^3^ (24 h) | Control 7.5 vs 15 U/g | *Solenopsis invicta* Buren | Fu *et al.*, 2023 |
| *Acorus tatarinowii* | Essential oil | 0.001 µL/cm^3^ (24 h) | Control 7.5 vs ~13 U/g | *Solenopsis invicta* Buren | Fu *et al.*, 2023 |
| *Citrus limon* | Essential oil | 0.001 µL/cm^3^ (24 h) | Control 7.5 vs ~10 U/g | *Solenopsis invicta* Buren | Fu *et al.*, 2023 |
| *Mosla chinensis* | Essential oil | 0.001 µL/cm^3^ (24 h) | Control 7.5 vs ~13.75 U/g | *Solenopsis invicta* Buren | Fu *et al.*, 2023 |
| *Cinnamomum cassia* | Essential oil | 0.001 µL/cm^3^ (24 h) | Control 7.5 vs 12.5 U/g | *Solenopsis invicta* Buren | Fu *et al.*, 2023 |
| *Allium sativum* | Dipropyl disulfide (DPDS), propyl methyl sulfide (PMDS), diallyl disulfide (DADS) | 300 µM (48 h) | GST activity [nmol/min/mg protein]: Control 10, DPDS 25, PMDS 25, DADS 40 | GTSPP in rat clone 9 cells | Tsai *et al.*, 2011 |
|  | Essential oil | 2.801 µl/ml (24 h) | GST activity [U/mg protein): 0.268 ± 0.009 Control vs 0.438 ± 0.0007 | *Ephestia kuehniella* | Shahriari *et al.*, 2020 |
| *Seriphidium brevifolium* | (α+β) Thujone | 12 µl/l | GST activity [CDNB product/mg/ protein/min): ~1.3 vs ~0.9 | *Solenopsis invicta* | Xie *et al.*, 2020 |
| *Matricaria chamomilla* | Essential oil, nanoemulsion | EO: 2327 mg/L, NE: 134 mg/L | GST activity [mmole sub. conjugated/min/mg protein]: 4 control vs 5.8 EO and 5.4 nanoemulsion | *Aphis craccivora* | Abdelaal *et al.*, 2021 |
| *Artemisia annua* | Essential oil | 0.693 % (24h); 0.419 % (48 h) | GST activity [CDNB/OD/min: 0.12 vs 0.16 (24 h); 0.18 vs 0.08 (48 h); GST activity [DCNB/OD/min]: 0.25 vs 0.1 (24 h) | *Pseudococcus viburni* (3rd nymphal instars) | Ramzi *et al.*, 2017 |
|  | Essential oil | LC_30_= 6.07 mg/g, LC_50_= 3.86 mg/g | GST [μmol · min^–1^ · mg^–1^ protein]: 0.148 ±0.007 control vs 0.094 ± 0.009 (LC_30_), 0.0116 ± 0.001 (LC_50_) | *Helicoverpa armigera* (4th instar larva) | Mojarab-Mahboubkar *et al.*, 2015 |
| *Sophora flavecens* | Alkaloid matrine (MT), alkaloid oxymatrine (OMT), 1,8-cineole (CN) | 0.4 mg/ml | GST activity [nmol/min/g]: 2750 control vs 4000 MT/OMT (8/2)/CN (12 h); 3500 control vs ~2000 MT/OMT (8/2)/CN and ~2500 CN (48 h) | *Plutella xylostella* | Xu *et al.*, 2023 |
| *Cinnamomum aromaticum* | *Cinnamomum aromaticum* essential oil (Grain treatment) | 15 % (24 h) | GST activity [µmol/min/mg protein]: ~25 control vs 5 | *Tribolium castaneum* (larvae) | Tarigan and Harahap *et al.*, 2016 |
| *Piper aduncum* | Essential oil, dillapiole | *Piper aduncum* EO: 3.49 mg/ml, dillapiole: 3.38 mg/ml | GST activity [µU/µg protein]: ~5 control vs ~15 *P. aduncum* EO, ~2 control vs ~20 dillapiole | *Amblyomma sculptum* | Pereira Filho *et al.*, 2024 |
| *Eucalyptus globulus Labill.* | Essential oil | 6.111 µl/ml (24 h) | GST activity [U/mg protein): 0.268 ± 0.009 control vs 0.405 ± 0.0007 | *Ephestia kuehniella* | Shahriari *et al.*, 2020 |
| *Ilex chinensis Sims.* | Essential oil | 43.57 µg/cm2 (24 h) | GST activity [U/gprot]: ~2.0 control vs 1.5 *I. chinensis* essential oil | *Haemaphysalis longicornis* | Gao *et al.*, 2022 |
| *Pinus massoniana Lamb.* | *α-*Pinene | 2.8 mg/g (Low dose-LD); 5.6 mg/g (medium dose-MD); 11.2 (high dose-HD) (24 h, 48 h) | GST Activity [U/mg prot]: ~100 control vs ~300 (LD), ~500 (MD), ~700 (HD) (24 h); ~100 control vs ~300 (LD), ~300 (MD), ~500 (HD) (48 h); | *Monochamus alternatus* | Xue *et al.*, 2023 |
| *Syzygium aromaticum* | Essential oil | 14.45 µg/cm^2^ (24 h) | GST Activity [U/gprot]: ~2.0 control vs 1.375 *S. aromaticum* essential oil | *Haemaphysalis longicornis* | Gao *et al.*, 2022 |
| *Mentha spicata* | Limonene | 1.683 µl/L (24 h) | GST Activity [U/mg prot]: ~30 control vs 33 limonene | *Reticulitermes dabieshanensis* | Yang *et al.*, 2021 |
|  | Essential oil | 18.422 µl/ml | GST Activity [µmol/min/mg protein]: 0.142 ±0.0107 control vs 0.504 ±0.0050 | *Tribolium castaneum* | Heydarzade *et al.*, 2019 |
| *Acorus calamus* | Methanol extract | Feeding deterrence (FD): FD_50_= 6.18 µg/cm^2^ (4h) | GST Activity [Glutathione conjugated product/min/mg in nM]: 8.68 ± 0.48 control vs 6.01 ±0.24 | *Spodoptera litura* (third instar larvae) | Kumrungsee *et al.*, 2023 |
|  | Essential oil | 25 µl/ml | GST Activity [nmol/min/mL]: 13.2 ± 1.1 negative control vs 7.1 ±0.2 | *Phenacoccus solenopsis* | Shoba *et al.*, 2024 |
| *Eugenia caryophyllus* | Essential oil | a) 5 mg/kg, b) 10 mg/kg (24 h after last intraperitoneal injection) | GST Activity [1,2-dinitro-4-nitrobenzene nmol/mg protein/min]: 208.6 ± 11.43 control vs a) 123.3 ± 10.97; b) 117.6 ± 9.42 | Spague-Dawley male rats | Park, 2006 |
| *Cymbopogon citratus* | Essential oil | Diluted with 50% ethanol to sublethal concentrations of 1% and 2% (24 h) | *HlGST* mRNAs increased more than 6 times (6.47 ± 0.39) than the control group | *Haemaphysalis longicornis* | Agwunobi *et al.*, 2020 |
|  | Permethrin+ β-citral (pβ),  Permethrin+ α-citral (pα) | 0.57 µg/larva (pβ), 0.42 µg/larva (pα) | GST Activity [CDNB µmol/mg protein/min]: 0.18 ± 1.77 control vs 0.56 ± 1.58 (pβ); 0.14 ± 1.47 (pα) | *Spodoptera frugiperda* | Ismail, 2024 |
| *Ruta graveolens* | Essential oil | LC_50_ = 27.21 ppm | GST Activity [µM/min/mg protein]: 2.448 ± 0.305 control vs 4.177 ± 0.256 (24 h); 3.093 ± 0.537 control vs 7.042 ± 0.752 (48 h) | *Culiseta longiareolata* (larvae -L4) | Bendjazia *et al.*, 2025 |
| *Lavandula angustifolia* | *L. angustifolia* essential oil | LC_25_ = 85.90 µl/liter (24 h) | GST Activity [nM/mg of proteins]: 27.64 ± 0.98 control vs 42.78 ± 0.32 (24 h); 28.99 ± 1.24 control vs 38.24 ± 0.35 (48 h) | *Rhyzopertha dominica* | Sayada *et al.*, 2022 |
| *Angelica sinensis* | Extract of *A. sinensis* | Diets containing 4.0 g ethyl acetate extract of *Angelica sinensis* (EAE) kg^−1^ diet for 60 days | GST Activity [U/mg protein]: 45.26 ± 2.08 control vs 54.86 ± 3.90 EAE | Gills and erythrocytes in trichlorfon-treated carp (*Cyprinus carpio* var. Jian) | Li *et al.*, 2020 |
| *Melaleuca alternifolia* | *M, alternifolia* essential oil | LD_50_ = 25.140 mg/ml | GST Activity [U/mg protein]: ~190 control vs ~160 M, alternifolia EO (12 h) | *Helicoverpa armigera* | Liao *et al.*, 2017 |

**References:**

Abdelaal, K., Essawy, M., Quraytam, A., Abdallah, F., Mostafa, H., Shoueir, K. *et al.* (2021) Toxicity of essential oils nanoemulsion against *Aphis craccivora* and their inhibitory activity on insect enzymes. *Processes*, 9(4), 624.

Acero, R. E. P., and Amor, E. C. (2022) Cholinesterase Inhibitory Activities and *In Silico* Docking Studies of Blumeatin Isolated from *Blumea balsamifera* L. DC. *Philippine Journal of Science*, 15(2).

Addesso, K. M., Oliver, J. B., O’Neal, P. A., and Youssef, N. (2017) Efficacy of nootka oil as a biopesticide for management of imported fire ants (Hymenoptera: Formicidae). *Journal of Economic Entomology*, 110(4), 1547-1555.

Agwunobi, D. O., Pei, T., Yang, J., Wang, X., Lv, L., Shen, R. *et al.* (2020) Expression profiles of glutathione S-transferases genes in semi-engorged *Haemaphysalis longicornis* (Acari: Ixodidae) exposed to *Cymbopogon citratus* essential oil. *Systematic and Applied Acarology*, 25(5), 918-930.

Akinyemi, A. J., Faboya, L., Awonegan, A., Olayide, I., Anadozie, S., and Oluwasola, T. (2018). Antioxidant and anti-Acetylcholinesterase activities of essential oils from garlic (*Allium sativum*) Bulbs. *Int. J. Plant Res*, *31*(2).

Alfred Ngenge, T., Kucukaydin, S., Ceylan, O., and Duru, M. E. (2021) Evaluation of enzyme inhibition and anti-quorum sensing potentials of *Melaleuca alternifolia* and *Citrus sinensis* essential oils. *Natural Product Communications*, 16(9), 1934578X211044565.

Ali, A., Chen, J., and Khan, I. A. (2022) Toxicity and repellency of *Magnolia grandiflora* seed essential oil and selected pure compounds against the workers of hybrid imported fire ants (Hymenoptera: Formicidae). *Journal of Economic Entomology*, 115(2), 412-416.

Ali, A., Shah, F. M., Manfron, J., Monteiro, L. M., de Almeida, V. P., Raman, V., and Khan, I. A. (2023) *Baccharis* Species essential oils: Repellency and toxicity against yellow fever mosquitoes and imported fire ants. *Journal of Xenobiotics*, 13(4), 641-652.

Ali, A., Shah, F. M., Radwan, M. M., Elhendawy, M. A., Elsohly, M. A., and Khan, I. A. (2024) *Curcuma longa* essential oils: toxicity and repellency against imported fire ants (Formicidae: Hymenoptera). *Journal of Medical Entomology*, 61(1), 191-200.

Alkan, M., Servi, H., Karakoç, Ö. C., Ertürk, S., Yücel, Y. Y., and Polatoğlu, K. (2024) Insecticidal and AChE inhibitory activities of *Matricaria chamomilla* var. recutita essential oils collected from different regions against storage insect pests. *Journal of Stored Products Research*, 109, 102439.

Anderson, J. A., and Coats, J. R. (2012) Acetylcholinesterase inhibition by nootkatone and carvacrol in arthropods. *Pesticide Biochemistry and Physiology*, 102(2), 124-128.

Bai, L., Gao, Z., Xu, X., Lv, W., Wang, Y., Dong, K. *et al.* (2024) The acaricidal activity and enzymatic targets of the essential oils of *Cinnamomum cassia* and *Cinnamomum camphora* and their major components against *Haemaphysalis longicornis* (Acari: Ixodidae). *Industrial Crops and Products*, 209, 117967.

Bendjazia, R., Dris, D., Seghier, H., Essid, R., Jallouli, S., Tabbene, O., and Bouabida, H. (2025) Analysis of the chemical properties and larvicidal activity of essential oils from *Ruta graveolens* and *Ruta montana* against *Culiseta longiareolata*: Toxicity, enzymatic and biochemical alterations. *South African Journal of Botany*, 180, 512-519.

Chaubey, M. K. (2013) Insecticidal effect of *Allium sativum* (Alliaceae) essential oil. *Journal of Biologically Active Products from Nature*, 3(4), 248-258.

Chougouo, R. D., Nguekeu, Y. M., Dzoyem, J. P., Awouafack, M. D., Kouamouo, J., Tane, P. *et al.* (2016) Anti-inflammatory and acetylcholinesterase activity of extract, fractions and five compounds isolated from the leaves and twigs of *Artemisia annua* growing in Cameroon. *Springerplus*, 5, 1-7.

Chu, M. H., Liu, T. W., Chen, P. H., Chen, Y. H., Tang, K. L., Hsu, S. J. *et al.* (2025) Investigation of the acetylcholinesterase inhibitors of Mentha genus essential oils with in vitro and in silico approaches. *Industrial Crops and Products*, 227, 120783.

Dalai, M. K., Bhadra, S., Chaudhary, S. K., Bandyopadhyay, A., and Mukherjee, P. K. (2014) Anti-cholinesterase activity of the standardized extract of *Syzygium aromaticum* L. *Pharmacognosy magazine*, 10(2), S276.

Dantas, J. O., Cavalcanti, S. C., Araújo, A. P. A., Blank, A. F., Silva, J. E., Picanço, M. C. *et al.* (2023) Synthetic carvacrol derivatives for the management of *Solenopsis* ants: toxicity, sublethal effects, and horizontal transfer. *Agriculture*, 13(10), 1988.

De Souza, L., Cardoso, M. D. G., Konig, I. F. M., Ferreira, V. R. F., Caetano, A. R. S., Campolina, G. A. *et al.* (2022) Toxicity, histopathological alterations and acetylcholinesterase inhibition of *Illicium verum* essential oil in *Drosophila suzukii*. *Agriculture*, 12(10), 1667.

Dincheva, I., Badjakov, I., Georgiev, V., Semerdjieva, I., Vrancheva, R., Ivanov, I., and Pavlov, A. (2025) Comprehensive GC-MS Characterization and Histochemical Assessment of Various Parts of Three Colchicum Species from Bulgarian Flora. *Plants*, 14(2), 270.

Du, Y., Grodowitz, M. J., and Chen, J. (2020) Insecticidal and enzyme inhibitory activities of isothiocyanates against red imported fire ants, *Solenopsis invicta*. *Biomolecules*, 10(5), 716.

Fan, M., Ye, T., Wang, Z., Li, Q., Li, C., Shi, Q. *et al.* (2024) The repellent effects of eight Chinese herbal essential oils on red imported fire ants, *Solenopsis invicta* Buren (Hymenoptera: Formicidae), and analysis of active components. *Industrial Crops and Products*, 219, 119111.

Feng, J., Qin, C., Liu, X., Li, R., Wang, C., Li, C. *et al.* (2023) Nematicidal coumarins from *Cnidium monnieri* fruits and *Angelica dahurica* roots and their physiological effect on pine wood nematode (*Bursaphelenchus xylophilus*). *Molecules*, 28(10), 4109.

Fu, J., Ma, Z., Wang, L., Zhang, Y., and Luo, Y. (2023) Fumigant toxicity and behavioral alterations of six plant essential oils against the red fire ant (*Solenopsis invicta* Buren). *Environmental Science and Pollution Research*, 30(26), 68677-68690.

Gao, Z., Yu, Z., Qiao, Y., Bai, L., Song, X., Shi, Y. *et al.* (2022) Chemical profiles and enzyme-targeting acaricidal properties of essential oils from *Syzygium aromaticum*, *Ilex chinensis* and *Citrus limon* against *Haemaphysalis longicornis* (Acari: Ixodidae). *Industrial Crops and Products*, 188, 115697.

Guo, Y. B., Liu, Z. J., Ye, Y. Y., Chen, Y. Y., and Wu, Z. W. (2025). Antifeedant and contact toxicity activity of crude extracts from 10 plants to red imported fire ant, *Solenopsis invicta* Buren (Hymenoptera: Formicidae). Journal of Asia-Pacific Entomology, 28(1), 102350.

Gupta, H., Singh, P. P., and Reddy, S. E. (2025) Exploring the chemical profiling and insecticidal properties of essential oils from fresh and discarded lemon peels, *Citrus limon* against pulse beetle. *International Biodeterioration and Biodegradation*, 196, 105924.

Hajlaoui, H., Arraouadi, S., Noumi, E., Aouadi, K., Adnan, M., Khan, M. A. *et al.* (2021) Antimicrobial, antioxidant, anti-acetylcholinesterase, antidiabetic, and pharmacokinetic properties of *Carum carvi* L. and *Coriandrum sativum* L. essential oils alone and in combination. *Molecules*, 26(12), 3625.

Hashimoto, Y., Yoshimura, M., and Huang, R. N. (2019) Wasabi versus red imported fire ants: preliminary test of repellency of microencapsulated allyl isothiocyanate against *Solenopsis invicta* (Hymenoptera: Formicidae) using bait traps in Taiwan. *Applied entomology and zoology*, 54, 193-196.

He, Y., Zhang, J., Shen, L., Wang, L., Qian, C., Lyu, H. *et al.* (2023) Eugenol derivatives: Strong and long-lasting repellents against both undisturbed and disturbed red imported fire ants. *Journal of Pest Science*, 96(1), 327-344.

Heydarzade, A., Valizadegan, O., Negahban, M., and Mehrkhou, F. (2019) Efficacy of *Mentha spicata* and *Mentha pulegium* essential oil nanoformulation on mortality and physiology of *Tribolium castaneum* (Col.: Tenebrionidae). *Journal of Crop Protection*, 8(4), 501-520.

Huang, C. L., Fu, J. T., Liu Y. K., Cheng, D. M., Zhang, Z. X. (2015). The insecticidal and repellent activity of soil containing cinnamon leaf debris against red imported fire ant workers. Sociobiology, 62(1), 46-51.

Ismail, S. M. (2024) Synergistic Action of Lemongrass Oil and Permethrin on Biochemical Responses of *Spodoptera frugiperda* (JE Smith). *Journal of Agricultural Sciences and Engineering*, 6(3), 133-140.

Johnson, T. O., Ojo, O. A., Ikiriko, S., Ogunkua, J., Akinyemi, G. O., Rotimi, D. E. *et al.* (2021) Biochemical evaluation and molecular docking assessment of *Cymbopogon citratus* as a natural source of acetylcholine esterase (AChE)-targeting insecticides. *Biochemistry and Biophysics Reports*, 28, 101175.

Kobenan, K. C., Ochou, G. E. C., Kouadio, I. S., Kouakou, M., Bini, K. K. N., Ceylan, R. *et al.* (2022) Chemical composition, antioxidant activity, cholinesterase inhibitor and in vitro insecticidal potentiality of essential oils of *Lippia multiflora* Moldenke and *Eucalyptus globulus* Labill. on the main carpophagous pests of cotton plant in Ivory Coast. *Chemistry and Biodiversity*, 19(4), e202100993.

Kumrungsee, N., Wiwattanawanichakun, P., Phankaen, P., Saiyaitong, C., Koul, O., Nobsathian, S. *et al.* (2023) Phenolic secondary metabolites from *Acorus calamus* (Acorales: Acoraceae) rhizomes: The feeding deterrents for *Spodoptera litura* (Lepidoptera: Noctuidae). *Journal of economic entomology*, 116(5), 1613-1620.

Kurmanbayeva, A., Ospanov, M., Tamang, P., Shah, F. M., Ali, A., Ibrahim, Z. M. *et al.* (2023) Regioselective Claisen–Schmidt Adduct of 2-Undecanone from *Houttuynia cordata* Thunb as Insecticide/Repellent against *Solenopsis invicta* and Repositioning Plant Fungicides against *Colletotrichum fragariae*. *Molecules*, 28(16), 6100.

Lao, G. M., Dasmariñas, H. A. Q., Nangcas, J. M. M., Luna, M. M., Perocho, S. N. S., Valdez, A., and Fegarido, J. E. (2023). Citronella (*Cymbopogon Nardus*) and peppermint (*Mentha x Piperita*) oil extracts as ant-repelling spray. ASEAN Journal of Agriculture and Food Engineering, 2(1), 33-38.

Li, H. T., Wu, M., Wang, J., Qin, C. J., Long, J., Zhou, S. S. *et al.* (2020) Protective role of *Angelica sinensis* extract on trichlorfon-induced oxidative damage and apoptosis in gills and erythrocytes of fish. *Aquaculture*, 519, 734895.

Li, S. G., Li, M. Y., Huang, Y. Z., Hua, R. M., Lin, H. F., He, Y. J. *et al.* (2013) Fumigant activity of Illicium verum fruit extracts and their effects on the acetylcholinesterase and glutathione S-transferase activities in adult *Sitophilus zeamais*. *Journal of pest science, 86, 677-683.*

Li, Y., Yu, S., Huang, J., Wang, Z., Zeng, Y., Wu, X. *et al.* (2022) Study of behavioral, electrophysiological response, and the active compounds of the essential oils from six kinds of flowers against *Solenopsis invicta* Buren (Hymenoptera: Formicidae). *Industrial Crops and Products*, 188, 115603.

Liang, J. J., Lv, T. M., Xu, Z. Y., Du, N. N., Lin, B., Huang, X. X., and Song, S. J. (2023a) Two new iridoids and triterpenoid analogues from the leaves of *Viburnum chingii* and their anti-acetylcholinesterase activity. *Fitoterapia*, 165, 105400.

Liang, Y., Liang, M., Chen, H., Hong, J., Song, Y., Yue, K., and Lu, Y. (2023b) The Effect of Botanical Pesticides Azadirachtin, Celangulin, and Veratramine Exposure on an Invertebrate Species *Solenopsis invicta* (Hymenoptera: Formicidae). *Toxins*, 16(1), 6.

Liao, M., Xiao, J. J., Zhou, L. J., Yao, X., Tang, F., Hua, R. M. *et al.* (2017) Chemical composition, insecticidal and biochemical effects of *Melaleuca alternifolia* essential oil on the *Helicoverpa armigera*. *Journal of Applied Entomology*, 141(9), 721-728.

Lin, S., Qin, D., Zhang, Y., Zheng, Q., Yang, L., Cheng, D. *et al.* (2020) Toxicity and sublethal effects of autumn crocus (*Colchicum autumnale*) bulb powder on red imported fire ants (*Solenopsis invicta*). *Toxins*, 12(11), 731.

Luo, P., Feng, X., Liu, S., and Jiang, Y. (2024) Traditional Uses, Phytochemistry, Pharmacology and Toxicology of Ruta graveolens L.: A Critical Review and Future Perspectives. *Drug Design, Development and Therapy*, 6459-6485.

Miyazawa, M., Hideyukitougo, and Ishihara, M. (2001) Inhibition of acetylcholinesterase activity by essential oil from *Citrus paradisi*. *Natural Product Letters*, 15(3), 205-210.

Miyazawa, M., Watanabe, H., Umemoto, K., and Kameoka, H. (1998) Inhibition of acetylcholinesterase activity by essential oils of Mentha species. *Journal of Agricultural and Food Chemistry*, 46(9), 3431-3434.

Mojarab-Mahboubkar, M., Sendi, J. J., and Aliakbar, A. (2015) Effect of *Artemisia annua* L. essential oil on toxicity, enzyme activities, and energy reserves of cotton bollworm *Helicoverpa armigera* (Hübner)(Lepidoptera: Noctuidae). *Journal of Plant Protection Research*, 55(4).

Mukherjee, P. K., Kumar, V., Mal, M., and Houghton, P. J. (2007) *In vitro* acetylcholinesterase inhibitory activity of the essential oil from *Acorus calamus* and its main constituents. *Planta medica*, 73(03), 283-285.

Muñoz-Nuñez, E., Madrid-Villegas, A., Alarcón-Enos, J., Ferreira-Funes, C., Valdés-Navarro, F., Santander, R. *et al.* (2025) Insecticidal potential of *Baccharis macraei* essential oils: An ecological approach to their volatile composition in insect management. *Agronomy*, 15(3), 509.

Nalini, T., and Sasinathan, S. (2020). Evaluation of toxicity of botanicals and essential oils against *Solenopsis geminata* (Fabricius)(Hymenoptera: Formicidae). Eco. Env. Cons, 26, 210-215.

Nathan, S. S., Choi, M. Y., Seo, H. Y., Paik, C. H., Kalaivani, K., and Kim, J. D. (2008) Effect of azadirachtin on acetylcholinesterase (AChE) activity and histology of the brown planthopper *Nilaparvata lugens* (Stål). *Ecotoxicology and Environmental Safety*, 70(2), 244-250.

Nwanade, C. F., Wang, M., Yu, Z., and Liu, J. (2024) Biochemical and molecular mechanisms involved in the response of *Haemaphysalis longicornis* (Acari: Ixodidae) to *Cinnamomum cassia* essential oil and its major constituent. *Journal of Pest Science*, 97(1), 99-111.

Oboh, G., Olasehinde, T. A., and Ademosun, A. O. (2014) Essential oil from lemon peels inhibit key enzymes linked to neurodegenerative conditions and pro-oxidant induced lipid peroxidation. *Journal of oleo science*, 63(4), 373-381.

Pan, Z., Liu, S., Chen, Y. C., Wang, Y. D., Gu, Q., and Song, D. (2022) As natural phytocide: Biomarker assessment of *Litsea cubeba* (Lour.) Persoon essential oil against *Drosophila suzukii* Matsumura (Diptera: Drosophilidae.). *Industrial Crops and Products*, 187, 115421.

Park, H. J. (2006) Toxicological studies on the essential oil of *Eugenia caryophyllata* buds. *Natural Product Sciences*, 12(2), 94-100.

Pattrick, J. G., Shepherd, T., Hoppitt, W., Plowman, N. S., and Willmer, P. (2017). A dual function for 4-methoxybenzaldehyde in *Petasites fragrans*? Pollinator-attractant and ant-repellent. Arthropod-Plant Interactions, 11, 623-627.

Paudel, P., Shah, F. M., Guddeti, D. K., Ali, A., Chen, J., Khan, I. A., and Li, X. C. (2023). Repellency of carvacrol, thymol, and their Acetates against Imported Fire Ants. Insects, 14(10), 790.

Pereira Filho, A. A., do Vale, V. F., de Oliveira Monteiro, C. M., Barrozo, M. M., Stanton, M. A., Yamaguchi, L. F. *et al.* (2024) Effects of *Piper aduncum* (Piperales: Piperaceae) essential oil and its main component dillapiole on detoxifying enzymes and acetylcholinesterase activity of *Amblyomma sculptum* (Acari: Ixodidae). *International Journal of Molecular Sciences*, 25(10), 5420.

Peter, R., Josende, M. E., da Silva Barreto, J., da Costa Silva, D. G., da Rosa, C. E., and Maciel, F. E. (2022) Effect of *Illicium verum* (Hook) essential oil on cholinesterase and locomotor activity of *Alphitobius diaperinus* (Panzer). *Pesticide Biochemistry and Physiology*, 181, 105027.

Qin, D., Huang, R., Li, Z., Wang, S., Cheng, D., and Zhang, Z. (2018) Volatile component analysis of *Michelia alba* leaves and their effect on fumigation activity and worker behavior of *Solenopsis invicta*. *Sociobiology*, 65(2), 170-176.

Ramzi, S., Seraji, A., Azadi Gonbad, R., Mirhaghparast, S. K., Mojib Haghghadam, Z., and Haghighat, S. (2017) Toxicity of *Artemisia annua* (Asteraceae) essential oil on the tea mealy bug, *Pseudococcus viburni* Sigornet (Hemiptera: Pseudococcidae). *Archives of Phytopathology and Plant Protection*, 50(19-20), 941-956.

Rattanapan, A., and Sujayanont, P. (2024) Impact of Neem Seed Extract on Mortality, Esterase and Glutathione-S-Transferase Activities in Thai Polyvoltine *Hybrid Silkworm*, *Bombyx mori* L. *Insects*, 15(8), 591.

Rizvi, S. A. H., Li, Y., Ullah, R. M. K., and Lu, Y. (2025) Exploring the fumigant potential of *Artemisia* subg. *Seriphidium* essential oils and their dominant constituents against the red imported fire ants *Solenopsis invicta*. *Industrial Crops and Products*, *226*, 120603.

Sakhanokho, H. F., Sampson, B. J., Tabanca, N., Wedge, D. E., Demirci, B., Baser, K. H. C. *et al.* (2013) Chemical composition, antifungal and insecticidal activities of Hedychium essential oils. *Molecules*, 18(4), 4308-4327.

Sayada, N., Tine, S., and Soltani, N. (2008) Evaluation of a botanical insecticide, lavender (*Lavandula angustifolia* (M.)) essential oil as toxicant, repellent and antifeedant against lesser grain borer (*Rhyzopertha dominica* (F.)). *Appl. Ecol. Environ. Res*, 20, 1301-1324.

Seixas, P. T. L., Demuner, A. J., Alvarenga, E. S., Barbosa, L. C. A., Marques, A., Farias, E. D. S., and Picanço, M. C. (2018) Bioactivity of essential oils from Artemisia against *Diaphania hyalinata* and its selectivity to beneficial insects. *Scientia Agricola*, 75, 519-525.

Shah, F. M., Guddeti, D. K., Paudel, P., Chen, J., Li, X. C., Khan, I. A., and Ali, A. (2023) *Matricaria chamomilla* essential oils: Repellency and toxicity against imported fire ants (Hymenoptera: Formicidae). *Molecules*, 28(14), 5584.

Shahriari, M., Zibaee, A., Shamakhi, L., Sahebzadeh, N., Naseri, D., and Hoda, H. (2020) Bio-efficacy and physiological effects of *Eucalyptus globulus* and *Allium sativum* essential oils against *Ephestia kuehniella* Zeller (Lepidoptera: Pyralidae). *Toxin reviews*, 39(4), 422-433.

Shoba, P., Rajagopal, R., Alfarhan, A., and Nandhakumari, P. (2024) Evaluation of the insecticidal activity of *Acorus calamus* rhizome and *Chrysopogon zizanioides* roots essential oils against mealybug, *Phenacoccus solenopsis*. *Discover Life*, 54(1), 1-12.

Song, Z., Wang, Y., Li, C., Tan, Y., Wu, J., and Zhang, Z. (2023) Fumigant toxicity and behavioral inhibition of garlic against red imported fire ants (*Solenopsis invicta*). *Environmental Science and Pollution Research*, 30(1), 1889-1897.

Souto, R. N. P., Harada, A. Y., Andrade, E. H. A., and Maia, J. G. S. (2012) Insecticidal activity of Piper essential oils from the Amazon against the fire ant *Solenopsis saevissima* (Smith)(Hymenoptera: Formicidae). *Neotropical entomology*, 41, 510-517.

Talić, S., Odak, I., Boras, M. M., Smoljan, A., and Bevanda, A. M. (2023). Essential oil and extracts from *Lavandula angustifolia* Mill. cultivated in Bosnia and Herzegovina: Antioxidant activity and acetylcholinesterase inhibition. *International Journal of Plant Based Pharmaceuticals*, *3*(1), 95-103.

Tang, L., Sun, Y. Y., Zhang, Q. P., Zhou, Y., Zhang, N., and Zhang, Z. X. (2013). Fumigant activity of eight plant essential oils against workers of red imported fire ant, *Solenopsis invicta*. Sociobiology, 60(1), 35-40.

Talić, S., Odak, I., Boras, M. M., Smoljan, A., and Bevanda, A. M. (2023) Essential oil and extracts from *Lavandula angustifolia* Mill. cultivated in Bosnia and Herzegovina: Antioxidant activity and acetylcholinesterase inhibition. *International Journal of Plant Based Pharmaceuticals*, 3(1), 95-103.

Tamfu, A. N., Ceylan, O., Kucukaydin, S., and Duru, M. E. (2020) HPLC-DAD phenolic profiles, antibiofilm, anti-quorum sensing and enzyme inhibitory potentials of *Camellia sinensis* (L.) O. Kuntze and Curcuma longa L. *LWT*, 133, 110150.

Tarigan, S. I., and Harahap, I. S. (2016) Toxicological and physiological effects of essential oils against *Tribolium castaneum* (Coleoptera: Tenebrionidae) and *Callosobruchus maculatus* (Coleoptera: Bruchidae). *Journal of Biopesticides*, 9(2), 135.

Tian, Y., and Zhang, Z. (2023) Insecticidal Activities of *Sophora flavescens* Alt. towards red imported fire ants (*Solenopsis invicta* Buren). *Toxins*, 15(2), 105.

Tsai, C. W., Liu, K. L., Lin, C. Y., Chen, H. W., and Lii, C. K. (2011) Structure and function relationship study of allium organosulfur compounds on upregulating the pi class of glutathione S-transferase expression. *Journal of agricultural and food chemistry*, 59(7), 3398-3405.

Verma, R. S., Joshi, N., Padalia, R. C., Singh, V. R., Goswami, P., Kumar, A. *et al.* (2017) Chemical Composition and Allelopathic, Antibacterial, Antifungal, and Antiacetylcholinesterase Activity of Fish‐mint (*Houttuynia cordata* Thunb.) from India. *Chemistry and biodiversity*, 14(10), e1700189.

Wang, G., Zhou, H., Yu, S., Wang, Z., Zeng, Y., Wu, X. *et al.* (2024) Behavioral preferences of *Solenopsis invicta* Buren to essential oils and active compounds from amiaceae plants. *Industrial Crops and Products*, 214, 118471.

Wen, Y., Ma, T., Chen, X., Liu, Z., Zhu, C., Zhang, Y. *et al.* (2016) Essential balm: a strong repellent against foraging and defending red imported fire ants (Hymenoptera: Formicidae). *Journal of Economic Entomology*, 109(4), 1827-1833.

Wu, Z., Jin, C., Chen, Y., Yang, S., Yang, X., Zhang, D., and Xie, Y. (2023) Mentha spp. essential oils: A potential toxic fumigant with inhibition of acetylcholinesterase activity on *Reticulitermes dabieshanensis*. *Plants*, 12(23), 4034.

Xiao, C. X., Tan, Y. T., Wang, F. F., Wu, Q. H., Qin, D. Q., and Zhang, Z. X. (2020) The Fumigating Activity of *Litsea cubeba* oil and Citral on *Solenopsis invicta*. *Sociobiology*, 67(1), 41-47.

Xie, F., Rizvi, S. A. H., and Zeng, X. (2020) Fumigant toxicity and biochemical properties of (α+ β) thujone and 1, 8-cineole derived from *Seriphidium brevifolium* volatile oil against the red imported fire ant *Solenopsis invicta* (Hymenoptera: Formicidae). *Revista Brasileira de Farmacognosia*, 29, 720-727.

Xie, W., Li, J., Zhang, W., Sun, Q., and Chen, L. (2024) Inhibitory Effect of Acorus tatarinowii Nasal in Situ Gel on Acetylcholinesterase. *Advances in Engineering Technology Research*, 10(1), 347-347.

Xing, H., Hu, Y., Yang, L., Lin, J., Bai, H., Li, Y. *et al.* (2023a). Fumigation activity of essential oils of *Cinnamomum loureirii* toward red imported fire ant workers. *Journal of pest science*, 96(2), 647-662.

Xing, H., Lin, J., Li, X., Huang, J., Liang, X., Li, Y. *et al.* (2023b) Changes in dopamine and octopamine levels caused disordered behaviour in red imported fire ants exposed to cinnamon essential oils. *Industrial Crops and Products*, 199, 116801.

Xu, J., Lv, M., Fang, S., Wang, Y., Wen, H., Zhang, S., and Xu, H. (2023) Exploration of synergistic pesticidal activities, control effects and toxicology study of a monoterpene essential oil with two natural alkaloids. *Toxins*, 15(4), 240.

Xue, M., Xia, X., Deng, Y., Teng, F., Zhao, S., Li, H. *et al.* (2023) Identification and Functional Analysis of an Epsilon Class Glutathione S-Transferase Gene Associated with α-Pinene Adaptation in *Monochamus alternatus*. *International Journal of Molecular Sciences*, 24(24), 17376.

Yang, X., Han, H., Li, B., Zhang, D., Zhang, Z., and Xie, Y. (2021) Fumigant toxicity and physiological effects of spearmint (*Mentha spicata*, Lamiaceae) essential oil and its major constituents against *Reticulitermes dabieshanensis*. *Industrial Crops and Products*, 171, 113894.

Yu, Z., Wang, B., Yang, F., Sun, Q., Yang, Z., and Zhu, L. (2011) Chemical compositionand anti-acetyl cholinesterase activity of flower essential oils of *Artemisia annua* at different flowering stage. *Iranian journal of pharmaceutical research: IJPR*, 10(2), 265.

Zaib, N. A. N. M., Hashim, S. N., Hassan, W. R. M., Tay, C. C. (2024). Molluscicidal Activity and Inhibition of Acetylcholinesterase Activity of *Azadirachta indica* Extract on *Pomacea canaliculata*. *Sains Malaysiana*, *53*(5), 1081-1091.

Zhang, N., Liao, Y., Xie, L., Zhang, Z., and Hu, W. (2021) Using essential oils from *Citrus paradisi* as a fumigant for *Solenopsis invicta* workers and evaluating the oils’ effect on worker behavior. *Environmental Science and Pollution Research*, 28, 59665-59672.

Zhang, Y., Fu, J., Huang, C., Cheng, D., Huang, R., and Zhang, Z. X. (2017) Insecticidal Activity of the Soil in the Rhizosphere of *Viburnum odoratissimum* against *Solenopsis invicta* (Hymenoptera: Formicidae). *Sociobiology*, 64(1), 1-6.

Zhang, Z. X., Huang, R. L., Li, Z. H., Wang, S. Y., Fu, J. T., and Cheng, D. M. (2016) Insecticidal effect of volatile compounds from plant materials of *Murraya exotica* against red imported fire ant workers. *Sociobiology*, 63(2), 783-791.

Zheng, Q., Yang, L. P., Lin, S. K., Ma, Q. L., Qin, D. Q., and Zhang, Z. X. (2020) Insecticidal activity of the leaf and stem water extract of *Gelsemium elegans* against *Solenopsis invicta*. *Sociobiology*, 67(2), 232-238.

Zheng, Q., Yan, W., Zhu, S., Miao, X., Wu, J., Lin, Z. *et al.* (2024). Effects of *Gelsemium elegans* extract on the red fire ant: disruption of peritrophic membrane integrity and alteration of gut microbial diversity, composition, and function. *Journal of Pest Science*, 97(4), 2139-2155.

Zhou, B. G., Wang, S., Dou, T. T., Liu, S., Li, M. Y., Hua, R. M. *et al.* (2016) Aphicidal activity of *Illicium verum* fruit extracts and their effects on the acetylcholinesterase and glutathione S-transferases activities in *Myzus persicae* (Hemiptera: Aphididae). *Journal of insect science*, 16(1), 11.

Zhou, Y., Zhang, M. X., Sun, M., Wan, L. S., and Chen, J. S. (2024) Exploring bioactive molecules with fumigation toxicity against the red imported fire ant *Solenopsis invicta* Buren from commercial essential oils by GC–MS combined with chemometrics. *Industrial Crops and Products*, 209, 117993.
